# A protein-based cGAS-STING nanoagonist enhances T cell-mediated anti-tumor immune responses

**DOI:** 10.1038/s41467-022-33301-0

**Published:** 2022-09-28

**Authors:** Xuan Wang, Yingqi Liu, Chencheng Xue, Yan Hu, Yuanyuan Zhao, Kaiyong Cai, Menghuan Li, Zhong Luo

**Affiliations:** 1 School of Life Science, Chongqing University, Chongqing, 400044 P. R. China; 2 Key Laboratory of Biorheological Science and Technology, Ministry of Education, Chongqing University, Chongqing, 400044 P. R. China

**Keywords:** Cancer immunotherapy, Immunotherapy, Cancer microenvironment

## Abstract

cGAS-STING pathway is a key DNA-sensing machinery and emerges as a promising target to overcome the immunoresistance of solid tumors. Here we describe a bovine serum albumin (BSA)/ferritin-based nanoagonist incorporating manganese (II) ions and β-lapachone, which cooperatively activates cGAS-STING signaling in dendritic cells (DCs) to elicit robust adaptive antitumor immunity. Mn^2+^-anchored mannose-modified BSAs and β-lapachone-loaded ferritins are crosslinked to afford bioresponsive protein nanoassemblies, which dissociate into monodispersive protein units in acidic perivascular tumor microenvironment (TME), thus enabling enhanced tumor penetration and spatiotemporally controlled Mn^2+^ and β-lapachone delivery to DCs and tumor cells, respectively. β-lapachone causes immunogenic tumor cell apoptosis and releases abundant dsDNA into TME, while Mn^2+^ enhances the sensitivity of cGAS to dsDNA and augments STING signaling to trigger downstream immunostimulatory signals. The cGAS-STING nanoagonist enhances the tumor-specific T cell-mediated immune response against poorly immunogenic solid tumors in vivo, offering a robust approach for immunotherapy in the clinics.

## Abstract (web summary)

Manganese has a crucial role in cGAS-STING-mediated DNA sensing and has emerged as a STING agonist. Here the authors report the design and characterization of a nanosystem incorporating manganese ions and the chemotherapeutic drug β-lapachone, inducing T-cell mediated anti-tumor immune responses in preclinical cancer models.

## Introduction

Immunotherapy is a promising antitumor modality that has attracted substantial interest in recent years, which refers to a broad category of treatment capable of stimulating the endogenous immune system to elicit antitumor effects^1–3^. From a mechanistical perspective, the treatment efficacy of immunotherapies predominantly relies on the mobilization of tumor-specific effector T cells, during which the naive CD4 and CD8 T cells are activated through interacting with the antigen-bound Class II and Class I major histocompatibility complex (MHC-II and MHC-I) expressed on antigen-presenting cells (APCs)^4–6^. However, the application of immunotherapy for solid tumor treatment has thus far been challenging, as solid tumors could orchestrate a highly immunosuppressive state in the microenvironment to escape from the T cell-mediated immune elimination^7,8^. Specifically, tumor cells could profoundly interfere with the antigen-presenting process by inhibiting the maturation and activation of dendritic cells (DCs), which are the most potent professional APCs in human body^9–11^. The resultant DC dysfunction would lead to critical failures in T cell priming and eventually cause immune tolerance. Therefore, the restoration and stimulation of DC function have become a promising strategy to reinvigorate T cell-mediated antitumor immunity^12^.

The cyclic guanosine monophosphate–adenosine monophosphate synthase (cGAS)-stimulator of interferon gene (STING) signaling is an evolutionarily conserved DNA-sensing mechanism in human body and plays critical roles in regulating the crosstalk between tumor cells and ambient immune cells, which emerges as a promising target for improving the effectiveness of immunotherapy against solid tumors^13,14^. During a typical treatment process, dsDNA derived from tumor cells is captured by DCs and binds with the cytosolic cGAS dimers, thereby activating their enzymatic activity to catalyze the reaction between adenylate triphosphate (ATP) and guanosine triphosphate (GTP) to generate 2′3′ cyclic GMP–AMP (cGAMP)^15–17^. cGAMP then binds to the STING dimers to induce STING oligomerization and further activates the TANK-binding kinase 1 (TBK1)-interferon regulatory factor 3 (IRF3) axis to trigger various immunostimulatory signals especially type I interferon (IFN-I) in DCs, which would substantially facilitate the DC-mediated cross-priming of T cells to develop robust antitumor immunity^18,19^. Nevertheless, the actual efficacy of several STING agonists in clinical trials was modest at most, revealing multiple key issues for cGAS-STING activation in tumor-encased DCs^20^. Typically, the TME is a physiologically complex niche with diverse cell populations, which makes the DC-specific delivery of STING agonists highly challenging^21^. On the other hand, a minimal concentration threshold of tumor-derived dsDNA is required for efficient dsDNA sensing and may kinetically impede cGAS-STING activation in DCs^22^. Moreover, solid tumor tissues are characterized by dense extracellular matrix and high interstitial fluid pressure^23^, which severely limits the diffusion rates of formulated STING agonists into tumor stroma and undermines their potential immunotherapeutic efficacy. Taken together, these features highlight the necessity of rational STING agonist selection and strategic therapeutic design for effective cGAS-STING signaling in DCs of solid tumors^24^.

Manganese is an essential trace element in human body with well-understood toxicology and several oral Mn supplements have long been approved for preventing or treating Mn deficiency-related diseases^25–27^. Recent insights reveal that Mn^2+^ ion also has crucial roles in cGAS-STING-mediated DNA sensing, which has emerged as a novel STING agonist with clinical promise^28^. From a mechanistic perspective, Mn^2+^ could directly bind with cGAS to enhance their dsDNA-sensitivity and cGAMP-producing enzymatic activity, thus stimulating the downstream STING signaling even under low levels of tumor-derived dsDNA^29–31^. On the other hand, Mn^2+^-cGAMP complexation could substantially enhance the STING binding affinity of cGAMP to facilitate STING activation, which is two orders of magnitude higher than pristine cGAMP^32,33^.

In this work, we report a TME-activatable nanoassembly based on natural-occurring bovine serum albumin (BSA) and ferritin for spatiotemporally coordinated Mn^2+^ delivery to tumor-residing DCs and immunogenic apoptosis induction in tumor cells by exploiting the cGAS-STING stimulatory function of Mn^2+^ ions, which leads to cooperative cGAS-STING activation in DCs to elicit robust T cell-mediated antitumor immunity. Taking advantage of the multiple modification and metal-binding sites in the protein structure, the BSAs are first functionalized with mannose (Man) moieties for targeting DCs and then loaded with Mn^2+^ ions via a simple ion diffusion approach (BSA-Man@Mn^2+^). Meanwhile, ferritin moieties could not only encapsulate β-lapachone (Lap) in the central cavity (Ft-Lap) at high yield through hydrophobic interaction, but also bind to transferrin receptor 1 (TfR1) overexpressed on tumor cell membrane to enable targeted Lap delivery. The BSA-Man@Mn^2+^ and Ft@Lap is procedurally cross-linked using acid-responsive Schiff base linkers to afford physiologically stable nanoassemblies (BSA-Man@Mn^2+^-Ft@Lap), which could be rapidly disintegrated in the slightly acidic perivascular TME into highly diffusive singular protein units, leading to enhanced penetration into solid tumor tissues. Specifically, Ft@Lap would be selectively taken in by tumor cells via TfR1-mediated endocytosis to induce immunogenic apoptosis, thus releasing abundant tumor-derived dsDNA into the TME for the cGAS-mediated DNA sensing in DCs, while BSA-Man@Mn^2+^ is efficiently internalized by DCs via Man-mediated ligand-receptor binding and release Mn^2+^ ions after enzymatic lysosomal degradation to activate the cGAS-STING axis, eventually triggering the downstream immunostimulatory transcriptional activities. These functions of the protein-based cGAS-STING nanoagonist in the present study could act in a cooperative manner to enhance the DC-mediated cross-priming of antitumor effector T cells in TME and improve antitumor immune responses in poorly immunogenic tumor models, which provides emerging opportunities for enhancing the efficacy of immunotherapies against solid tumor indications in the clinics.

## Results

### Construction and characterization of the nanoplatform

**Fig. 1:**
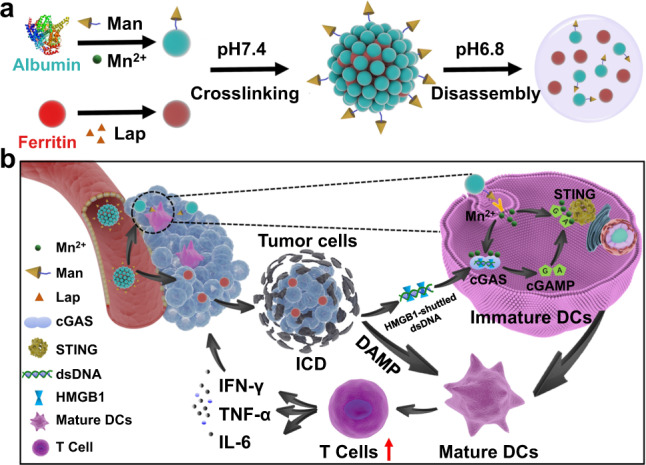
On-demand activation of the protein-based nanoassembly in TME enhances the immune response of poorly immunogenic solid tumors. **a** Preparation procedure of the protein nanoassembly and its activation in TME. **b** Schematic illustration of nanoassembly-mediated enhanced immunotherapy in poorly immunogenic solid tumors. The systemically administered BSA-Man@Mn^2+^-Ft@Lap nanoassembly rapidly disintegrated in the perivascular region of solid tumors into highly diffusive BSA-Man@Mn^2+^ and Ft@Lap components, thus enabling enhanced penetration into the tumor interior. Ft@Lap molecules would bind to the transferrin receptors on tumor cells for targeted Lap delivery to elicit efficient immunogenic apoptosis, leading to the release of abundant DAMPs including HMGB1 and dsDNA. Meanwhile, BSA-Man@Mn^2+^ molecules were selectively internalized by immature DCs to enhance the dsDNA sensitivity of the cGAS-STING axis. These activities would act in a cooperative manner to promote DC maturation and cross-priming, eventually enhancing the infiltration and effector function of tumor-specific T cells for efficient immunotherapy.

**Fig. 2:**
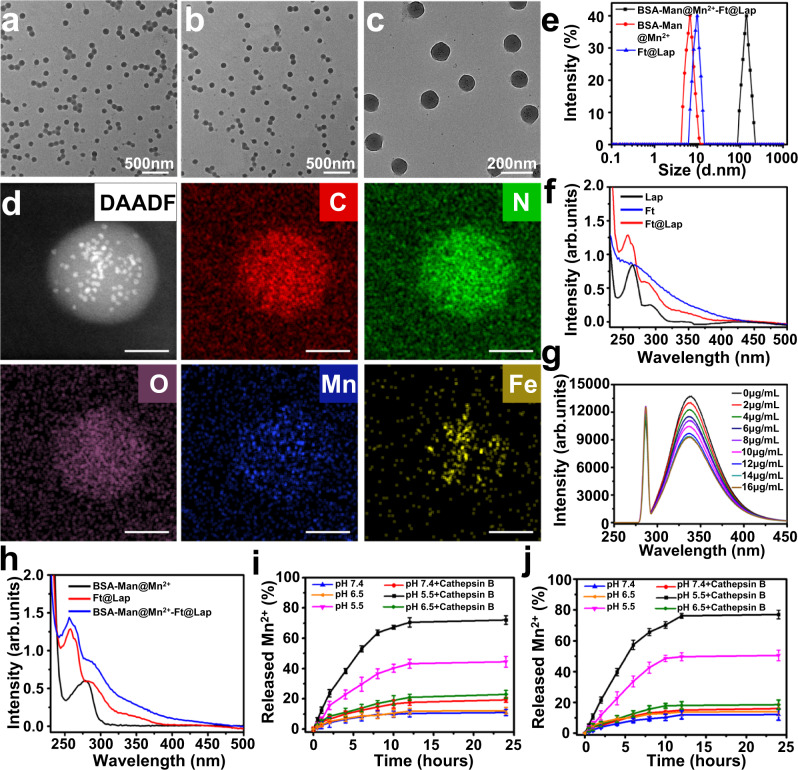
Physical characterization of the BSA-Man@Mn^2+^-Ft@Lap nanoassembly. **a** TEM image of blank BSA-Man-Ft nanocarriers. **b**, **c** TEM image of BSA-Man@Mn^2+^-Ft@Lap nanoassembly under low (**b**) and high (**c**) magnifications for illustrating the morphological details. **d** Scanning transmission electron microscopic (STEM) and EDS image of BSA-Man@Mn^2+^-Ft@Lap. **e** DLS analysis on the size distribution of Ft@Lap, BSA-Man@Mn^2+^, and BSA-Man@Mn^2+^-Ft@Lap in aqueous solution. **f** Comparative UV-vis absorption analysis of Lap, Ft, and Ft@Lap. **g** Changes in the fluorescence emission of BSA-Man@Mn^2+^ under different feeding ratios of Mn^2+^. Experiments in panels **a**–**g** were repeated three times independently with similar results. **h** Comparative UV-vis absorption analysis of Ferritin@Lap, BSA-Man@Mn^2+^, and BSA-Man@Mn^2+^-Ft@Lap. **i** Release profile of Mn^2+^ from BSA-Man@Mn^2+^-Ft@Lap. **j** The time-dependent release of Lap from BSA-Man@Mn^2+^-Ft@Lap after incubation under different conditions. Data are presented as mean values ± SEM (*n* = 3 independent experiments for panels **i**, **j**). Source data are provided as a Source data file.

To realize the spatiotemporally controlled amplification of cGAS-STING signaling in the complex TME, we formulated a biocompatible and TME-responsive protein-based nanostructure through the reversible cross-linking of bovine serum albumin (BSA) and ferritin (Fig. 1). Here BSA was employed as the carrier substrate for Mn^2+^ ions on account of the abundant metal ion binding sites therein. To improve DC targeting, the BSA molecules were proactively modified with mannose (Man) groups via non-invasive amide coupling reaction (BSA-Man), of which the process was thoroughly characterized via ^1^H NMR and HPLC in Supplementary Fig. 1. Fluorescence spectroscopic analysis of BSA after treatment with graded Mn^2+^ concentrations showed that the BSA-characteristic emission at 336 nm gradually decreased with increasing Mn^2+^ feeding ratios (Fig. 2g), which was ascribed to the binding between Mn^2+^ and aspartic acid or histidine residues in BSA and consistent with the observations in previous reports^34–36^. According to the DLS analysis, the hydrodynamic size of BSA-Man@Mn^2+^ was around 8 nm in aqueous solution (Fig. 2e). Meanwhile, β-lapachone (Lap), a clinically tested topoisomerase I/II inhibitor capable of inducing immunogenic tumor cell apoptosis, was accommodated into ferritin nanocages via hydrophobic interaction (Ft@Lap) by exploiting the large central cavity and intrinsic tumor cell binding capability of ferritin molecules, of which the size was maintained at around 10 nm. Uv-vis spectroscopic analysis of Ft@Lap revealed a new peak at 265 nm compared to pristine Ft and confirmed the effective loading of Lap into the ferritins (Fig. 2e, f). BSA-Man@Mn^2+^ and Ft@Lap were procedurally cross-linked using CHO-PEG_2000_-CHO as the bridging ligand via acid-responsive Schiff base bond, leading to the formation of physiologically stable nanoassemblies for robust drug delivery. Results of the TEM and DLS analysis collectively demonstrated that BSA-Man@Mn^2+^-Ft@Lap nanoassemblies had a uniform spherical shape with an average size of around 100 nm in Fig. 2a–c. The formation of BSA-Man@Mn^2+^-Ft@Lap was further confirmed by UV-vis spectroscopic analysis (Fig. 2h), of which the results showed that BSA-Man@Mn^2+^-Ft@Lap retained all the spectroscopic features of individual BSA-Man@Mn^2+^ and Ft@Lap components. The successful incorporation of Mn^2+^ ions and construction of the hybrid protein-based nanostructure was also supported by the results of energy dispersive X-ray spectroscopy (EDS) analysis (Fig. 2d). Quantitative inductively coupled plasma mass spectrometry (ICP-MS) and fluorescence spectroscopy tests further revealed that the loading ratios of Mn^2+^ and Lap in the final BSA-Man@Mn^2+^-Ft@Lap product were around 5.2% and 3.5%. To test the structural stability of the protein complex, BSA-Man@Mn^2+^-Ft@Lap was incubated in murine serum-supplemented PBS (10%) and its size changes were monitored via DLS. Only negligible changes were observed in the average diameter of the nanoassembly even after 48 h of incubation, of which the peak intensity and PDI both remained at the same level (Supplementary Fig. 2). The DLS data evidently suggests the favorable stability of the nanoassembly for drug administration via the intravenous route.

Taking advantage of the intrinsic hydrolysis capability of the Schiff base ligand between the BSA and Ft molecules, it’s theorized that the BSA-Man@Mn^2+^-Ft@Lap nanoassemblies could readily dissociate into smaller structural units in weakly acidic conditions, which is a key feature for realizing the cooperative modulation of DCs and tumor cells in the TME. As shown by the TEM images in Supplementary Fig. 3, it was observed that the globular BSA-Man@Mn^2+^-Ft@Lap nanoassemblies completely disintegrated into small protein units within 12 h under pH 6.5, which resembles the pH conditions of perivascular tissues in most solid tumors. We subsequently monitored the release kinetics of Mn^2+^ and Lap from the nanoassemblies using ICP and fluorescence spectroscopy. The release profiles in Fig. 2i showed that Mn^2+^ ions were well maintained within the BSAs under near-neutral conditions even in the presence of Cathepsin B, of which the final release ratios were only around 10.9%, 12.2%, 19.3%, and 22.6% after 24 h of incubation under pH 7.4, pH 6.5, pH 7.4+Cathepsin B, and pH 6.5+Cathepsin B, respectively. In contrast, the release rate of Mn^2+^ ions increased significantly at the lysosome-resembling acidic pH of 5.5 and was even higher under the combined treatment of pH 5.5 and Cathepsin B, which reached around 73% within only 12 h of incubation. The rapid Mn^2+^ release rate under pH 5.5+Cathepsin B was ascribed to the combined results of acidity-induced decoordination and Cathepsin B-induced enzymatic degradation of BSA. Similar release patterns have also been observed for the Fn-caged Lap in the nanoassemblies that the combined treatment of pH 5.5 and cathepsin B has triggered the fastest Lap release, validating their on-demand drug release capability in tumor lysosomes (Fig. 2j). The degradation and Mn^2+^/Lap release profiles of BSA-Man@Mn^2+^-Ft@Lap confirmed our hypothesis that the protein-based nanoassemblies could remain stable under physiological condition but disintegrate into highly diffusive protein units in TME, eventually release the payload in an on-demand manner in the lysosomal compartment of predetermined cellular targets. These merits may improve the specificity and efficacy of the immunomodulation treatment against solid tumors.

### Uptake analysis of the protein-based nanoassembly by target cells

**Fig. 3:**
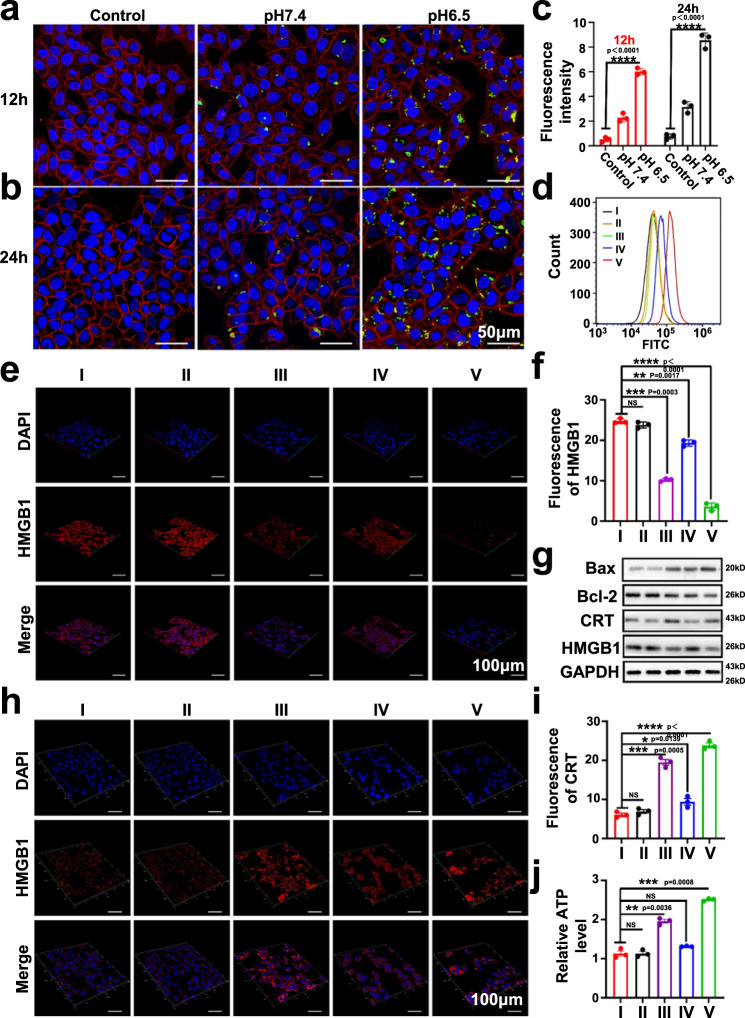
BSA-Man@Mn^2+^-Ft@Lap nanoassembly induced immunogenic apoptosis of tumor cells via Lap-mediated chemotherapy. **a**, **b** Fluorescence imaging on the endocytosis of BSA-Man@Mn^2+^-Ft-FITC@Lap by 4T1 tumor cells after incubation under different pH values for 12 h and 24 h. **c** Quantitative analysis of FITC fluorescence from panel a/b. **d** Flow cytometric analysis regarding the accumulation of mannose-modified BSA contents in DCs after 24 h of incubation with (I) control, (II) BSA-FITC@Mn^2+^-Ft@Lap at pH 7.4, (III) BSA-FITC-Man@Mn^2+^-Ft@Lap at pH 7.4, (IV) BSA-FITC@Mn^2+^-Ft@Lap at pH 6.5, and (V) BSA-FITC-Man@Mn^2+^-Ft@Lap at pH 6.5. **e** CLSM imaging of HMGB1 immunofluorescence in 4T1 cells after treatment with (I) control, (II) BSA-Man-Ft, (III) BSA-Man-Ft@Lap, (IV) BSA-Man@Mn^2+^-Ft, and (V) BSA-Man@Mn^2+^-Ft@Lap. Greater red fluorescence indicates lower HMGB1 release. **f** Quantitative fluorescence analysis of HMGB1 release in panel (**e**). **g** Western blot analysis on the expression levels of HMGB1, CRT and apoptosis markers (Bax, Bcl-2) in 4T1 cells with different treatments. **h** CLSM imaging of CRT expression after different treatments with (I) control, (II) BSA-Man-Ft, (III) BSA-Man-Ft@Lap, (IV) BSA-Man@Mn^2+^-Ft, and (V) BSA-Man@Mn^2+^-Ft@Lap. Greater red fluorescence indicates higher expression levels. **i** Quantitative fluorescence analysis of CRT in panel (**h**). **j** ATP levels in cell culture supernatants after 24 h of different treatments: (I) control, (II) BSA-Man-Ft, (III) BSA-Man-Ft@Lap, (IV) BSA-Man@Mn^2+^-Ft, and (V) BSA-Man@Mn^2+^-Ft@Lap. Data are presented as mean values ± SEM (*n* = 3 biologically independent samples for panels **c**, **f**, **i**, and **j**). Flow cytometry and western blot experiments in panels **d** and **g** were repeated three times independently with similar results. Statistical analysis for panels **c**, **f**, **i**, and **j** were carried out via multiple comparisons one-way ANOVA method. * Indicates significance at *p* < 0.05, ** indicates significance at *p* < 0.01, *** indicates significance at *p* < 0.001, **** indicates significance at *p* < 0.0001. Source data are provided as a Source data file.

The coordinated DC/tumor cell targeting capability of the BSA-Man@Mn^2+^-Ft@Lap nanoassemblies was investigated via fluorescence analysis under different pH conditions. To monitor the tumor-targeting ability of the nanoassembly, we first labeled Ft molecules with fluorescein isothiocyanate (FITC) and then cross-linked them with BSA-Man@Mn^2+^, which was further used to treat 4T1 tumor cells incubated at pH 7.4 and 6.5. Results of the CLSM imaging showed that fluorescence accumulation in tumor cells at pH 6.5 was visually higher than that under pH 7.4 (Fig. 3a–c). The distinctly different FITC fluorescence patterns could be readily explained by the acidity-triggered dissociation of the protein assembly under pH 6.5 and the resultant enhancement of the TfR-binding ability of Ft molecules, which could liberate those caged binding sites in their cross-linked state. Quantitative analysis showed that the mean fluorescence intensity (MFI) of FITC in the pH 6.5 group was 3 times higher than the pH 7.4 group. To elucidate the uptake mechanism of the Lap-laden Fn units, we employed TfR1 antibody to block the receptor-mediated endocytosis of tumor cells and monitored the changes in the uptake rate of the FITC-labeled Fn units under pH 6.5. As shown in Supplementary Fig. 4, the TfR1 antibody treatment caused a 67% decrease in the FITC fluorescence accumulation in BSA-Man-Ft-FITC@Lap-treated 4T1 cells under pH 6.5 (Supplementary Fig. 4a, b), validating our hypothesis that the Fn units could serve as tumor-targeted carriers for selective Lap delivery.

Based on similar mechanisms we also prepared FITC-labeled BSA to monitor the DC-targeting ability of the protein nanoassembly via flow cytometry (Fig. 3d). Previous studies collectively indicate that DCs present elevated expression of mannose receptors^37^, which could bind to mannose groups in a highly specific manner and thus lays the mechanistic basis for the DC-targeting ability of the mannose-modified BSA units. Interestingly, the total FITC fluorescence in BSA-FITC-Man@Mn^2+^-Ft@Lap-treated DCs under pH 6.5 was almost 6 times higher than that under pH 7.4, while no significant difference was observed in the uptake of BSA-FITC-Man@Mn^2+^-Ft@Lap and BSA-FITC@Mn^2+^-Ft@Lap by DCs under pH 7.4. In contrast, FITC accumulation in those BSA-FITC-Man-Ft@Lap-treated DCs under pH 6.5 dropped significantly by 63% after adding mannose receptor 1 antibody to block the mannose-dependent receptor-mediated endocytosis of the FITC-labeled BSA units (Supplementary Fig. 4c, d). These observations not only confirmed the DC-targeting ability of the mannose groups but also revealed that the Schiff-base ligation could temporarily shield the mannose groups to avoid non-specific accumulation. It is of interest to note that the inhibition effect of TfR1 antibody and mannose receptor 1 antibody on the uptake of the protein units was less significant under an environmental pH of 7.4, which could be explained that the majority of the targeting moieties in the cross-linked protein units were still in a caged state and therefore their uptake by DCs or tumor cells was less affected when disabling the specific ligand-receptor binding. Overall, the CLSM and flow cytometric results above collectively supported our hypothesis that the DC and tumor cell targeting ability of the BSA-Man and Ft components could be activated in an on-demand manner through the acidity-triggered dissociation of the cross-linked structure, which laid the mechanical basis for the strategic immunomodulation of the TME with the protein nanoassembly after systemic administration.

### Nanoassembly induced immunogenic apoptosis in tumor cells

Chemotherapy-mediated immunogenic cell death (ICD) is an emerging concept in immunotherapy, which could restore the immunogenicity of tumor cells for improving the immunotherapeutic outcome. Previous studies already demonstrated that Lap could be activated by abundant endogenous NAD(P)H:quinone oxidoreductase 1 (NQO1) in tumor cells to induce tumor cell apoptosis and cause the release of immunostimulatory damage-associated molecular patterns (DAMPs) including high mobility group protein 1 (HMGB1), ATP, membrane calreticulin (CRT), heat shock protein 70 (HSP70) and dsDNA^38–40^. The abnormally upregulated NQO1 expression in 4T1 tumor cells was also confirmed using western blot assay, which was 8-fold higher than the healthy HC11 mouse mammary epithelial cells, suggesting the intrinsic tumor specificity of the nanoassembly-mediated chemotherapy (Supplementary Fig. 5). We first monitored the apoptosis-inducing capability of the Lap-incorporated nanoassembly on 4T1 tumor cells. As shown in Supplementary Fig. 6, the viability of BSA-FITC-Man@Mn^2+^-Ft@Lap-treated 4T1 cells under pH 6.5 dropped below 60% under the equivalent Lap dose of 3.5 μg/mL according to MTT assay. Flow cytometric analysis with Annexin V-FITC/PI apoptosis assay kit consistently revealed that the apoptotic ratio of 4T1 tumor cells reached around 28% after the treatment with BSA-Man@Mn^2+^-Ft@Lap, which was also supported by the cell live/dead imaging results (Supplementary Fig. 7). Western blotting assay further showed that BSA-FITC-Man@Mn^2+^-Ft@Lap treatment induced significant upregulation of pro-apoptotic BAX proteins while downregulating anti-apoptotic Bcl-2 proteins (Fig. 3g). These results were in good agreement with the trends in the uptake evaluations and again validated the potential therapeutic benefit of the acidity-triggered nanoassembly dissociation for enhancing Lap delivery to tumor cells. It was also noteworthy that the treatment with non-drug-loaded BSA-Man-Ft nanoassemblies showed no apparent impact on tumor viability and apoptosis levels (Supplementary Fig. 7), suggesting their biocompatibility for drug delivery applications. The evidence above collectively verified the apoptosis-inducing ability of BSA-FITC-Man@Mn^2+^-Ft@Lap nanoassemblies in 4T1 tumor cells.

In view of the potent BSA-Man@Mn^2+^-Ft@Lap-mediated apoptosis in 4T1 tumor cells, we hypothesize that treating tumor cells with the Lap-incorporated protein nanoassembly would lead to the release of abundant DAMPs into the TME, which could be exploited for DC-mediated immune stimulation. Remarkably, western blot and immunofluorescence analysis revealed that CRT and HSP70 expression on 4T1 tumor cells increased by 1.6-fold and 1.5-fold after the incubation with BSA-Man@Mn^2+^-Ft@Lap compared to the control group (Fig. 3e–j and Supplementary Fig. 8), while ELISA assay of the supernatant consistently showed a 6- and 2.6-fold increase in HMGB1 and ATP release. The changes in CRT, HMGB1, HSP70, and ATP levels after BSA-FITC-Man@Mn^2+^-Ft@Lap treatment are immediate evidence for the ICD-inducing ability of the nanoassembly on 4T1 tumor cells, which would constitute a major source of tumor-derived dsDNA according to previous insights^41,42^. These observations supported our hypothesis that treating 4T1 tumor cells with BSA-FITC-Man@Mn^2+^-Ft@Lap could substantially enhance their immunogenicity through releasing abundant DAMPs, which may facilitate the subsequent activation of tumor-specific CD4 and CD8 T cells for enhancing the antitumor immune response.

### Nanoassembly augmented tumor-derived dsDNA sensing in DCs

**Fig. 4:**
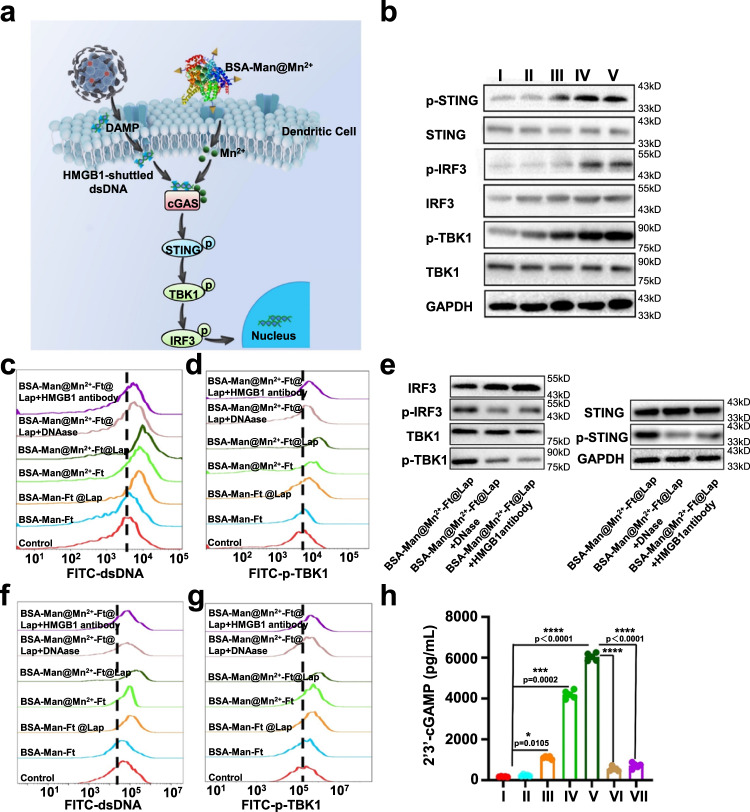
BSA-Man@Mn^2+^-Ft@Lap nanoassembly stimulates cGAS-STING-mediated dsDNA sensing machineries in DCs. **a** Schematical illustration of the nanoassembly-stimulated cGAS/STING signaling of DCs in the TME. **b** Western blot analysis of the expression levels of STING, p-STING, TBK1, p-TBK1, IRF-3, and p-IRF-3 in BMDCs after the conditioning with supernatants from 4T1 cells treated with (I) control, (II) BSA-Man-Ft, (III) BSA-Man-Ft@Lap, (IV) BSA-Man@Mn^2+^-Ft, and (V) BSA-Man@Mn^2+^-Ft@Lap, respectively. **c**, **d** Flow cytometric analysis of dsDNA uptake and p-TBK1 levels in BMDCs incubated with 4T1 cells after different treatments. **e** Western blot analysis of the expression level of STING, p-STING, IRF-3, p-IRF-3, TBK1, and p-TBK1 in BMDCs after treatment with supernatants from 4T1 cells treated with BSA-Man@Mn^2+^-Ft@Lap, BSA-Man@Mn^2+^-Ft@Lap+DNase, and BSA-Man@Mn^2+^-Ft@Lap+HMGB1 antibody, respectively. **f**, **g** Flow cytometric analysis of dsDNA uptake and p-TBK1 levels in splenic cells co-cultured with 4T1 cells after different treatments. **h** cGAMP production in BMDCs after treatment with (I) control, (II) BSA-Man-Ft, (III) BSA-Man-Ft@Lap, (IV) BSA-Man@Mn^2+^-Ft, (V) BSA-Man@Mn^2+^-Ft@Lap, (VI) BSA-Man@Mn^2+^-Ft@Lap+DNase, and (VI) BSA-Man@Mn^2+^-Ft@Lap+HMGB1 antibody, measured by ELISA. Flow cytometry and western blot experiments in panels **b**–**g** were repeated three times independently with similar results. Data are presented as mean values ± SEM (*n* = 5 biologically independent samples for panel **h**). Statistical analysis was carried out via two-way ANOVA method. * Indicates significance at *p* < 0.05, *** indicates significance at *p* < 0.001, **** indicates significance at *p* < 0.0001. Source data are provided as a Source data file.

The antigen-presenting function of DCs in solid tumor milieu is usually impaired due to the immunosuppressive nature of the TME, which imposes strong negative impact on vital immunological events including DC maturation, cross-priming and immune stimulation, eventually contributing to immune escape of the tumor cells^43^. To address this challenge, we strategically stimulated DC function via Mn^2+^ ions using a nanointegrated approach. According to the recent report on the immunostimulatory activity of HMGB1^44^, we hypothesized that HMGB1 could bind to the concurrently released tumor-derived dsDNA and shuttle them to DCs to activate the cGAS-STING pathway therein after the nanoassembly-induced ICD of tumor cells. Meanwhile, Mn^2+^ ions would complex with cGAS and STING in DCs to enhance their signaling sensitivity to dsDNA, eventually leading to strong antitumor immune response via robust DC activation (Fig. 4a). To test our hypothesis, we extracted the supernatant of nanoassembly-treated 4T1 tumor cells (incubated at pH 6.5 for 24 h) post low-speed centrifugation and used it for murine bone marrow-derived dendritic cell (BMDC) conditioning, while dsDNA antibody was subsequently added for monitoring the dsDNA sensitivity of BMDCs (Fig. 4c). Due to the elevated TfR1 expression on 4T1 tumor cells, the majority (80%) of the BSA units remained in the supernatant, while the mount of residual Lap-laden Ft units were almost negligible (<10%) (Supplementary Fig. 9). It was observed that the dsDNA uptake and cGAMP abundance in the BSA-Man-Ft group remained at baseline level after 24 h of incubation, excluding the potential contribution of the protein-based carrier to DC stimulation. In contrast, the dsDNA uptake in the BSA-Man-Ft@Lap and BSA-Man@Mn^2+^-Ft@Lap-treated BMDCs increased substantially by 2.5- and 5-fold, accompanied with a 6- and 34-fold increase in cGAMP levels. These nanoagonist-induced changes not only supported the successful induction of DAMP release via Lap medication but also confirmed cGAS-STING stimulation function of the cooperatively delivered Mn^2+^ (Fig. 4c, f, h). Moreover, adding anti-HMGB1 antibodies or dsDNA-degrading DNase both caused a sharp decrease in dsDNA uptake and cGAMP production in DCs treated by BSA-Man-Ft@Lap or BSA-Man@Mn^2+^-Ft@Lap, mechanistically validating that the dsDNA uptake by DCs was HMGB1 dependent. To determine if the nanoagonist-enabled DC activation effect involves BMDC-derived dsDNA, we monitored the changes in BMDC viability after incubation with different nanoagnoists under pH 6.5 and found that BMDCs were evidently more resistant to Lap-induced ROS production and apoptosis than tumor cells, which could be explained by the antitumor mechanism of Lap as well as its low efficient uptake by BMDCs (Supplementary Fig. 9). It is well understood that the reduction of Lap to its cytotoxic hydroquinone form is mediated by the endogenous NQO1 enzyme, of which the expression levels are abnormally upregulated in tumor cells but remain normal in immune cells. Meanwhile, tumor cells are the largest cell population in the TME and have much higher uptake rate for Ft-Lap, thus further attenuating the cytotoxic impact on the immune cells therein. The low death rate of BMDCs in response to Lap treatment suggests that the most of the dsDNAs in the post-treatment TME are originated from tumor cells and supports the tumor-specificity of the nanoagonist-stimulated cGAS-STING-mediated immunity. Similar to the assay results on BMDCs, we observed that BSA-Man@Mn^2+^-Ft@Lap nanoassembly also pronouncedly enhanced the dsDNA sensing capability of ex vivo harvested splenic immune cells, which are another commonly used cellular model in immunological research. Alternative to DC conditioning with tumor cell debris, we also co-incubated BMDCs with 4T1 tumor cells under pH 6.5 to mimic the two cell populations in the TME and then added different samples. Western blot data showed that the expression levels of major components in cGAS-STING signaling including phosphorylated STING (p-STING), phosphorylated IRF3 (p-IRF3), and phosphorylated TBK1 (p-TBK1) have been significantly upregulated in BSA-Man@Mn^2+^-Ft@Lap-treated BMDCs in response to tumor-derived dsDNA, while the protein levels of non-phosphorylated STING, IRF3, and TBK1 remained unchanged. Notably, no significant changes were observed in the expression levels of p-STING, pTBK1, and p-IRF3 when BSA-Man@Mn^2+^-Ft@Lap-treated DCs were co-incubated with antiHMGB1 antibody or DNase, substantiating the central role of HMGB1-mediated dsDNA shuttling for cGAS-STING activation in DCs (Fig. 4b, d, e, g). The comparative analysis of DC activity after conditioning with tumor cell debris or co-incubation with tumor cells also confirmed that the cGAS-STING activation in DCs was caused by the tumor-derived dsDNA rather than those membrane-bound antigens.

**Fig. 5:**
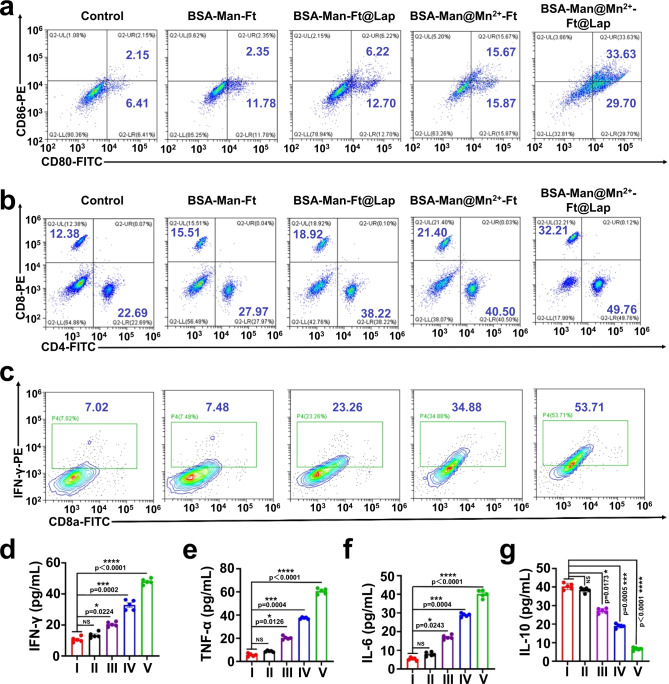
BSA-Man@Mn^2+^-Ft@Lap nanoassembly promotes DC maturation and cross-priming. **a**–**c** Flow cytometric analysis of the expression levels of mature DC markers CD80/CD86 and effector T cell markers CD4/CD8 and CD8a/ IFN-γ in splenic immune cells co-incubated with 4T1 cells treated with: (I) control, (II) BSA-Man-Ft, (III) BSA-Man-Ft@Lap, (IV) BSA-Man@Mn^2+^-Ft, or (V) BSA-Man@Mn^2+^-Ft@Lap for 24 h; flow cytometry experiments in panels **a**–**c** were repeated three times independently with similar results. **d**–**g** Secretion levels of immunostimulatory cytokines including IFN-γ, TNF-α, IL-6, and IL-10 in the supernatant from the above co-culture system with different treatments, including (I) control, (II) BSA-Man-Ft, (III) BSA-Man-Ft@Lap, (IV) BSA-Man@Mn^2+^-Ft and (V) BSA-Man@Mn^2+^-Ft@Lap. Data are presented as mean values ± SEM (*n* = 5 biologically independent samples for panels **d**–**g**). Statistical analysis in panels **d**–**g** was carried out via one-way ANOVA method. * Indicates significance at *p* < 0.05, *** indicates significance at *p* < 0.001, ****indicates significance at *p* < 0.0001. Source data are provided as a Source data file.

To test our hypothesis that the nanoassembly-induced DC activation enhances tumor-specific T cell immunity, we constructed a co-culture system composed of 4T1 tumor cells and ex vivo harvested splenic immune cells for recapitulating key immunological features in the TME. Importantly, flow cytometric results revealed that BSA-Man@Mn^2+^-Ft@Lap treatment caused significant upregulation of CD80/CD86 co-stimulatory ligands on DC surface (Fig. 5a), which was 55% higher than the control group and evidently confirming the enhanced DC maturation due to cooperative DAMP release and Mn^2+^ stimulation. As a result of the boosted DC functions in the co-incubation system, the amount of CD4/CD8 T cells have increased by 47% compared to the control group (Fig. 5b, c), accompanied with elevated cellular IFN-γ levels (46%). We further employed ELISA assay to investigate the cytokines secretion and found that the secretion level of pro-inflammatory cytokines interleukin-6 (IL-6), tumor necrosis factor-α (TNF-α), and IFN-γ increased by 8-, 12-, and 6-fold compared to the control group, while the anti-inflammatory cytokine interleukin-10 (IL-10) decreased by 80% (Fig. 5d–f and Supplementary Fig. 10). Due to the nanoassembly-enhanced generation and activation of tumor-specific effector T cells, the apoptosis ratio of the 4T1 tumor cells in the 4T1/splenic immune cell co-culture system increased substantially from 2.8% in the control group to 80.4% in the BSA-FITC-Man@Mn^2+^-Ft@Lap group (Supplementary Fig. 11), supporting the efficient clearance of the poorly immunogenic 4T1 cells thereof.

Nevertheless, it is acknowledged that the TME is a complex ecosystem and the Lap-induced ICD of tumor cells may also activate TLR4 signaling in DCs to stimulate antitumor immune responses^45^. To determine the potential therapeutic contribution of cGAS-STING signaling pathway in the nanoagonist-mediated antitumor effects, we have comprehensively studied the responses of splenic immune cells to tumor-derived DAMPs through comparative analysis. Notably, even the Mn-free nanoassembly could elicit moderate immunostimulatory effects on immune cells, as we observed that the supernatant from BSA-Man-Ft@Lap-treated tumor cells caused a modest (5.52%) increase in DC maturation ratio (CD86/CD80) and slightly expanded CD8a+/IFN-γ+ and CD4/CD8 T cell populations (8 and 20%) compared to the control group, suggesting that the Lap-induced ICD of tumor cells could activate T cell-mediated immune response even without proper cGAS-STING stimulation (Supplementary Figs. 12–13). However, the addition of TLR4 antibodies caused an evident decrease in DC maturation and T cell activation across all groups, confirming that the DAMP-activated TLR4 signaling pathway may also contribute to the nanoagonist-mediated immunostimulatory effects. In contrast to the apparent susceptibility of the BSA-Man-Ft@Lap-mediated immunotherapeutic effect to TLR4 inhibition, the BSA-Man@Mn^2+^-Ft@Lap still demonstrated pronounced antitumor immune responses despite TLR4 inhibition, of which the DC maturation ratio and T cell activation effects were significantly superior to BSA-Man-Ft@Lap with or without TLR4 inhibition. These results not only revealed the contribution of conventional TLR4-based immunotherapeutic activities in the present study but also highlighted its potent antitumor effect through cooperative cGAS-STING activation, substantiating the immunostimulatory capability of the BSA-Man@Mn^2+^-Ft@Lap nanoagonist to induce robust tumor-specific T cell immunity in vitro.

### Biodistribution of the protein nanoassembly

**Fig. 6:**
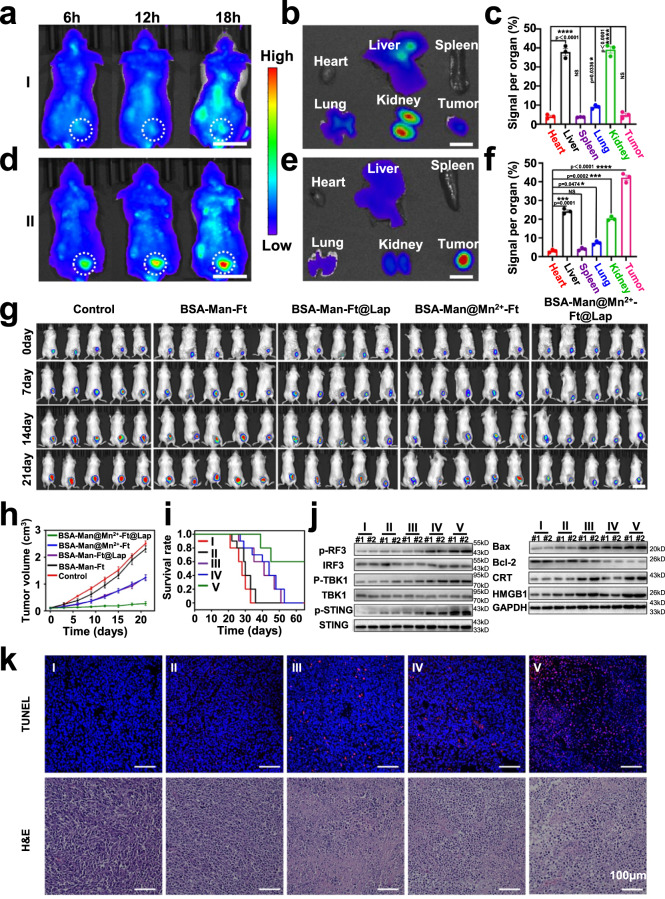
BSA-Man@Mn^2+^-Ft@Lap nanoassembly was selectively activated in solid tumors to induce potent immune response for tumor suppression. **a**, **b**, **d**, **e** Panels **a**, **d** are the systemic distribution of the samples at 6/12/18 h post intravenous injection. I: Non-dissociable amide-ligated BSA-Man@Mn^2+^-Ft@Lap@Cy5; II: Tumor-responsive Schiff base-ligated BSA-Man@Mn^2+^-Ft@Lap@Cy5. Scale bar = 2 cm; panels **b**, **e** show the fluorescence distribution in major organs and tumors after the euthanasia. Scale bar=1 cm; panels **c**, **f** are the corresponding quantitative analysis results in each group. **g** In vivo bioluminescence analysis of 4T1-Luc tumor-bearing mice after different treatments. (I) control, (II) BSA-Man-Ft, (III) BSA-Man-Ft@Lap, (IV) BSA-Man@Mn^2+^-Ft, and (V) BSA-Man@Mn^2+^-Ft@Lap. Scale bar = 3 cm. **h** Tumor size changes during the incubation period after different treatment. **i** Survival analysis of mice after treatment. **j** Western blot analysis on the expression of damage-associated molecular patterns (HMGB1 and CRT), apoptosis markers (BAX, Bcl-2), and key cGAS/STING signaling pathway makers (STING, p-STING, IRF-3, p-IRF-3, TBK1, and p-TBK1) in tumors from 4T1 tumor-bearing mice after treatment with different samples. **k** H&E staining and TUNEL staining results of tumor tissue samples after different treatment. (I) control, (II) BSA-Man-Ft, (III) BSA-Man-Ft@Lap, (IV) BSA-Man@Mn^2+^-Ft, and (V) BSA-Man@Mn^2+^-Ft@Lap. In vivo western blot and histological experiments in panels **j** and **k** were repeated three times independently with similar results. Data are presented as mean values ± SEM (*n* = 3 mice for panels **c** and **f**, *n* = 5 mice for panel **h**). Statistical analysis for panels **c** and **f** was carried out via one-way ANOVA method. * Indicates significance at *p* < 0.01, *** indicates significance at *p* < 0.001, ****indicates significance at *p* < 0.0001. Source data are provided as a Source data file.

To determine whether the protein nanoassemblies could be used for the immunomodulation of solid tumors in vivo, we first monitored their transportation, accumulation, and activation in immunocompetent mice after systemic administration. Pharmacokinetic analysis showed that the Cy5-labeled BSA-Man@Mn^2+^-Ft@Lap nanoassembly has a blood half-life of around 8 h (Supplementary Fig. 15c), while pristine Cy5 molecules were rapidly cleared from the bloodstream within 2 h. The long blood circulation time of the Cy5-labeled BSA-Man@Mn^2+^-Ft@Lap nanoassembly was ascribed to the high biostability and low immunogenicity of the protein components and was beneficial for drug delivery via blood route. We also prepared TME-dissociable and non-dissociable nanoassemblies employing acid-triggerable Schiff base or non-responsive amide linkers to investigate the tumor-specific drug delivery capacity of the nanoagonist. Fluorescence tracking showed only modest accumulation of amide-ligated nanoassemblies in the tumor region (Fig. 6a), which most accumulated in organs with rich mononuclear phagocyte system including liver, lungs and kidneys (Fig. 6b). In comparison, Schiff base-ligated Cy5-labeled BSA-Man@Mn^2+^-Ft@Lap nanoassemblies primarily accumulated in the tumor tissues in a time-dependent manner (Fig. 6d), while fluorescence deposition in those non-specific organs and tissues remained at a visibly low level (Fig. 6e). The visual trends of fluorescence distribution were further confirmed via quantitative analysis (Fig. 6c, f), for which the relative tumor accumulation ratio of Schiff base-ligated nanoassemblies reached around 43% while that for the amide-ligated ones was only around 5%. The low tumor accumulation of the amide-ligated nanoassemblies is due to the complex biological barriers in solid tumors, which severely impede the accumulation and penetration of the protein-based nanoassembly. In contrast, the Schiff base-ligated BSA-Man@Mn^2+^-Ft@Lap nanoassemblies could readily disintegrate into highly diffusive BSA and Ft units, which may circumvent those solid tumor-associated barrier effects and improve the retention and penetration of the disconnected protein units into the tumor stroma, thus facilitating their therapeutic interaction with the designated cellular targets in the TME^46,47^. Meanwhile, this would also reduce the non-specific uptake of the nanoagonist and thus avoid overstimulation of the adaptive immune system at a systemic level. To further elucidate the nano-biointeraction patterns of the nanoagonist in vivo, we have proactively labeled the BSA and Ft protein units separately with FITC and Rhodamine B (RhB) fluorescent probes and constructed TME-responsive Schiff-base-ligated and non-responsive amide-ligated nanoassemblies for fluorescence analysis. For the amide-ligated BSA-Man@Mn^2+^-FITC -Ft@Lap@RhB nanosamples, we observed that only sparse red and green fluorescence appeared in the tumor samples, suggesting the inefficient accumulation of the nanocomplex in the tumor tissues after systemic administration. Moreover, the red and green fluorescence largely overlapped due to the inability to dissociate (Supplementary Fig. 14a). In contrast, the deposition of red and green fluorescence in the Schiff base-ligated BSA-Man@Mn^2+^-FITC -Ft@Lap@RhB group was evidently higher and their distribution was well separated, for which the red fluorescence was predominantly detected in 4T1 tumor cells. The distinct fluorescence patterns supported our hypothesis that the TME-dependent disintegration of the protein-based complex could yield superior tumor-targeted drug delivery efficacy in clinically relevant conditions. Extending from the fluorescent imaging results above, we further extracted the tumor tissues for FACS-based quantitative fluorescence analysis to determine the actual delivery efficacy of the protein-based nanocomplex to individual cell populations in the TME. Specifically, the DCs were analyzed using CD11c antibodies, while the tumor cells were gated as antiCD45- population. According to the flow cytometric analysis on the sorted cell populations, we observed that the FITC fluorescence levels in DCs increased by 8-fold, accompanied with a 7.6-fold increase in RhB fluorescence for tumor cells (Supplementary Fig. 14b, c). In comparison, only modest changes were observed in the fluorescence accumulation in DCs and tumor cells in the amide-ligated BSA-Man@Mn^2+^-FITC-Ft@Lap@RhB group. The fluorescence imaging and flow cytometric results supported that the acidity-triggerable dissociation of the protein-based nanoassembly could help to overcome the intrinsic drug delivery barriers in solid tumors to realize the proposed therapeutic functions.

On the other hand, we have also comprehensively investigated the toxicity of the nanoagonist on 4T1 tumor-bearing Balb-c mouse model after systemic administration. Histological analysis on H&E-stained major organs revealed no apparent tissue damage or inflammatory features (Supplementary Fig. 15e), suggesting that the nanoagonist did not cause any acute toxicity. According to the in vivo distribution profiles, liver and kidney are the primary sites of non-specific drug accumulation after systemic administration of the nanoagonist due to the richly deposited mononuclear phagocytic system, which accounted for around 40% of the total drug deposition in vivo. We thus monitored key liver indices (ALT and AST) and kidney indices (urea and creatinine) after different treatment and found that all these parameters were within normal range with no significant changes, indicating that the adverse impact of the nanoagonist on liver/kidney function was negligible. Furthermore, it was found that the nanoagonist didn’t induce hemolysis of erythrocytes even under a high concentration of 150 μg/mL (Supplementary Fig. 16), nor cause significant changes in key blood indices including RBC, WBC, PLT, and HGB after intravenous injection, which not only validated its applicability for systemic administration but also again affirmed that it didn’t induce overstimulation of the adaptive immune system at a systemic level (Supplementary Fig. 17).

### Nanoassembly-mediated immunostimulation prolongs mouse survival, inhibits distal tumor growth, and reduces lung metastasis

To investigate the therapeutic efficacy of the cooperative cGAS-STING nanoagonist in vivo, we constructed immunocompetent 4T1-luc tumor-bearing mice, which is a well-established animal tumor model with poor immunogenicity and low response rate to existing immunotherapeutic modalities^48^. Consistent with the immunoresistance of 4T1 tumors in previous reports^49,50^, mice treated by BSA-Man-Ft showed uncontrolled and rapid growth similar to the PBS group, of which the tumor size grew to around 2.5 cm^3^ after 21 days of incubation and suggested the non-toxicity of the protein-based carrier itself (Fig. 6g, h and Supplementary Fig. 15a). Meanwhile, BSA-Man-Ft@Lap and BSA-Man@Mn^2+^-Ft both showed modest inhibition effect against 4T1 tumors with an average final tumor size of around 1.34 and 1.28 cm^3^, respectively, which could be explained by the limited antitumor efficacy of Lap-based chemotherapy and Mn^2+^-mediated DC activation when administered separately. Remarkably, mice in the BSA-Man@Mn^2+^-Ft@Lap group showed the most pronounced tumor inhibition effect with an average final tumor size of around 0.27 cm^3^. Similar trends were also observed for the tumor weight and survival analysis. Specifically, the BSA-Man@Mn^2+^-Ft@Lap group presented an average final tumor weight of around 0.3 g and a median survival time of 60 days (Fig. 6i and Supplementary Fig. 15d), which was evidently superior to all the other groups. Western blot analysis on the tumor tissue samples further showed that BSA-Man@Mn^2+^-Ft@Lap significantly upregulated the expression levels of BAX and Caspase-3 in tumors while downregulating Bcl-2 expression (Fig. 6j), which was in good accordance with the apoptosis-inducing capability of Lap and effector T cells against tumor cells. The tumor tissues in the mouse models were also extracted for histological analysis via hematoxylin and eosin (H&E) and terminal deoxynucleotidyl transferase dUTP nick end labeling assay (TUNEL) staining (Fig. 6k), and the results revealed that BSA-Man@Mn^2+^-Ft@Lap treatment induced the largest population of dead tumor cells among all groups, which was consistent with its potent apoptosis-inducing capability thereof. To test the general applicability of the nanoagonist in a clinical context, we also monitored its effects on B16F10-luc tumor-bearing C57 mice, which is another commonly used tumor model for preclinical evaluation. Consistent with the observations on 4T1-luc tumors, mice in the BSA-Man@Mn^2+^-Ft@Lap group showed the most pronounced tumor inhibition effect, evidenced by the smallest final tumor size (0.3 cm^3^), lowest tumor weight (0.27 g), longest median post-treatment tumor survival time (60 days) as well as the greatest tumor cell apoptosis according to H&E and TUNEL staining (Supplementary Figs. 18 and 19). Moreover, histological features of major organs of the B16F10-luc tumor-bearing C57 mice all remained normal after the nanoagonist-mediated immunotherapy, against validating its safety for in vivo applications. The nanoagonist-enabled efficient inhibition of both 4T1 and B16F10 tumors immediately suggests its potential application against a variety of solid tumor indications.

**Fig. 7:**
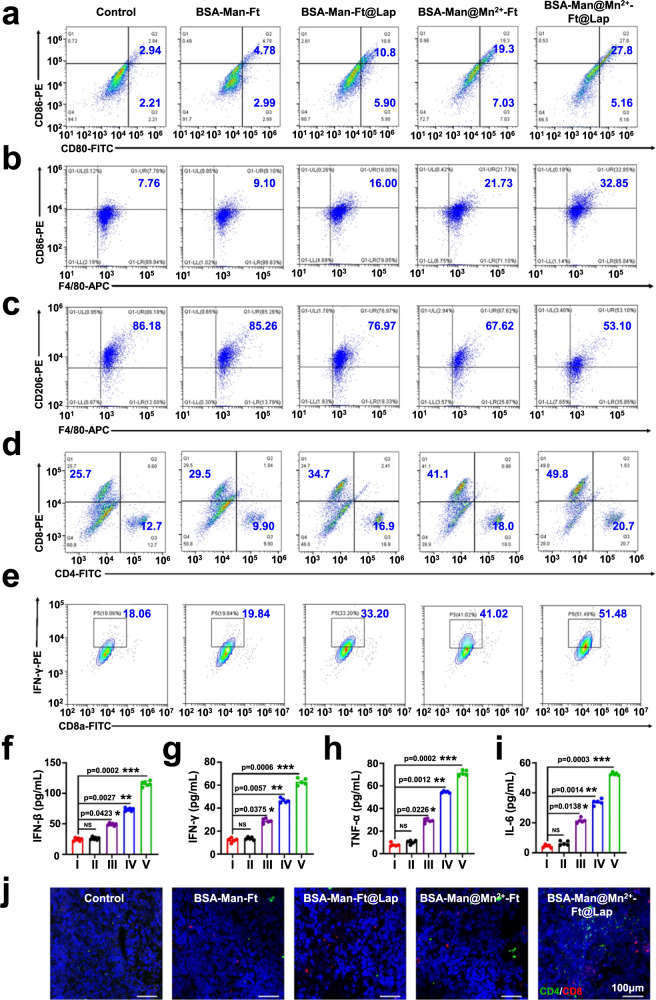
BSA-Man@Mn^2+^-Ft@Lap nanoassembly promotes DC maturation and cross-priming in vivo to enhance T cell infiltration and effector function. **a**–**e** Flow cytometric analysis on the expression levels of DC maturation (CD80/CD86), M1/M2 macrophage ratio (F4/80/CD86^+^ and F4/80/CD206^+^), and T cell activation state (CD4/CD8, CD8a/IFN-γ) in 4T1 tumor tissues after treatment with (I) control, (II) BSA-Man-Ft, (III) BSA-Man-Ft@Lap, (IV) BSA-Man@Mn^2+^-Ft, and (V) BSA-Man@Mn^2+^-Ft@Lap in vivo. **f**–**i** Serum levels of IFN-β, IFN-γ, TNF-α, and IL-6 in 4T1 tumor-bearing mice after different treatments. **j** Immunofluorescence analysis of the intratumoral infiltration of CD4/CD8 T cells in 4T1 tumor-bearing mice after different treatments. Flow cytometry and immunofluorescence experiments in panels **a**–**e** and **j** were repeated three times independently with similar results. Data are presented as mean values ± SEM (*n* = 6 mice for panels **f**–**i**). Statistical analysis for panels **f**–**i** was carried out via one-way ANOVA method. * Indicates significance at *p* < 0.05, ** indicates significance at *p* < 0.01, *** indicates significance at *p* < 0.001. Source data are provided as a Source data file.

To elucidate the antitumor mechanism of the BSA-Man@Mn^2+^-Ft@Lap nanoassembly, we subsequently measured the abundance of DAMPs in the tumor tissues as well as the immune cell responses in tumors and spleens. WB and immunofluorescence assay on the extracted 4T1-luc tumor tissue samples showed evidently increasing levels of typical DAMPs including HMGB1 and CRT, evidencing the successful ICD induction in vivo (Fig. 6j and Supplementary Fig. 20). Meanwhile, treating 4T1-luc tumor-bearing mice with BSA-Man@Mn^2+^-Ft@Lap induced a drastic increase in the amount of tumor-resident mature DCs (Fig. 7a) as well as the expression of p-STING, p-IRF3, and p-TBK1 therein, while the expression level of STING/IRF3/TBK1 remained unchanged similar to the in vitro observations (Fig. 6j). Specifically, the maturity ratio of tumor-infiltrating DCs reached around 33% compared to the BSA-Man-Ft@Lap (17%) and BSA-Man@Mn^2+^-Ft groups (26%), suggesting the potent DC stimulating effect of the BSA-Man@Mn^2+^-Ft@Lap nanoassembly through cooperative tumor-derived dsDNA release and DC-targeted cGAS-STING stimulation. Meanwhile, the maturity ratio of DCs in the spleen of 4T1-luc tumor-bearing mice also increased from 7% in the control group to 43% in the BSA-Man@Mn^2+^-Ft@Lap group (Supplementary Fig. 21), which was ascribed to the homing of stimulated DCs as well as the pro-maturation effect of tumor-derived DAMPs captured from peripheral blood and beneficial for eliciting systemic antitumor immunity^51,52^. It is well-established that mature DCs express high levels of both MHC-I and MHC-II molecules, which are responsible for presenting the antigenic peptides to prime naïve CD8 and CD4 T cells, respectively. Consistent with the mechanistic insights in previous reports^53,54^, flow cytometric analysis on the DC populations in the tumors of the BSA-Man@Mn^2+^-Ft@Lap group showed that their MHC-I and MHC-II expression levels have increased by 36.18% and 31.68% compared to the control group, supporting the enhanced capacity of these DCs to activate tumor-specific CD8 and CD4 T cells (Supplementary Fig. 22). Moreover, we detected that treating 4T1-luc tumor-bearing mice with BSA-Man@Mn^2+^-Ft@Lap has also reprogrammed the anti-inflammatory M2 macrophages to the pro-inflammatory M1 phenotype and significantly altered the M1/M2 macrophage ratio in the TME from 0.09:1 to 0.62:1. This phenomenon could be explained by the intrinsically high mannose receptor (CD206) expression levels in M2 macrophages and the resultant cGAS-STING stimulation (Fig. 7b, c)^55^. Considering the immunostimulatory functions of M1 macrophages in various immunotherapeutic designs, the nanoagonist-mediated M2-to-M1 macrophage repolarization may alleviate the immunosuppression in the TME and enhance the eventual T cell-mediated antitumor efficacy.

Extending from the nanoagonist-enhanced T cell priming capacity of DCs, we further measured the activated T cell populations (CD4/CD8 T cells) in tumor tissues and spleens after different treatment via immunofluorescence imaging and flow cytometry (Fig. 7d, e, j and Supplementary Figs. 21, 23, 24). The results showed that the tumor T cell infiltration in the BSA-Man@Mn^2+^-Ft@Lap group was the greatest, of which the activated T cell population was 32% larger than the PBS group. Consistently, mice in the BSA-Man@Mn^2+^-Ft@Lap group also showed the highest splenic activated T cell population, which was 37.4% higher than the PBS group and suggested that the immunomodulation strategy in this study potentiated efficient differentiation of naïve T cells into tumor-specific activated T cells. Similarly, we also observed that the immune cell activation and infiltration in B16F10-tumor-bearing C57 mice has been substantially enhanced after the treatment with BSA-Man@Mn^2+^-Ft@Lap, where the nanoagonist induced a significant increase in DC maturation (27%), M1/M2 macrophage ratio (0.52) and tumor-infiltrating CD4/CD8 T cell populations (9.72% and 21.48%). Notably, the BSA-Man@Mn^2+^-Ft@Lap treatment not only enhanced the tumor infiltration of immune cells but also boosted their immune functions. It is observed that the ISG56 expression in tumors have substantially increased by 4- and 4-fold for the 4T1 and B16F10 tumors after BSA-Man@Mn^2+^-Ft@Lap treatment, respectively. Additionally, serum levels of immunostimulatory cytokines including IFN-β, IL-6, TNF-α and IFN-γ have increased by varying degrees in 4T1 tumor (4-, 6.7-, 6-, and 4-fold) and B16F10 tumor (5-, 6.5-, 4-, and 3.2-fold) bearing mice (Fig. 7f–i and Supplementary Figs. 25 and 26), validating the successful induction of antitumor immune responses. Moreover, treating tumors with blank BSA-Man-Ft nanoassembly induced no significant changes in serum cytokine levels, confirming that the protein-based nanostructure itself is non-immunogenic. The observations above demonstrated that the BSA-Man@Mn^2+^-Ft@Lap nanoassembly is capable of promoting cGAS-STING-mediated DC maturation and activation and profoundly reshaping the immunosuppressive TME into an immunosupportive state. Antibody-mediated T cell depletion tests on the BSA-Man@Mn^2+^-Ft@Lap-treated 4T1-tumor-bearing Balb/c mice showed that the nanoagonist-induced CD8^+^ T cells are a major contributing factor for the treatment-induced tumor regression, for which the antitumor effects of the nanoagonist was largely abrogated in the antiCD4 and antiCD8 groups without inducing significant alterations in the DC maturation and M1 macrophage polarization levels. Meanwhile, depleting CD4^+^ T cells caused a modest reduction in the antitumor potency of the nanoagonist, which agrees with the supporting role of CD4 T cells to boost CD8^+^ T cell-mediated antitumor response and is consistent with the observations in previous reports^56,57^. It is also noteworthy that the nanoagonist still demonstrated significant tumor inhibitory effect under the combined treatment of antiCD4 and antiCD8, attributing to the cytotoxic activity of Lap (Supplementary Fig. 27). It could thus be concluded that the BSA-Man@Mn^2+^-Ft@Lap-mediated antitumor effect predominantly depends on the generated tumor-specific CD4 and CD8 T cell subsets.

**Fig. 8:**
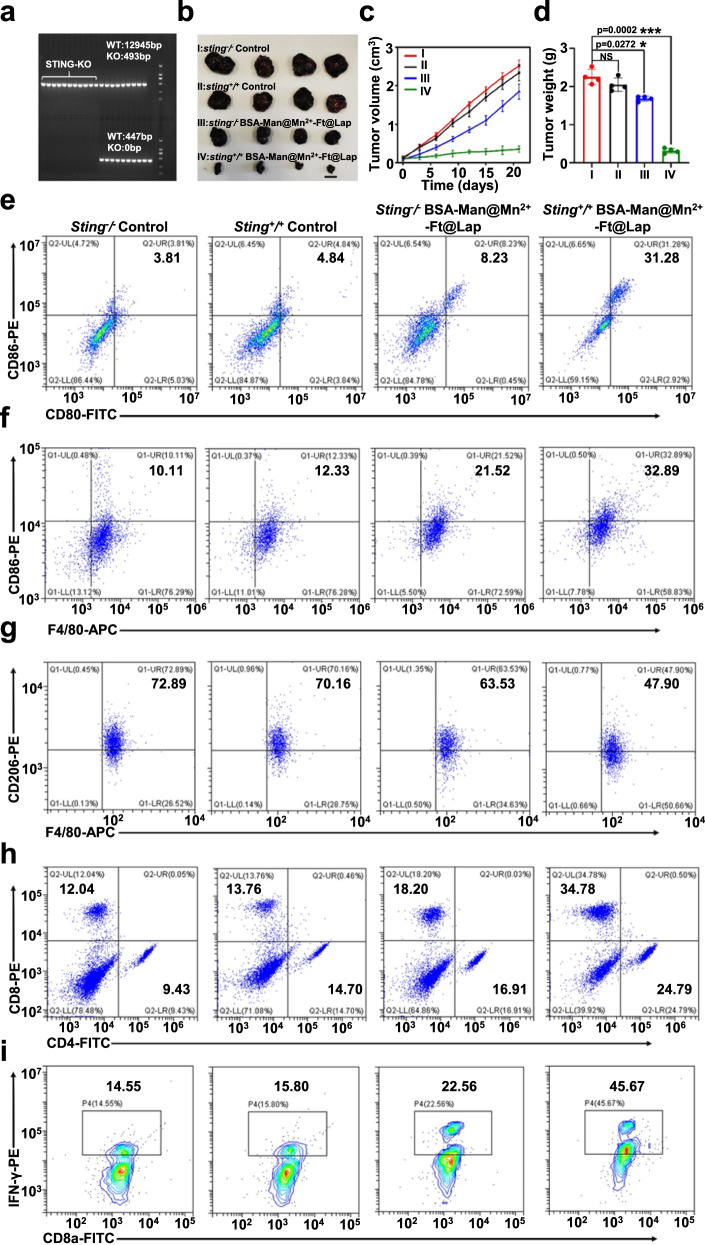
Immunostimulatory effects of BSA-Man@Mn^2+^-Ft@Lap nanoagonist on STING-KO B16F10 tumor-bearing C57 mice in vivo. **a** PCR assay of the STING-KO mice (*n* = 11 mice). **b** Visual comparison of the nanoagonist-induced antitumor effects on STING-KO B16F10 tumor-bearing C57 mice. Scale bar = 1 cm. **c** Change in tumor volumes on STING-KO B16F10 tumor-bearing C57 mice throughout the treatment period. **d** Weight comparison of B16F10 tumors on STING-KO mice after different treatments. **e**–**i** Flow cytometric analysis on the DC maturation (CD80/CD86), M1/M2 macrophage ratio (F4/80/CD80/CD206) and T cell activation status (CD4/CD8, CD8a/IFN-γ) in B16F10 tumors on STING-KO mice after different treatments. Flow cytometry experiments in panels **e**–**i** were repeated three times independently with similar results. Data are presented as mean values ± SEM (*n* = 4 mice for panels **c**, **d**). Statistical analysis for panel **d** was carried out via one-way ANOVA method. * indicates significance at *p* < 0.05, *** indicates significance at *p* < 0.001. Source data are provided as a Source data file.

To study the role of cGAS-STING signaling pathway in the nanoassembly-mediated immunotherapeutic effect, we established B16F10-tumor-bearing STING-knockout (STING-KO) C57 mouse models and comparatively analyzed their response to BSA-Man@Mn^2+^-Ft@Lap treatment. From an overall perspective, the tumor inhibition efficacy of BSA-Man@Mn^2+^-Ft@Lap nanoagonist on STING-KO mice was significantly lower than that on the wild-type group (WT), evidenced by the more rapid tumor growth in terms of volume and weight (Fig. 8a–d). Flow cytometric analysis on the FACS-sorted DC and macrophage populations further revealed a substantial decrease in DC maturation and M1/M2 macrophage ratio (23.05% and 0.63) in STING-KO mice compared to BSA-Man@Mn^2+^-Ft@Lap-treated WT mice, suggesting that the cGAS-STING signaling is necessary for the nanoagonist-enhanced DC activation and M2-to-M1 macrophage repolarization and consistent with the insights in previous reports^58^. Interestingly, we also observed that the mature DC and M1 macrophage populations in the tumors of BSA-Man@Mn^2+^-Ft@Lap-treated STING-KO mice was slightly higher than that in PBS-treated ones, which was attributed to the activation of alternative non-STING-dependent DAMP-sensing machineries such as TLR4. In accordance with the severely impaired DC function in STING-KO mice, we found that the BSA-Man@Mn^2+^-Ft@Lap-treated STING-KO mice failed to generate CD4 and CD8 T cells, thus abrogating its antitumor immunity (Fig. 8e–i). Overall, the analysis results on STING-KO mice collectively supported the crucial role of host cGAS-STING machineries in the nanoagonist-enabled tumor therapy.

**Fig. 9:**
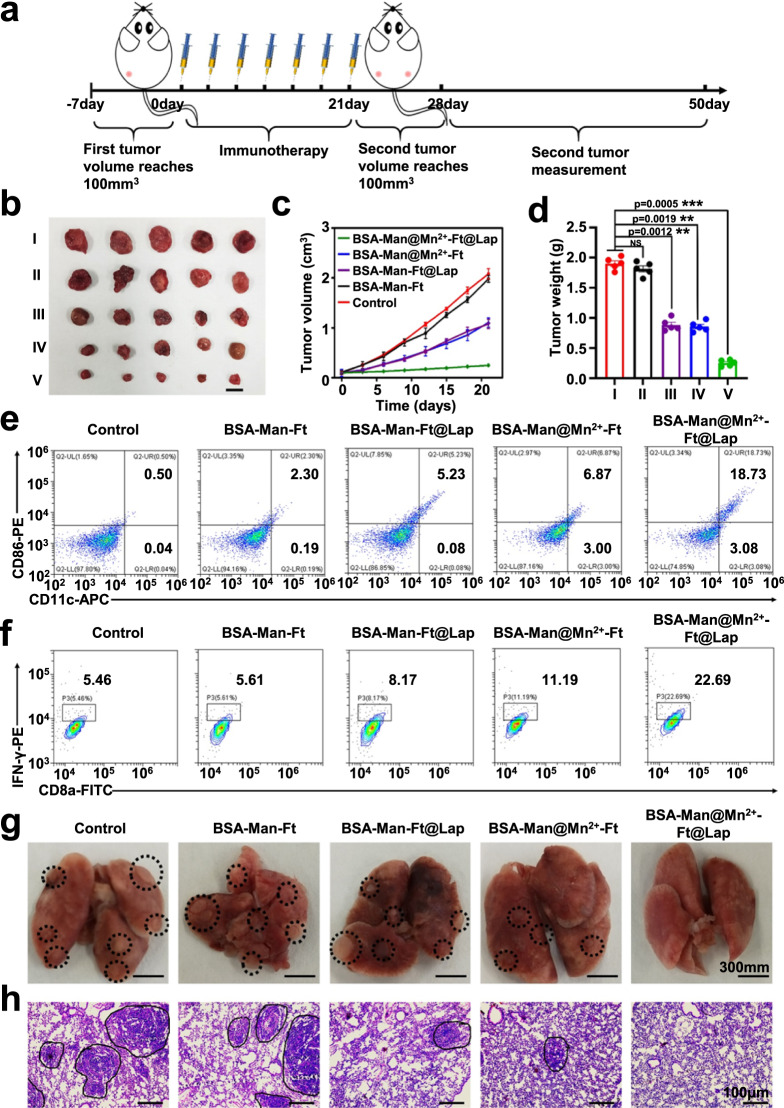
BSA-Man@Mn^2+^-Ft@Lap nanoassembly elicits systemic immunity to suppress distal tumors and lung metastasis. **a** Schematic illustration of the treatment procedures for bilateral tumors. **b** Images of the extracted distal tumors after various treatments including (I) control, (II) BSA-Man-Ft, (III) BSA-Man-Ft@Lap, (IV) BSA-Man@Mn^2+^-Ft, and (V) BSA-Man@Mn^2+^-Ft@Lap. Scale bar = 1 cm. **c** The change in the volumes of the distal tumors throughout the treatment period. **(d)** The average weight of distal tumors after different treatments. **e** Expression levels of CD80/CD86 in DCs from lymph nodes of the bilateral 4T1-tumor models after treatment with (I) control, (II) BSA-Man-Ft, (III) BSA-Man-Ft@Lap, (IV) BSA-Man@Mn^2+^-Ft, and (V) BSA-Man@Mn^2+^-Ft@Lap. **f** Expression levels of CD8a/IFN-γ markers of T cells in distal tumors after treatment with (I) control, (II) BSA-Man-Ft, (III) BSA-Man-Ft@Lap, (IV) BSA-Man@Mn^2+^-Ft, and (V) BSA-Man@Mn^2+^-Ft@Lap. **g** Photographs regarding lung metastasis in 4T1-tumor-bearing mice after different treatment (I) control, (II) BSA-Man-Ft, (III) BSA-Man-Ft@Lap, (IV) BSA-Man@Mn^2+^-Ft, and (V) BSA-Man@Mn^2+^-Ft@Lap. **h** The H&E staining of lungs after different treatments. Black circles indicate lung metastasis modules. Experiments in panels **e**–**h** were repeated three times independently with similar results. Data are presented as mean values ± SEM (*n* = 5 mice for panels **c**, **d**). Statistical analysis for panel **d** was carried out via one-way ANOVA method. ** indicates significance at *p* < 0.01, *** indicates significance at *p* < 0.001. Source data are provided as a Source data file.

To further determine whether the nanoassembly-induced antitumor T cell immunity could protect the mice from distal tumors, we have treated 4T1 tumor-bearing mice with different samples and subsequently inoculated a secondary 4T1 tumor on the opposite flank after 21 days. Remarkably, the growth of distal tumors in the BSA-Man@Mn^2+^-Ft@Lap group has been substantially inhibited (Fig. 9a–d and Supplementary Fig. 28a), of which the size (<0.2 cm^3^) and weight (0.2 g) was almost negligible after the 21-day incubation period. In contrast, distal tumors in the BSA-Man@Mn^2+^-Ft and BSA-Man-Ft@Lap groups showed significant growth. The trends of distal tumor growth in different groups indicated that the systemic immunity after Lap-mediated ICD or Mn^2+^-mediated cGAS-STING stimulation in DCs was insufficient to inhibit the progression of the poorly immunogenic distal tumors when acting separately, while the strategic coordination of the two modalities with BSA-Man@Mn^2+^-Ft@Lap could lead to almost complete remission of both the primary and distal tumors. We further collected murine lymph nodes and distal tumor tissues to detect the lymphatic DCs and tumor-specific effector T cell activation status for determining the mechanism of nanoassembly-mediated elimination of distal tumors. Flow cytometry results showed that the matured DC population in the lymph nodes of the BSA-Man@Mn^2+^-Ft@Lap group was around 21% higher than the PBS group (Fig. 9e), supporting the enhanced cross-presentation capacity of DCs after the BSA-Man@Mn^2+^-Ft@Lap-mediated immunomodulation. Meanwhile, the IFNγ secretion by tumor-resident effector T cells after BSA-Man@Mn^2+^-Ft@Lap administration increased by about 17% comparing to PBS group, immediately suggesting the nanoassembly-induced promotion of effector T cell activation and infiltration into tumor tissues (Fig. 9f).

The abscopal effect of the nanoassembly-mediated immunotherapy was further verified via metastasis analysis. The 4T1 tumor in this study is a highly aggressive tumor model prone to whole-body metastasis. Here we treated the 4T1-tumor-bearing mice with different samples for 21 days and then collected the lungs to monitor the 4T1 lung metastasis. As shown in Fig. 9g, h and Supplementary Fig. 28b, large amount of macroscopic and microscopic lung metastasis foci were found in the PBS, BSA-Man-Ft, BSA-Man-Ft@Lap, and BSA-Man@Mn^2+^-Ft groups, while the number of metastasis foci in the BSA-Man@Mn^2+^-Ft@Lap group was almost negligible. These observations supported our hypothesis that the nanoassembly-mediated cooperative DC-specific cGAS-STING stimulation and tumor-selective ICD induction was beneficial for activating robust tumor-specific T cell immunity against metastatic tumors.

## Discussion

In summary, we have developed a TME-activatable protein-based cooperative cGAS-STING nanoagonist for enhanced immunotherapy against poorly immunogenic solid tumor indications. The nanoagonist was obtained through the reversible cross-linking of Mn^2+^-anchored mannose-modified BSA and β-lapachone-loaded ferritin, which could readily disintegrate in the TME and selectively taken in by DCs and tumor cells. Lap would induce the immunogenic apoptosis of tumor cells and release abundant tumor-derived dsDNA into TME, which would be sensed by DCs via Mn^2+^-stimulated cGAS-STING signaling pathway to enhance the activation and infiltration of tumor-specific effector T cells, eventually leading to enhanced systemic antitumor immunity. In vivo analysis showed that the cooperative cAGS-STING nanoagonist caused almost complete remission of primary tumors, efficiently suppressed the growth of secondary distal tumors and substantially reduced tumor metastasis without eliciting systemic toxicity, which offers an approach for overcoming the immunoresistance of solid tumor indications.

## Methods

### Materials

Mannose, 4-carboxybenzaldehyde, bovine serum albumin (BSA), MnCl_2_, fluorescein isothiocyanate (FITC), and ferritin were purchased from Sigma-Aldrich. OH-PEG_2000_-OH, sodium hydroxide, 1-(3-dimethylaminopropyl)-3-ethylcarbodiimide hydrochloride (EDC), and N-hydroxy succinimide (NHS) were provided by Aladdin China. DCM, DMF, anhydrous alcohol diethyl, and methanol solution were provided by Xingang Chemical Glass Co. LTD (Chongqing). Annexin FITC/PI cell assay kit, ATP assay kit, FDA, PI, DCFH-DA, DAPI, and TUNEL kits were provided by Beyotime.

HMGB1, IL-6, TNF-α, IFN-β, IFN-γ, and IL-10 Elisa kit were provided by Fine TEXT. Antibodies used in this study include: GADPH (Proteintech, 10494-1-AP, 1:10,000), BAX (Proteintech, 50599-2-Ig, 1:1000), Bcl-2 (Proteintech, 26593-1-AP, 1:1000), HMGB1 (Proteintech, 66525-1-Ig, 1:1000), CRT (Proteintech, 10292-1-AP, 1:1000), HSP70 (Proteintech, 66183-1-Ig, 1:1000), NQO1 (Proteintech, 11451-1-AP, 1:5000), HRP-conjugated anti-rabbit secondary antibody (Proteintech, SA00001-2, 1:5000), Cy3-conjugated Goat anti-Rabbit IgG (Sangon Biotech, D110062, 1:300), Alexa Fluor 488-conjugated Goat anti-rabbit IgG (Sangon Biotech, D110061, 1:300). STING (Cell Signaling Technology, 13647, 1:1000), p-STING (Cell Signaling Technology, 50907, 1:1000), TBK1 (Cell Signaling Technology, 3504, 1:1000), p-TBK1 (Cell Signaling Technology, 5483T, 1:1000), IRF3 (Cell Signaling Technology, 29047, 1:1000), p-IRF3 (Cell Signaling Technology, 29047, 1:1000), IRF3 (Cell Signaling Technology, 4302, 1:1000). CD4 (Leinco Technologies, 53-6.7 C375), CD8 (Leinco Technologies, GK1.5 C1333). Fluorescent antibodies used in this study include: FITC-antiCD80 (Biolegend, 16-10A1, 50 μg), PE-antiCD86 (Biolegend, A17199A, 50 μg), APC-antiF4/80 (Biolegend, QA17A29, 100 μg), FITC-antiCD4 (Biolegend, KG1.5, 50 μg), PE-antiCD8 (Biolegend, S18018E, 50 μg), FITC-antiCD8 (Biolegend, 53-6.7), FITC-antiIFN-γ (Biolegend, XMG1.2), and APC-antiCD11c (Biolegend, N418, 100 μg). Antibodies for western blot analysis were diluted Primary Antibody Dilution Buffer. Antibodies for intraperitoneal injections were diluted using sterile PBS. The validation information for all antibodies was shown in Supplementary Table 1.

### Cell lines and animal handling

4T1 and 4T1-Luc cell lines were purchased from Yeze Shanghai Biological Technology Co. LTD. under the catalog number of CRL-2539 and CRL-2539-luc2, respectively. B16F10 and B16F10-Luc were provided by Chongqing Medical University under the catalog number of CRL-6475 and CRL-6475-luc2. STING-KO C57 mice were purchased from Gempharmatech Co., LTD. Balb/c and C57 mice (male, 6-week-old) were provided by Chongqing Medical University and all mice were kept in the animal house of Chongqing Medical University. Mice were housed in cages with four mice per cage and kept on in a regular 12-h:12-h light:dark cycle (9:00 AM–9:00 PM; 9:00 AM–9:00 PM). The temperature was 22 ± 1 degree Celsius) and humidity was 40–68%. All characterizations were carried out following the Animal Management Rules of the Ministry of Health of the People’s Republic of China.

### Synthesis of OHC-PEG_2000_-CHO

OH-PEG_2000_-OH (2.5 g), 4-carboxybenzaldehyde (1.125 g), EDC·HCl (4.793 g), and DMAP (0.122 g) were dissolved in a 250 mL round-bottom flask and added with 150 mL of dichloromethane (DCM) to dissolve the above reagents, which was then stirred for 48 h at 25 °C. Afterward, DCM was removed by rotary evaporation, the product was washed 5 times with saturated sodium chloride solution and another 3 times with 5% sodium chloride solution. Subsequently, the organic layer was collected and incubated with 50 mg anhydrous magnesium sulfate for 12 h to remove moisture after filtration. The product was recovered on a rotary evaporator and purified twice with excess diethyl ether. Then, the products were dialyzed (MWCO = 1000 Da) with ultrapure water for 2 d. After lyophilization, the final product OHC-PEG_2000_-CHO (75.2% yield) were obtained and characterized by ^1^H NMR (400 MHz, D_2_O, ppm).

### Synthesis of COOH-PEG_2000_-COOH

OH-PEG_2000_-OH (1 g), succinic anhydride (0.3 g), triethylamine (500uL), and DMAP (0.1 g) were dissolved in a 250 mL round-bottom flask and added with 150 mL of dichloromethane (DCM) to dissolve the above reagents, which was then stirred for 24 h at 25 °C. Afterward, DCM was removed by rotary evaporation, the products were dialyzed (MWCO = 600 Da) with ultrapure water for 2 d. After lyophilization, the final product COOH-PEG_2000_-COOH was obtained and characterized by ^1^H NMR (400 MHz, D_2_O, ppm).

### Synthesis of Man-COOH and BSA-Man

Mannan (100 mg) and sodium hydroxide (3 M, 1 mL) were dissolved in a round-bottom flask and stirred for 20 min for alkalinization. 1.2 mL of chloroacetic acid (160 g/L^−1^) was then added and the mixture was stirred in an oil bath (55 °C) for 7 h, After that, hydrochloric acid (1 M) was added to adjust the pH value to 2–3 to produce carboxymethylated mannan, which was precipitated with methanol. The samples were analyzed via HPLC and ^1^H NMR (400 MHz, D_2_O, ppm) tests. Man-COOH (0.84 mmol, 0.2 g), EDC (1.68 mmol, 0.32 g), and NHS (1.68 mmol, 0.2 g) were dissolved in water. The solution mixture was stirred for 6 h to activate carboxyl groups, followed by the addition of BSA (600 mg) dissolved in water. After stirring for 24 h, the BSA-Man product was purified by dialyzing (MWCO = 1000 Da) against DI water for 2 days.

### Fluorescamine assay on BSA and BSA-Man

First, BSA-COOH and BSA were dissolved with 1 mL of PBS (pH 8.0) at a concentration of 100 μg/mL in a 1.5 mL tube, followed by the addition of fluorescamine solution (40 μL, 0.3 g/L) for 20 min incubation under dark environment. Finally, the fluorescence intensity was measured on a fluorescence spectrophotometer.

### Anchoring manganese ions into BSA-Man

BSA-Man (3 mg) was dissolved in 1 mL of deionized water and then mixed with different concentrations of MnCl_2_ (0, 2, 4, 6, 8, 10, 12, 14, and 16 µg/mL). The incubation would continue for 6 h in an incubator under 37 °C. Finally, the fluorescence emission intensity of BSA-Man@Mn^2+^ at 285 nm under the excitation at 285 nm was measured by fluorescence spectrophotometer to determine the optimal condition to obtain BSA-Man@Mn^2+^ nanocomplex.

### Cross-linking of BSA-Man@Mn^2+^ and Ft@Lap

First, BSA-Man (3 mg/mL, 10 mL) and MnCl_2_ (16 µg/mL, 3 mL) was dissolved with PBS (10 mL, pH7.8) in a 25 mL round-bottom flask and stirred for 12 h. The mixture solution was dialyzed (MWCO = 1000 Da) to remove the excess manganese ions and then dried with cryogenic freeze dryer to obtain BSA-Man@Mn^2+^. Ft (5 mg/mL, 5 mL) and Lap (100 μg/mL, 3 mL) were dissolved with deionized water (10 mL) and DFM (500 µL) in a 25 mL round-bottom flask and stirred 12 h to obtain Ft@Lap complex. Subsequently, 50 mg BSA-Man@Mn^2+^ was added into a flat-bottomed flask and mixed with 10 mL ultrapure water, followed by ultrasonication to dissolve the contents. The solution was then stirred at 300 rpm and mixed with 10 mg of Ft@Lap dissolved in 2 mL of ultrapure water in a 50 mL centrifuge tube. Subsequently, NaOH (1 M, 20 µL) was injected into the tube, followed by the dropwise addition of alcohol. When the mixture solution changed to a colloidal state after around 2 h, 100 µL of CHO-PEG_2000_-CHO was added to enable Schiff base ligation, which lasted around 6 h. After the reaction, the solution was purified via dialysis (MWCO: 5000) against deionized water for 2 days.

### Synthesis of BSA-Man@Mn^2+^-Ft-FITC@Lap

Ft@Lap (5 mg) and FITC (3 mg) were dissolved in a single-neck round-bottom flask using 5 mL of PBS and stirred at room temperature for 24 h at 600 rpm. Afterward, the solution was poured into a dialysis bag to remove the residue FITC to obtain Ft-FITC@Lap. 50 mg of BSA-Man@Mn^2+^ and 10 mg of Ft-FITC@Lap were dissolved in a flat-bottomed flask, followed by the addition of 10 μL NaOH (1 M). Subsequently, ethyl alcohol was added into the solution dropwise under stirring until the mixture became turbid. 100 µL of CHO-PEG_2000_-CHO was added for cross-linking and the reaction lasted another 6 h. After the reaction, the solution was transferred to a dialysis bag with an MWCO of 5000 for 2 days. BSA-Man@Mn^2+^-Ft- FITC@Lap was thus obtained.

### Synthesis of BSA-FITC-Man@Mn^2+^-Ft@Lap

BSA-Man@Mn^2+^ (10 mg) and FITC (5 mg) were dissolved in a single-neck round-bottom flask using 5 mL of PBS, stirred at room temperature for 24 h at 600 rpm, and then poured into a dialysis bag to remove the residue FITC to obtain BSA-FITC-Man@Mn^2+^, which was further used to synthesize BSA-FITC-Man@Mn^2+^-Ft@Lap.

### Synthesis of BSA-Man@Mn^2+^-Ft@Cy5

Ft (10 mg), Cy5 (10 mg), NHS (3 mg), and EDC (3 mg) were dissolved in a single-neck round-bottom flask using 5 mL of PBS, stirred at room temperature for 24 h and Ft@Cy5 was prepared through the amide ligation. BSA-Man@Mn^2+^-Ft@Cy5 are synthesized by cross-linking with BSA-Man@Mn^2+^and Ft@Cy5 in the manner described above.

### Synthesis of BSA-FITC-Man@Mn^2+^-Ft-RhB@Lap

BSA-Man@Mn^2+^ (10 mg) and FITC (5 mg) were dissolved in a single-neck round-bottom flask using 5 mL of PBS, stirred at room temperature for 24 h at 600 rpm, and then poured into a dialysis bag to remove the residue FITC to obtain BSA-FITC-Man@Mn^2+^; Ft (5 mg/mL, 5 mL), Lap (100 μg/mL, 3 mL) and RhB (Rhodamine) (50 μg/mL, 100 mL) were dissolved with deionized water (10 mL) and DFM (500 µL) in a 25 mL round-bottom flask and stirred 12 h to obtain Ft-RhB@Lap complex. Subsequently, BSA-FITC-Man@Mn^2+^-Ft--RhB @Lap are synthesized by cross-linking with BSA-FITC-Man@Mn^2+^ and Ft-RhB@Lap in the manner described above.

### pH-triggered dissociation of the protein assembly

BSA-Man@Mn^2+^-Ft@Lap was dissolved in 1 mL of PBS (pH 6.5) and incubated on a shaker for different periods (0, 6, and 12 h). Morphology of the nanoassembly was observed by TEM.

### UV absorption

The UV absorption of Lap, Ft, Ft@Lap, BSA-Man@Mn^2+^, and BSA-Man@Mn^2+^-Ft@Lap in the range of 200–500 nm was measured at an equivalent Lap concentration of 3.5 μg/mL on a UV spectrometer.

### DLS characterization of the protein assembly

BSA-Man@Mn^2+^, Ft@Lap, and BSA-Man@Mn^2+^-Ft@Lap were dissolved in pure water at 1 mg/mL. The hydrodynamic sizes of the nanosamples were measured using a multi-angle particle size analyzer (Brookhaven Instruments Corporation, Omni).

### DLS characterization on the serum stability of protein assembly

To further investigate the blood stability of the nanoassembly after systemic administration, mouse blood was collected after removing the eyeballs and centrifugated at 670.8 × *g* for 10 min without anti-coagulant to collect serum. The fresh murine serum was diluted in PBS at a volume ratio of 1:9 for the solubilization of BSA-Man@Mn^2+^-Ft@Lap at 1 mg/mL. The hydrodynamic sizes and PDI of the nanoassembly were measured at 12 h, 24 h, 36 h, and 48 h using a multi-angle particle size analyzer (Brookhaven Instruments Corporation, Omni).

### Evaluation of Lap and Mn^2+^ release in solution

30 mg of BSA-Man@Mn^2+^-Ft@Lap was dissolved 30 mL of PBS with different pH values (pH = 7.4, pH = 7.4 + 0.28 μg/L Cathepsin B, pH = 6.5, pH = 6.5 + 0.28 μg/L Cathepsin B, pH = 5.5, pH = 5.5 + 0.28 μg/L Cathepsin B) and then poured in a dialysis bag, which was placed in an incubator at 37 °C. 0.2 mL of the incubation solution was collected at different time points and measured by ultraviolet-visible spectroscopy and inductive coupled plasma emission spectrometry to test the Lap and Mn^2+^ release, respectively.

### CLSM study on tumor cell uptake

4T1 cell were seeded into confocal dishes at a density of 1 × 10^5^ and incubated overnight. Afterward, the culture medium was replaced with fresh ones containing 100 μg/mL of BSA-Man@Mn^2+^-Ft-FITC@Lap, while the pH value of medium was adjusted to 7.4 and 6.5, respectively. The incubation would continue for another 12 h/24 h in an incubator, afterward the residue nanosamples were washed away using PBS for 3 times. Cells were fixed with 4% paraformaldehyde for 30 min at 4 °C, washed with PBS for 3 times, added with 200 mL of rhodamine-labeled wheat germ agglutinin (10 μg/mL) and incubated overnight at 4 °C under dark. 200 mL DAIP was subsequently added to stain the cell nucleus. Finally, the double-stained cells were washed with PBS for 3 times and mounted with glycerol, and then observed under a confocal fluorescence microscope (Leica TCS SP8, Germany).

### Flow cytometry analysis on the uptake of the nanoagonist by tumor cell

4T1 cells were seeded into 6-well plates at a density of 1 × 10^5^ and incubated overnight. To investigate the role of Ft-TfR1 binding on the uptake rates of the Ft units, some of the 4T1 tumor cells were pre-treated with TfR1 antibody (0.5 µL) for 6 h, afterward non-treated and TfR1 antibody-pre-treated 4T1 cells were incubated using fresh culture media supplemented with BSA-Man@Mn^2+^-Ft-FITC@Lap (100 μg/mL) for another 12 h and 24 h of incubation at pH 7.4 or pH 6.5. The 4T1 tumor cells were collected and the cellular FITC intensity was analyzed via flow cytometry (Beckman Coulter).

### Fluorescent analysis on the targeting ability of individual components in the nanoassemblies

To start with, BSA and Ft units were labeled with FITC via thiourea ligation. The FITC-labeled BSA and Ft units were used to construct BSA-FITC-Man@Mn^2+^-Ft@Lap and BSA-Man@Mn^2+^-Ft-FITC@Lap nanoassemblies. Meanwhile, 4T1 cells were seeded into 6-well plates at a density of 1 × 10^5^ in phenol red-free DMEM and incubated overnight, afterward the culture medium was replaced with fresh ones containing (I) BSA-FITC-Man@Mn^2+^-Ft@Lap and (II) BSA-Man@Mn^2+^-Ft-FITC@Lap at an equivalent concentration of 100 μg/mL for 24 h of incubation at pH 6.5. The change in the FITC fluorescence in supernatant was measured by fluorescence spectrophotometer to indicate the cellular uptake of individual protein units.

### Flow cytometry analysis on the uptake of the nanoagonist by BMDCs

Murine bone marrow cells were inoculated in 6-well plates overnight after extraction and treated by the medium containing IL-4 (10 ng/mL) and GM-CSF (20 ng/mL) to generate BMDCs. BMDCs were isolated after 7 days by collecting the floating cells. The purified BMDCs were seeded into 6-well plates at a density of 1 × 10^6^ units per well and incubated overnight, afterward fresh culture media supplemented with BSA-FITC@Mn^2+^-Ft@Lap, BSA-Man-FITC@Mn^2+^-Ft@Lap (100 μg/mL), or BSA-Man-FITC@Mn^2+^-Ft@Lap (100 μg/mL)+CD206 antibody (0.5 µL) were added for another 24 h of incubation at pH 7.4 or pH 6.5. The BMDCs were collected for flow cytometry analysis (Beckman Coulter).

### Flow cytometric analysis on the apoptosis levels of DCs

4T1 cell were seeded into 6-well plate at a density of 1 × 10^5^ and incubated overnight. Afterward, the culture medium was replaced with fresh ones containing (I) PBS, (II) BSA-Man-Ft, (III) BSA-Man-Ft@Lap, (IV) BSA-Man@Mn^2+^-Ft, and (V) BSA-Man@Mn^2+^-Ft@Lap at an equivalent concentration of 100 μg/mL, while the pH value of medium was adjusted to 6.5. The supernatant of the nanoassembly-treated tumor cells was collected after 24 h and used for the incubation of BMDCs in a cell incubator for 24 h. Finally, the treated BMDCs were purified and resuspended in 200 μL of sterile PBS, followed by assaying with Annexin V/PI Cell Assay Kit to determine their apoptosis levels.

### MTT assay on the chemotherapeutic efficacy of the nanoassembly

4T1 cells were inoculated into 96-well plates at a density of 1 × 10^4^ and incubated overnight. The culture medium was replaced with fresh ones containing (I) PBS, (II) BSA-Man-Ft, (III) BSA-Man-Ft@Lap, (IV) BSA-Man@Mn^2+^-Ft, and (V) BSA-Man@Mn^2+^-Ft@Lap at an equivalent nanoassembly concentration of 100 μg/mL, while the pH value of medium was adjusted to 6.5. The incubation lasted for 12 h or 24 h at 37 °C under an atmospheric CO_2_ level of 5%, respectively. After removing the supernatants, cells were incubated with fresh medium containing MTT agent (0.5 mg/mL) for another 4 h at 37 °C, followed by the addition of 100 μL DMSO to dissolve the emerging Formazan crystals. The plates were measured on a SpectraMax I3X microporous plate reader to determine the optical density at 490 nm.

### Flow cytometry analysis on nanoagonist-mediated tumor cell eradication in vitro

4T1 cells (10^5^ units) and immune cells extracted from the spleen of mouse (10^6^ units) were co-inoculated in the 6-well plate overnight, afterward the culture medium was replaced with fresh ones containing (I) PBS, (II) BSA-Man-Ft, (III) BSA-Man-Ft@Lap, (IV) BSA-Man@Mn^2+^-Ft, and (V) BSA-Man@Mn^2+^-Ft@Lap. The samples were supplemented at the concentration of 100 μg/mL, while the pH value of medium was adjusted to 6.5. After incubation for 24 h, the floating immune cells were removed by discarding the exhausted culture media, while the tumor cells were detached with trypsin, cleaned with PBS for 3 times, and collected by centrifugation. Cells were resuspended with 200 μL of sterile PBS, followed by treatment with Annexin V/PI Cell Assay Kit. CytoFLEX system (Beckman Coulter) was used to detect tumor cell apoptosis due to the combined treatment of nanoassembly and immune cells.

### Cell live/death imaging

4T1 cells and immune cells extracted from the spleen of mice were inoculated into confocal dishes overnight, afterward the culture medium was replaced with fresh ones containing (I) PBS, (II) BSA-Man-Ft, (III) BSA-Man-Ft@Lap, (IV) BSA-Man@Mn^2+^-Ft, and (V) BSA-Man@Mn^2+^-Ft@Lap at the equivalent concentration of 100 μg/mL, while the pH value of medium was adjusted to 6.5. After incubation for 24 h, cells attached on the dishes were washed with PBS for 3 times and incubated with FDA (50 μg/mL) for 30 min. Then the cells were again washed with PBS for 3 times and treated with propidium iodide (400 μg/mL) in dark for 10 min. The cells were washed with PBS for another 3 times and observed with Leica TCS SP8 confocal laser microscopy at ×40.

### CLSM Imaging on immunogenic apoptosis of tumor cells

4T1 cells were seeded in dishes at a density of 1 × 10^5^ cells. Subsequently, the culture medium was replaced with fresh ones containing (I) PBS, (II) BSA-Man-Ft, (III) BSA-Man-Ft@Lap, (IV) BSA-Man@Mn^2+^-Ft, and (V) BSA-Man@Mn^2+^-Ft@Lap at the equivalent concentration of 100 μg/mL, while the pH value of medium was adjusted to 6.5 for 24 h of incubation. The treated cells were gently washed with PBS for 3 times and then stained with the antibodies of CRT and HMGB1 and incubated overnight at 4 °C. Fluorescent secondary antibodies were subsequently added for incubation at 37 °C for 2 h, followed by staining with DAPI for 10 min. Finally, the samples were washed with PBS and observed by a Leica TCS SP8 confocal laser microscope.

### ELISA assay of ATP and HMGB1 release in 4T1 cells after treatment with different samples

4T1 cells were first inoculated into the 6-well plate overnight. Subsequently, the culture media were replaced with 2 mL of fresh ones including (I) control, (II) BSA-Man-Ft, (III) BSA-Man-Ft@Lap, (IV) BSA-Man@Mn^2+^-Ft, and (V) BSA-Man@Mn^2+^-Ft@Lap at the equivalent concentration of 100 μg/mL, while the pH value of medium was adjusted to 6.5 and further incubated for 24 h. Afterward, the samples were centrifuged at low speed of 201.2 g/min and the supernatant in each group was collected; in which the ATP and HMGB1 levels were determined by ATP enhancement test kit and HMGB1 ELISA kit.

### Western blot analysis of 4T1 tumor cells and DCs

First, 4T1 cells were inoculated into a 6-well plate at a density of 1 × 10^5^ overnight. There were five experimental groups including (I) control, (II) BSA-Man-Ft, (III) BSA-Man-Ft@Lap, (IV) BSA-Man@Mn^2+^-Ft, and (V) BSA-Man@Mn^2+^-Ft@Lap. The concentration of the nanoassemblies was maintained at an equivalent level of 100 μg/mL, while the pH value of medium was adjusted to 6.5. Cells were incubated with nanoassemblies for 24 h. Afterward, the tumor cells were collected and the total protein concentration was determined by BCA protein assay kit. Protein immunoassay was performed by SDS-PAGE electrophoresis and finally photographed by a molecular imaging apparatus (Versa doc MP 4000 system, Bio-Rad). The expression levels of CRT, HMGB1, HSP70, BAX, Bcl-2 were observed and analyzed using GADPH as the internal standard.

For the western blot analysis on DCs, 4T1 cells were incubated overnight in 6-well plates at an initial density of 1 × 10^5^ and treated with culture media supplemented with (I) control, (II) BSA-Man-Ft, (III) BSA-Man-Ft@Lap, (IV) BSA-Man@Mn^2+^-Ft, (V) BSA-Man@Mn^2+^-Ft@Lap, (VI) BSA-Man@Mn^2+^-Ft@Lap+dsDNase (100 µL), and (VII) BSA-Man@Mn^2+^-Ft@Lap+HMGB1 antibody (0.5 µL) for 24 h. The nanoassembly concentration was maintained at an equivalent level of 100 μg/mL and the pH of the culture medium was set at 6.5. Supernatants from 4T1 cells were collected for BMDC incubation for another 24 h, for which the BMDCs were seeded in 6-well plates at a density of 8 × 10^6^ and pre-incubated for 24 h. The supernatant-treated BMDCs were collected for WB analysis to detect STING, p-STING, TBK1, p-TBK1, IRF-3, and p-IRF-3 levels.

### Flow cytometry analysis on the uptake of ds DNA by DCs

Bone marrow of murine tibia and fibula were extracted and the bone marrow cells were incubated with IL-4 (10 ng/mL) and GM-CSF (20 ng/mL), which would differentiate into BMDCs and were collected after 7 days of induction. Meanwhile, 4T1 cells were seeded in 6-well plates and treated by (I) control, (II) BSA-Man-Ft, (III) BSA-Man-Ft@Lap, (IV) BSA-Man@Mn^2+^-Ft, (V) BSA-Man@Mn^2+^-Ft@Lap, (VI) BSA-Man@Mn^2+^-Ft@Lap+DNase, and (VII) BSA-Man@Mn^2+^-Ft@Lap+HMGB1 antibody at pH 6.5 for 24 h. The supernatants for each group were collected as the incubation media for BMDCs, which would last 24 h. BMDCs were finally collected, fixed, and permeabilized for flow cytometry analysis to determine the amount of dsDNA uptake.

### Flow cytometry analysis on the activation status of DCs and T cells

4T1 cells (1 × 10^5^ units) and murine splenic immune cells (3 × 10^6^ units) were seeded in 6-well plates and incubated overnight. Fresh culture media containing (I) control, (II) BSA-Man-Ft, (III) BSA-Man-Ft@Lap, (IV) BSA-Man@Mn^2+^-Ft, and (V) BSA-Man@Mn^2+^-Ft@Lap (2 mL, 100 μg/mL) were then added for treatment, while the pH value of medium was adjusted to 6.5. The cells were placed in an incubator for further cultivation for 24 h, afterward the floating immune cells were collected and stained with fluorescent antibodies as follows: PE anti-CD86 (Biolegend Co., USA), FITC anti-CD80 (Biolegend Co., USA), APC anti-F4/80 (Biolegend Co., USA), APC anti-CD11c (Biolegend Co., USA), FITC anti-CD4 (Biolegend Co., USA), PE anti-CD8 (Biolegend Co., USA), FITC anti-IFN-γ (Biolegend Co., USA). Finally, samples were tested by flow cytometry (Beckman Coulter Co., USA).

### Determining the contribution of different antigen-sensing mechanisms for nanoagonist-mediated immunostimulatory effects

4T1 cells (1 × 10^5^ units) and murine splenic immune cells (3 × 10^6^ units) were seeded in 6-well plates and incubated overnight. The DC maturation and T cell activation status with or without TLR4 inhibition was then monitored for comparatively analysis. For the first batch of samples, the cells were incubated with fresh culture media containing (I) control, (II) BSA-Man-Ft, (III) BSA-Man-Ft@Lap, (IV) BSA-Man@Mn^2+^-Ft, and (V) BSA-Man@Mn^2+^-Ft@Lap (2 mL, 100 μg/mL). In comparison, cells of the second batch were pre-treated with TLR4 antibodies for 6 h before the addition of (I) control, (II) BSA-Man-Ft, (III) BSA-Man-Ft@Lap, (IV) BSA-Man@Mn^2+^-Ft, and (V) BSA-Man@Mn^2+^-Ft@Lap (2 mL, 100 μg/mL). The pH value of medium was adjusted to 6.5 for all groups. The cells were placed in an incubator for further cultivation for 24 h, afterward immune cells from supernatant were collected and stained with fluorescent antibodies as follows: PE anti-CD86 (Biolegend Co., USA), FITC anti-CD80 (Biolegend Co., USA), APC anti-F4/80 (Biolegend Co., USA), APC anti-CD11c (Biolegend Co., USA), FITC anti-CD4 (Biolegend Co., USA), PE anti-CD8 (Biolegend Co., USA), FITC anti-IFN-γ (Biolegend Co., USA). Finally, samples were tested by flow cytometry (Beckman Coulter Co., USA).

### ELISA Assay on the biochemical alterations of nanoagonist-stimulated immune cells

To explore the treatment-induced cGAMP production in BMDCs, 1 × 10^5^ units of 4T1 cells were first incubated overnight in 6-well plates and then treated by (I) control, (II) BSA-Man-Ft, (III) BSA-Man-Ft@Lap, (IV) BSA-Man@Mn^2+^-Ft, (V) BSA-Man@Mn^2+^-Ft@Lap, (VI) BSA-Man@Mn^2+^-Ft@Lap+DNase, and (VII) BSA-Man@Mn^2+^-Ft@Lap+HMGB1 antibody under pH 6.5. The incubation lasted for 24 h, and the supernatant was collected for the incubation of BMDCs for 24 h. BMDCs were finally collected and their cGAMP production levels were tested with c-GAMP ELISA kits.

To test the cytokine secretion levels of the activated immune cells, 4T1 cells (10^5^ units) and murine splenic immune cells (10^6^ units) were co-inoculated in the 6-well plate overnight, afterward the culture media were replaced with fresh ones containing (I) control, (II) BSA-Man-Ft, (III) BSA-Man-Ft@Lap, (IV) BSA-Man@Mn^2+^-Ft, and (V) BSA-Man@Mn^2+^-Ft@Lap (2 mL, 100 μg/mL) at the pH of 6.5. Cells was placed in an incubator for further cultivation for 24 h and the supernatant was subsequently collected. The secretion levels of cytokines including TNF-α, IL-6, IL-10, and IFN-γ in the samples were tested with ELISA kits (Dakewe Biotech, China).

### Construction of animal models and therapeutic evaluation

60 male Balb/c and C57 mice were purchased and housed in Chongqing Medical University for the in vivo evaluations in this study, of which the average age and weight were around 4–6 weeks and 18 g. All animal tests have been reviewed and approved by the Animal Care and Use Committee of Laboratory Animals Administration of Chongqing Medical University, which strictly followed the national and institutional guidelines. According to the national and institutional guidelines, the maximum tumor size and weight allowed was 3 cm^3^ and 10% of mouse body weight. Mice were euthanized when the tumor burden exceeded the threshold. Each Balb/c mouse was injected with PBS solution containing 4 × 10^7^ units of 4T1-luc cells to establish the 4T1-luc tumor mouse model, while B16F10-luc tumor mouse model was established by injecting same number of B16F10-luc cells into C57 mice. When the tumor grew to about 100 mm^3^, the tumor-bearing Balb/c or C57 mice were randomly divided into 5 groups (6 mice in each group), while body weight and tumor volume of each group were kept at a comparable level with the same batch of mice. For each type of tumor models, there are five groups including (I) control, (II) BSA-Man-Ft, (III) BSA-Man-Ft@Lap, (IV) BSA-Man@Mn^2+^-Ft, and (V) BSA-Man@Mn^2+^-Ft@Lap, respectively. The tumor size was measured once every three days, which was calculated using the formula Vtumor = L × W^2^/2 (L: longitudinal diameter of the tumor, W: cross-sectional diameter of the tumor). After 21 days of treatment, the mice were euthanized and tumor and main organs were exercised and washed with PBS for 3 times, prepared into paraffin sections and then stained for H&E and TUNEL assays. Immune cells were extracted from the tumor and spleen, followed by grinding and processing using various fluorescently labeled antibodies including FITC anti-CD80 (Biolegend Co., USA), PE anti-CD86 (Biolegend Co., USA), APC anti-F4/80 (Biolegend Co., USA), APC anti-CD11c (Biolegend Co., USA), FITC anti-MHC I, PE anti- MHC II (Biolegend Co., USA), FITC anti-CD4 (Biolegend Co., USA), PE anti-CD8 (Biolegend Co., USA), FITC anti-IFN-γ (Biolegend Co., USA) for flow cytometry analysis (Beckman Coulter Co., USA). The tumors were also dissected and sliced into thin sections after formaldehyde fixation, which were processed using 50 µL of PE anti-CD4 (Biolegend Co., USA), PE anti-CD8 (Biolegend Co., USA), FITC anti-IFN-γ (Biolegend Co., USA) and incubated in a refrigerator at 4 °C for 8 h to monitor the tumor infiltration of immune cells via immunofluorescence imaging. Meanwhile, the serum was separated from the blood of mice and the content of cytokines (IFN-β, IFN-γ, TNF-α, and IL-6) in the serum was detected by ELISA kit.

### In vivo imaging for biodistribution analysis

To observe the distribution of nanoassemblies in mice after systemic administration, dissociable Schiff base-ligated BSA-Man@Mn^2+^-Ft@Lap@Cy5 (cross-linked with CHO-PEG_2000_-CHO) and non-dissociable amide-ligated BSA-Man@Mn^2+^-Ft@Lap@Cy5 (cross-linked with COOH-PEG_2000_-COOH) were prepared and injected into the tumor-bearing mice through tail vein at a concentration of 3 mg/kg/d and then imaged at 6 h, 12 h and 24 h using a living-image system.

### Blood circulation stability test

BSA-Man@Mn^2+^-Ft@Lap@Cy5 and Cy5 were injected into the tail vein of the mice at a concentration of 3 mg/kg/d. Afterward, 100 μL of blood was extracted from the tail at 0, 0.5, 1, 2, 4, 6, 8, 12, and 24 h post injection. Supernatant was collected after centrifugation at 670.8 g. The fluorescence value in supernatant in the BSA-Man@Mn^2+^-Ft@Lap@Cy5 group at 0 h was set as 100%, and the relative Cy5 fluorescence intensity in supernatant was detected on a fluorescence spectrometer at 0.5, 1, 2, 4, 6, 8, 12, and 24 h, respectively. The blood circulation half-life was determined using Origin Pro.

### Evaluation of the DC/tumor cell dual-targeting capability of the nanoassembly in vivo

For the dual fluorescence labeling of the nanoagonist, BSA-Man@Mn^2+^ (10 mg) and FITC (5 mg) were dissolved in a single-neck round-bottom flask using 5 mL of PBS, stirred at room temperature for 24 h at 600 rpm, and then poured into a dialysis bag to remove the residue FITC to obtain BSA-FITC-Man@Mn^2+^ while Ft (5 mg/mL, 5 mL), Lap (100 μg/mL, 3 mL), and RhB (Rhodamine B) (50 μg/mL, 100 μL) were dissolved with deionized water (10 mL) and DFM (500 µL) in a 25 mL round-bottom flask and stirred 12 h to obtain Ft-RhB@Lap complex. The FITC-labeled BSA and RhB-labeled Ft units were then cross-linked using CHO-PEG_2000_-CHO and COOH-PEG_2000_-COOH to obtain dissociable and non-dissociable nanoagonists. 3 mg/kg of the amide-ligated BSA-Man@Mn^2+^-FITC-Ft@Lap@RhB and Schiff base-ligated BSA-Man@Mn^2+^-FITC-Ft@Lap@RhB were injected into the tail vein of tumor-bearing mice and incubated for 24 h, then the mice were euthanized and tumors were immediately dissected. Fresh frozen sections were fixed with paraformaldehyde and their fluorescence distribution patterns were observed by a Leica TCS SP8 confocal laser microscope. Meanwhile, 0.2 g of the tumors were grinded and the extracted cells were treated using fluorescently labeled antibodies including APC anti-CD11c (Biolegend Co., USA) and APC anti-CD45 (Biolegend Co., USA) for the FACS-sorting of DCs and tumor cells by FACS (Beckman Coulter Co., USA), in which the changes in FITC and RhB fluorescence levels were analyzed via flow cytometry.

### Survival analysis

The 4T1-luc and B16F10-luc tumor-bearing mice were randomly divided into 5 groups and treated by (I) control, (II) BSA-Man-Ft, (III) BSA-Man-Ft@Lap, (IV) BSA-Man@Mn^2+^-Ft, and (V) BSA-Man@Mn^2+^-Ft@Lap as described above. After 21 days of continuous treatment, the number of live mice in each group was record once every 3 days until day 65.

### Liver, kidney, and hematological indices of mice

The tumor-bearing mouse model was constructed by subcutaneous injection of 4T1 cells as described above, while different samples were administered in tail vein. Mouse blood was collected after 24 h from the eye socket and centrifugated at 670.8 × *g* for 10 min without anti-coagulant to collect serum. 200 µL serum was tested with AST/ALT/UREA/CREA assay kit to determine the liver and kidney functions. Meanwhile, 800 μL of the extracted blood was stored in anti-coagulant EP and tested on a hematology analyzer.

### Immunofluorescence analysis of tumor samples

After 21 days of treatment, the mice were euthanized and tumors were extracted, sliced into thin sections, and incubated with CRT, HMGB1, and ISG56 antibodies overnight. The old incubation media were removed and then added with fluorescent secondary antibodies for 4 h incubation at 4 °C, followed by staining with DAPI for 10 min. Finally, the samples were washed with PBS and observed by a Leica TCS SP8 confocal laser microscope.

### Bilateral tumor model construction and therapeutic evaluation

Mice with an average weight of about 18 g were divided into five groups (*n* = 6) and injected with 4T1-luc tumor cells on the left buttocks of the mice. When the tumor volume reached 100 mm^3^, they were injected with and treated by (I) control, (II) BSA-Man-Ft, (III) BSA-Man-Ft@Lap, (IV) BSA-Man@Mn^2+^-Ft and (V) BSA-Man@Mn^2+^-Ft@Lap through the caudal vein. A total of 7 injections were carried out at a three-day interval. Afterward, a second tumor was inoculated on the right hip of the mouse, of which the size changes were recorded regularly. After 21 days, the lymph gland and distal tumors in each group were extracted. Immune cells in the lymph gland and distal tumors were extracted by grinding, which were stained with various fluorescent antibodies as follow: FITC anti-APC (Biolegend Co., USA), PE anti-CD86 (Biolegend Co., USA), APC anti-F4/80 (Biolegend Co., USA), APC anti-CD11c (Biolegend Co., USA), FITC anti-CD8(Biolegend Co., USA), PE anti-IFN-γ (Bioligand Co., USA). Finally, samples were tested by flow cytometry (Beckman Coulter Co., USA).

### Evaluation of lung metastasis

When the 4T1-luc tumors grew to about 100 mm^3^, the mice were randomly divided into 5 groups with roughly equal tumor size and body weight, which were subsequently treated by (I) control, (II) BSA-Man-Ft, (III) BSA-Man-Ft@Lap, (IV) BSA-Man@Mn^2+^-Ft, and (V) BSA-Man@Mn^2+^-Ft@Lap through tail vein injection for 7 times at a 3-day interval. On day 50, the mice were sacrificed to extract the lungs, which were dissected and imaged to count the number of lung metastasis nodules. Meanwhile, the lungs were also embedded into paraffin and cut into thin sections for H&E staining.

### T cell depletion

25 male 4T1-luc tumor-bearing Balb/c mice with an initial tumor size of around 100 mm^3^ divided into 5 groups and treated by (I) control, (II) BSA-Man@Mn^2+^-Ft@Lap+antiCD4 + antiCD8, (III) BSA-Man@Mn^2+^-Ft@Lap+antiCD4, (IV) BSA-Man@Mn^2+^-Ft@Lap+antiCD8 and (V) BSA-Man@Mn^2+^-Ft@Lap, respectively. For group II, III, and IV, the mice were first treated with 100 µg of antiCD4 (clone GK1.5, BioXCell), 100 µg of antiCD8 (clone 2.43, BioXCell), and 100 µg of antiCD4 + 100 µg of antiCD8 through intraperitoneal injection, while the nanoagonist was injected through the tail vein the next day. The treatment was given at a 3-day interval and repeated for 7 times. Finally, the mice were euthanized after 21 days of treatment. the tumor was extracted and grinded, and antibodies (FITC anti-CD80, PE anti-CD86, APC anti-F4/80, FITC anti-CD4, PE anti-CD8, and anti-IFN-γ (Biolegend Co., USA)) were used to detect the infiltration of immune cells therein.

### Mechanistic analysis on STING-KO mice

Male STING-KO C57 mice with an average body weight of 15 ± 2 g and age of 6 weeks were purchased from Gempharmatech Co., LTD. Eight male STING-KO C57 mice provided by Gempharmatech Co., LTD were seeded with B16F10 tumors and divided into two groups of equal number (*n* = 4), which were incubated with or without BSA-Man@Mn^2+^-Ft@Lap. Meanwhile, 8 male WT C57 mice were treated similarly and used as wild-type control. Thus, there are altogether four groups for the mechanistic evaluations on nanoagonist-mediated cGAS-STING signaling including (I) STING^−/−^, (II) STING^+/+^, (III) STING^−/−^ + BSA-Man@Mn^2+^-Ft@Lap, and (IV) STING^+/+^ + BSA-Man@Mn^2+^-Ft@Lap, for which the treatment of BSA-Man@Mn^2+^-Ft@Lap would start when the tumor size reached around 100 mm^3^. After 21 days of treatment, the mice were euthanized and the tumors were grinded. Changes in the tumor infiltration and function of immune cells were detected by flow cytometry.

### Statistics and reproducibility

All measurements were performed on 3 or more independent replicates from separate experiments. The exact sample size and statistical test for each experiment are described in the relevant figure legends. All results are presented as the mean ± standard error (S.E.M.). All statistical data were processed in GraphPad Prism (version 8.0 for Windows) by Student’s *t*-test, one-way ANOVA, or two-way ANOVA. * Indicates significance at *p* < 0.05, ** indicates significance at *p* < 0.01, *** indicates significance at *p* < 0.001, **** indicates significance at *p* < 0.0001.

### Reporting summary

Further information on research design is available in the Nature Research Reporting Summary linked to this article.

## Supplementary information

Supplementary Information

Reporting Summary

## Data Availability

The publicly available BRCA data used in this study are collected from TCGA and GTEx databases and recomputed using UCSC Xena [https://xena.ucsc.edu/]. All data generated in this study are available within the Article, Supplementary Information or Source data file. Source data are provided with this paper.

## Source data

Source Data
