# Supplementary material for: A protein-based cGAS-STING nanoagonist enhances T cell-mediated anti-tumor immune responses
Source: Nat Commun. 2022 Sep 28;13:5685. doi: 10.1038/s41467-022-33301-0 (PMC9515186; doi:10.1038/s41467-022-33301-0)
Supplement: Supplementary file 1 — Supplementary Information [file 41467_2022_33301_MOESM1_ESM.pdf]

A protein-based cGAS-STING nanoagonist enhances T cell-mediated anti-tumor immune responses

Xuan Wang[1†], Yingqi Liu[1†], Chencheng Xue[1], Yan Hu[2], Yuanyuan Zhao[1], Kaiyong Cai[2], Menghuan Li[1*], Zhong Luo[1,2*]

[1]School of Life Science, Chongqing University, Chongqing 400044, P. R. China.

[2]Key Laboratory of Biorheological Science and Technology, Ministry of Education, Chongqing University, Chongqing 400044, P. R. China.

Author information:

[†]These authors contributed equally: Xuan Wang and Yingqi Liu.

[*]These authors jointly supervised this work: Menghuan Li and Zhong Luo.

Email:

Zhong Luo: luozhong918@cqu.edu.cn

Menghuan Li: menghuanli@cqu.edu.cn

Supplementary Figures

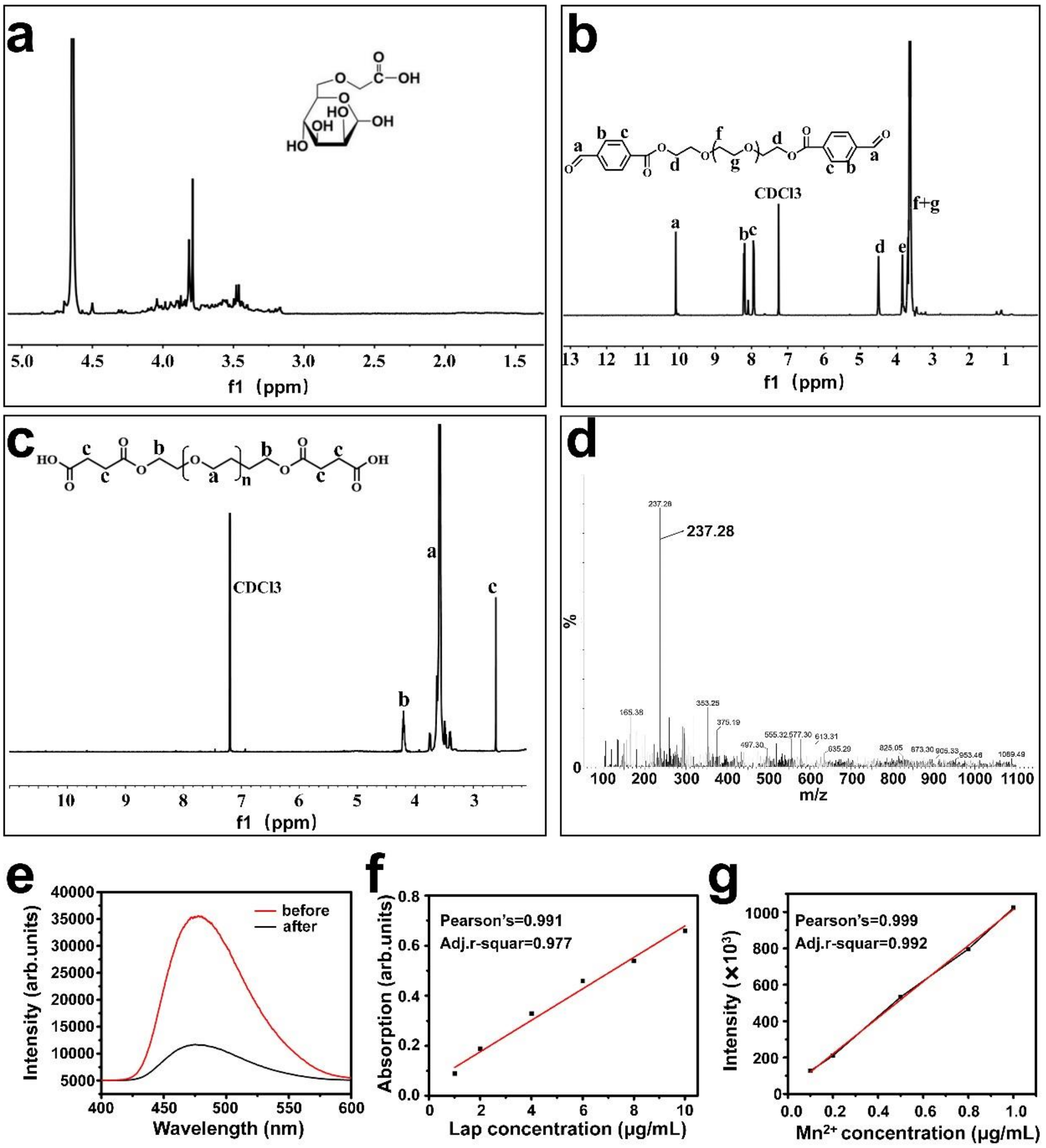

Supplementary Figure 1**.** Characterizations on the chemical structure of key ligands and drug release features. **(a-c)** 1H NMR spectra of Man-COOH, CHO-PEG$_{2000}$-CHO and COOH-PEG$_{2000}$-COOH. **(d)** Mass spectrum of Man-COOH. **(e)**: Fluorescamine assay on BSA and BSA-Man. **(f)** Standard curves of Mn^{2+} under different concentrations in ICP tests for profiling Mn^{2+} release. **(g)** Standard curve for the quantification of the Lap loading in Ft@Lap. Experiments in all panels were repeated three times independently with similar results. Source data are provided as a Source Data file.

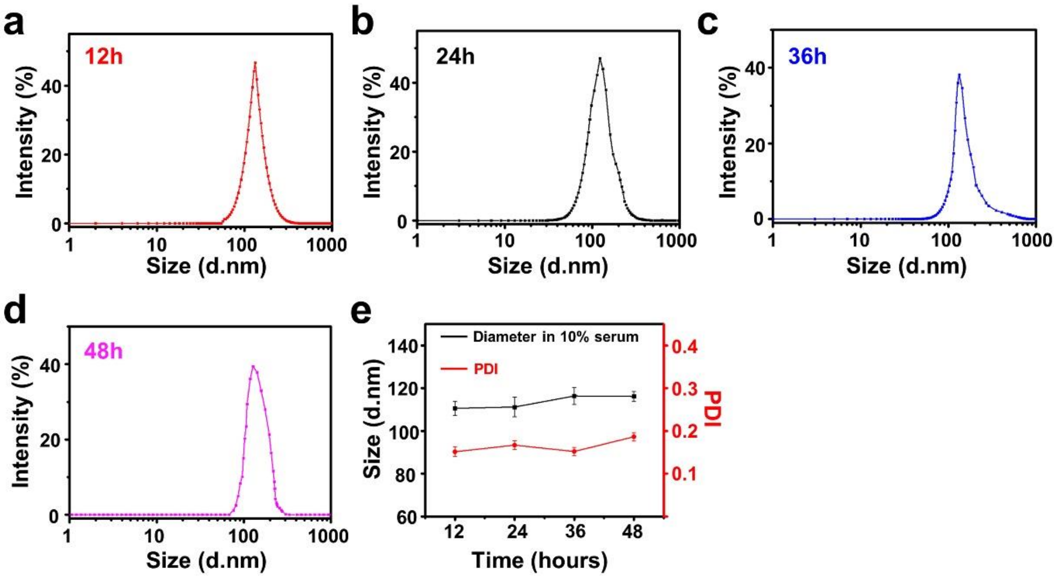

Supplementary Figure 2**.** Colloidal stability of the nanoagonist. **(a-d)** DLS analysis on the size changes of BSA-Man@Mn^{2+}-Ft@Lap in murine serum-supplemented PBS (10%) for (a) 12h, (b) 24h, (c) 36h and (d) 48h, respectively. Experiments in panel a-d were repeated three times independently with similar results. **(e)** Size and PDI changes of BSA-Man@Mn^{2+}-Ft@Lap in murine serum-supplemented PBS (10%). Data are presented as mean values ± SEM (n=3 independent experiments for panel e). Source data are provided as a Source Data file.

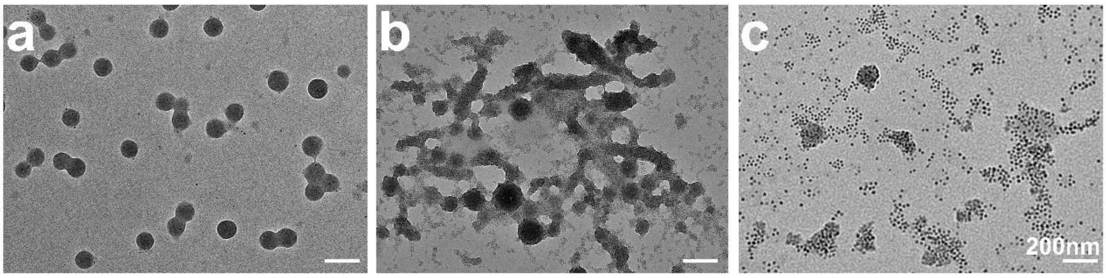

Supplementary Figure 3. Microscopic tests on the bioresponsive dissociation of the BSA-Man@Mn^{2+}-Ft@Lap nanoassembly. BSA-Man@Mn^{2+}-Ft@Lap was incubated at pH 6.5 for for **(a)** 0, **(b)** 6 and **(c)** 12h. Experiments in all panels were repeated three times independently with similar results.

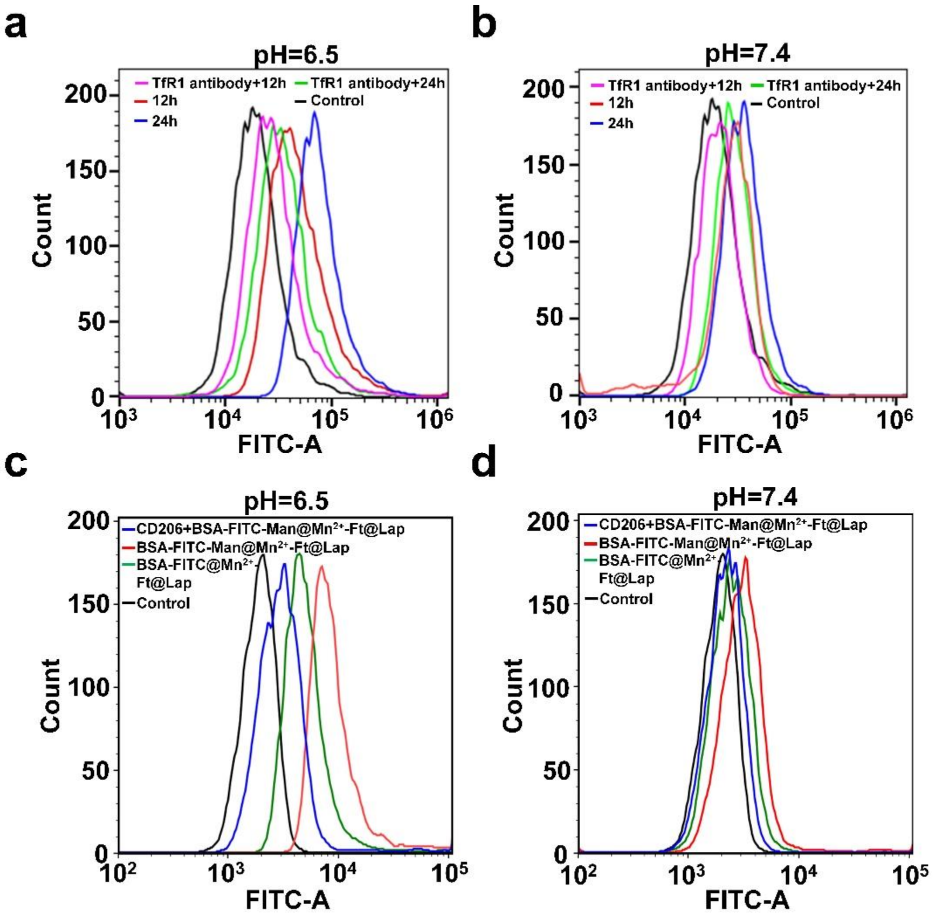

Supplementary Figure 4. Verification of the targeting mechanisms of the nanoagonist. **(a-b)** Flow cytometric analysis on the uptake of Ft contents by tumor cells under different conditions at pH 6.5 and pH 7.4. (**c-d**) Flow cytometric analysis on the uptake of mannose-modified BSA contents by DCs under different conditions at pH 6.5 and pH 7.4. Experiments in all panels were repeated three times independently with similar results.

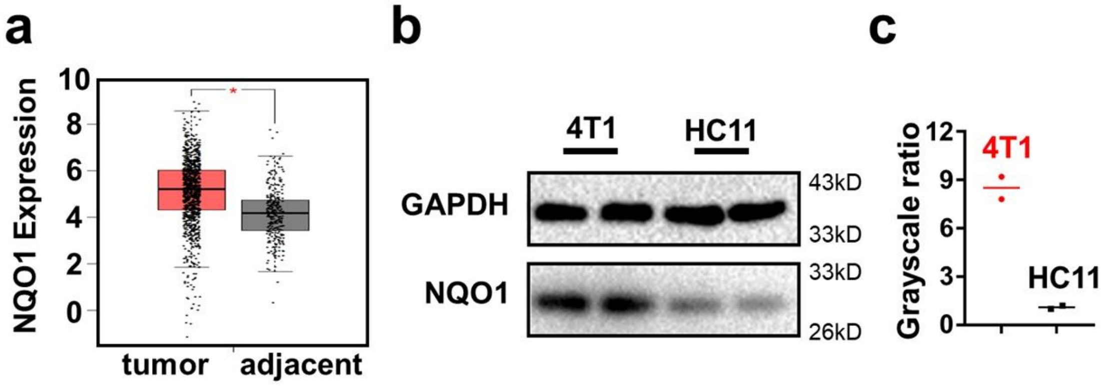

Supplementary Figure 5. The expression of NQO1 in tumor and normal cells. **(a)** The expression of NQO1 in RBCA and peri-tumor healthy tissues according to the TCGA and GTEx databases. Data are presented as mean values ± SEM (n=1085 biologically independent samples for tumor tissues and n=291 biologically independent samples for adjacent healthy tissues in panel a). Statistical analysis was carried out via Student's t-test. Center lines in box plots indicate median values. The box limits indicate the range of central 50% of the clinical data. Whiskers indicate 5 and 95 percentiles. Data points outside of the whisker plot are the outliers. * indicates significance at $p<0.05$. **(b/c)** NQO1 expression levels in 4T1 and HC11 cells according to the western blot analysis. Experiments in panel b and c were repeated three times independently with similar results. Data are presented as mean value (n=2 independent samples for panel c). Source data are provided as a Source Data file.

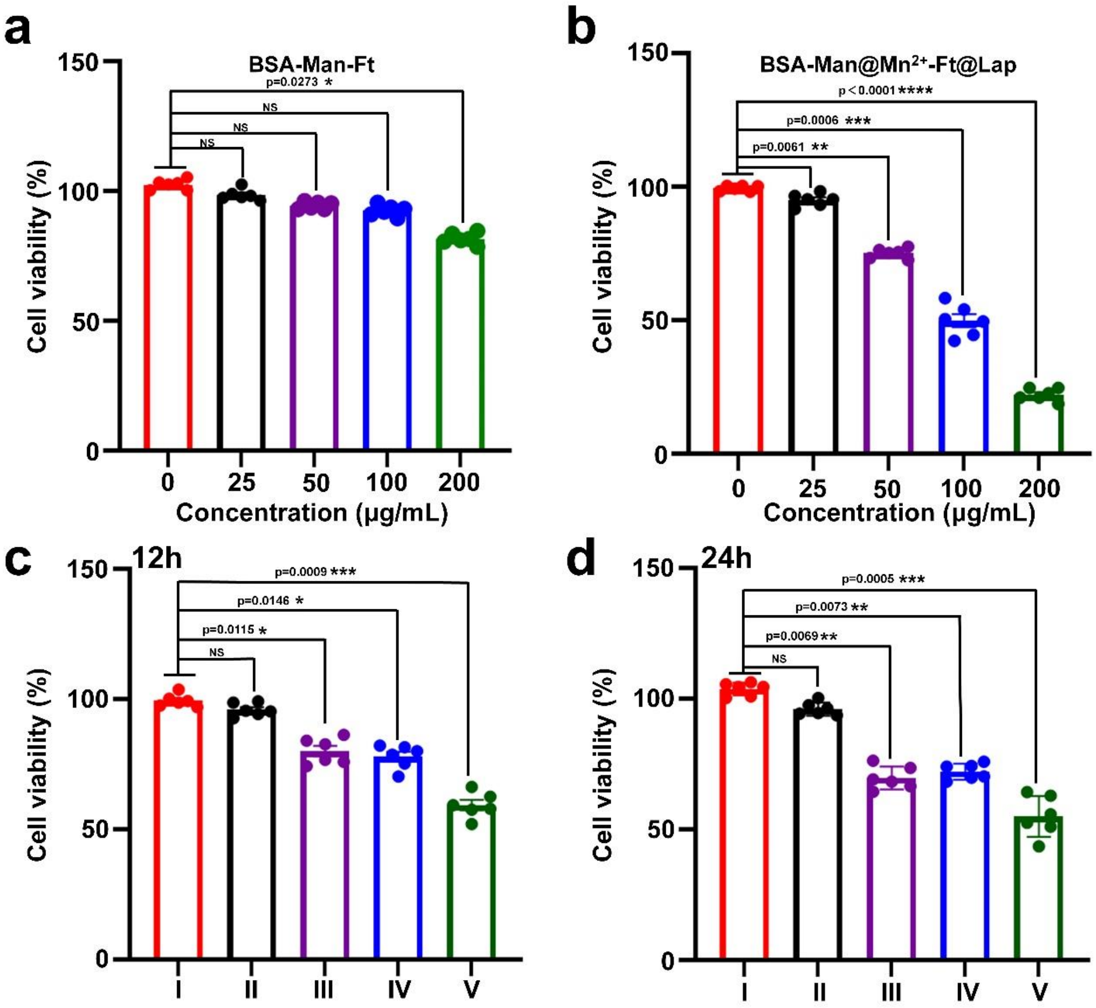

Supplementary Figure 6. Chemotherapeutic evaluation of the protein-based nanoassembly. **(a-b)** MTT assay of 4T1 cells after incubation with different concentrations of BSA-Man-Ft and BSA-Man@Mn^{2+}-Ft@Lap; **(c-d)** Survival rates of 4T1 cells measured via MTT assay after incubation with different samples for 12h and 24h.(I) control, (II) BSA-Man-Ft, (III) BSA-Man-Ft@Lap, (IV) BSA-Man@Mn^{2+}-Ft and (V) BSA-Man@Mn^{2+}-Ft@Lap. Data are presented as mean values ± SEM (n=6 biologically independent samples for panel a-d). Statistical analysis for all panels was carried out via one-way ANOVA method. * indicates significance at $p<0.05$, ** indicates significance at $p<0.01$, *** indicates significance at $p<0.001$, **** indicates significance at $p<0.0001$. Source data are provided as a Source Data file.

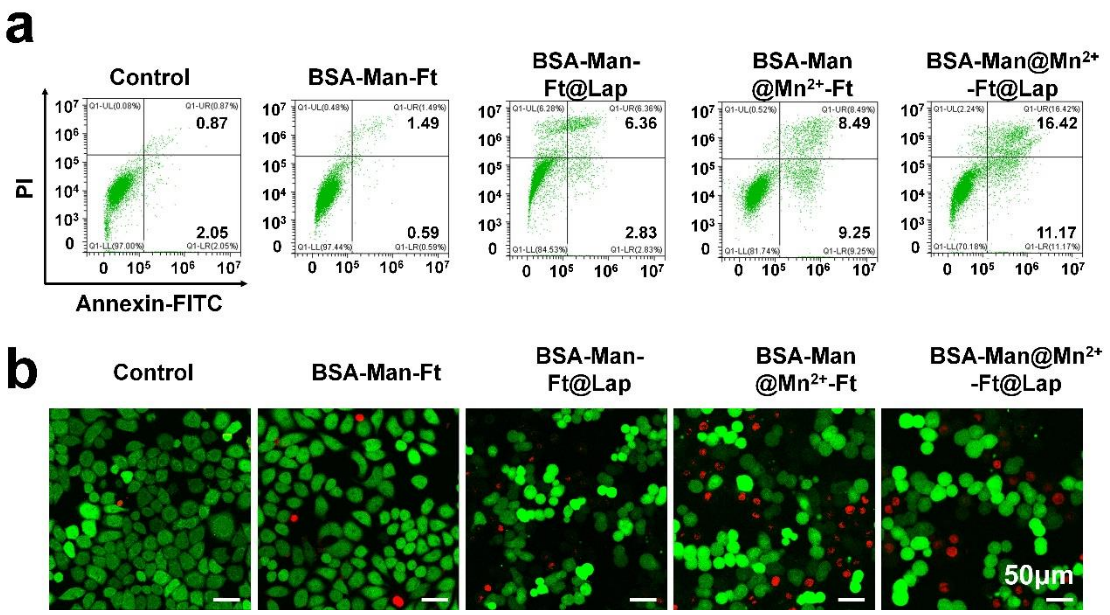

Supplementary Figure 7. Evaluation on the apoptosis inducing capability of the protein-based nanoassembly. **(a)** Flow cytometric analysis on the apoptosis levels of 4T1 cells in pH 6.5 under treatment with different samples. **(b)** Live/dead cell images of 4T1 cells after different treatment. Red fluorescence represents dead cells while the green color indicates living cells. Experiments in all panels were repeated three times independently with similar results.

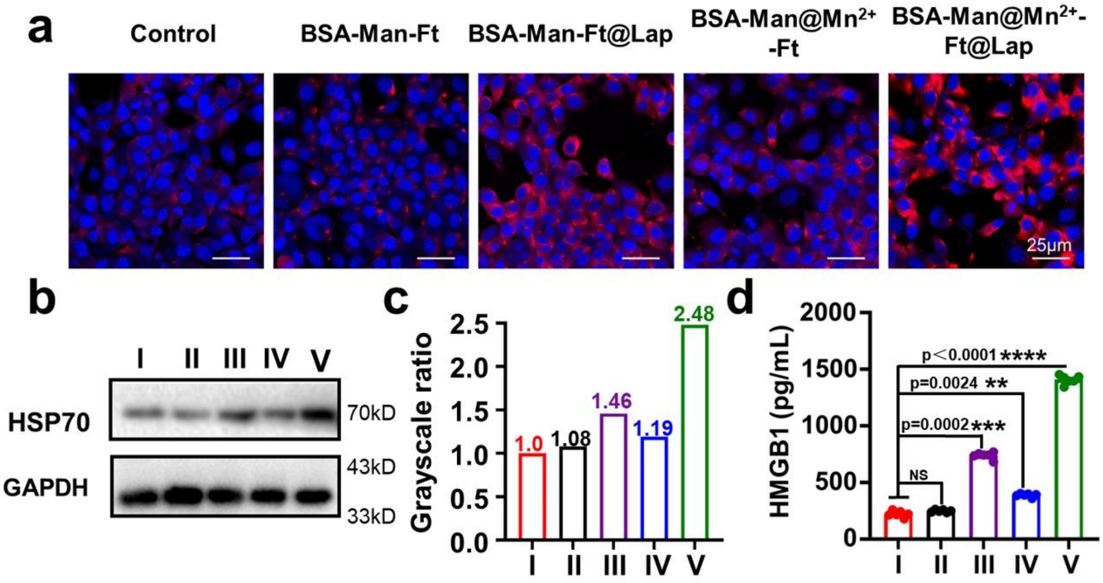

Supplementary Figure 8. Nanoagonist-induced ICD of tumor cells. **(a)** CLSM imaging of HSP70 expression after different treatments with (I) control, (II) BSA-Man-Ft, (III) BSA-Man-Ft@Lap, (IV) BSA-Man@Mn^{2+}-Ft and (V) BSA-Man@Mn^{2+}-Ft@Lap. Greater red fluorescence indicates higher expression levels. **(b)** Western blot analysis on the expression levels of HSP70 in 4T1 cells with different treatments. **(c)** The grayscale ratio of the HSP70 expression in panel b. Fluorescence and western blot experiments in panel a-c were repeated three times independently with similar results. **(d)** HMGB1 levels in cell culture supernatants after 24h of different treatments: (I) control, (II) BSA-Man-Ft, (III) BSA-Man-Ft@Lap, (IV) BSA-Man@Mn^{2+}-Ft and (V) BSA-Man@Mn^{2+}-Ft@Lap. Data are presented as mean values ± SEM (n=6 biologically independent samples for panel d). Statistical analysis for panel d was carried out via one-way ANOVA method. * indicates significance at $p<0.05$, ** indicates significance at $p<0.01$, *** indicates significance at $p<0.001$, **** indicates significance at $p<0.0001$. Source data are provided as a Source Data file.

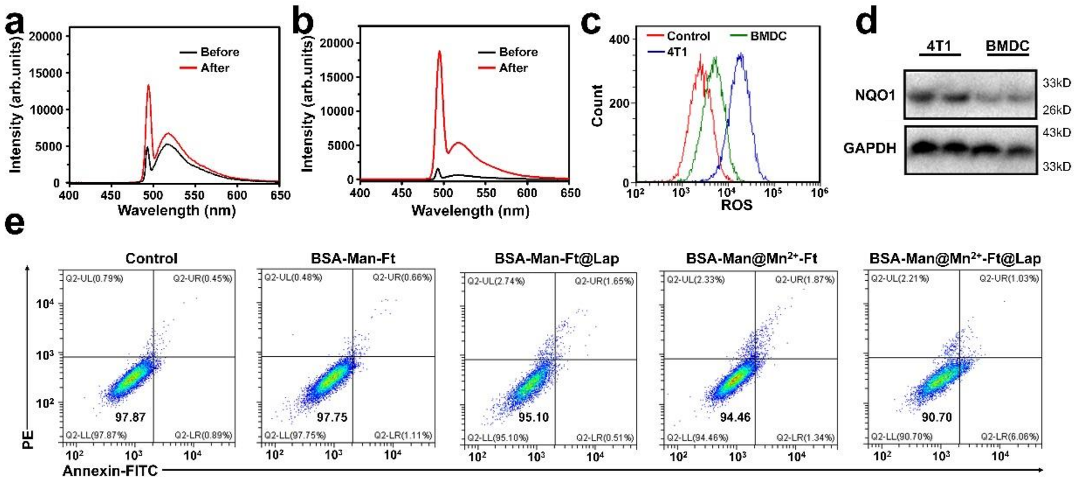

Supplementary Figure 9. Evaluations on the mechanism of the nanoagonist-mediated cGAS-STING activation. **(a)** FITC fluorescence retention in supernatant of BSA-FITC-Man@Mn^{2+}-Ft@Lap-incubated tumor cells under pH 6.5. **(b)** FITC fluorescence retention in supernatant of BSA-Man@Mn^{2+}-Ft-FITC@Lap-incubated tumor cells under pH 6.5. **(c)** Flow cytometric analysis on the ROS levels in tumor cells and DCs after treatment with BSA-Man@Mn^{2+}-Ft@Lap. **(d)** Western blot analysis on the different NQO1 expression levels in 4T1 tumor cells and BMDCs; **(e)** Flow cytometric analysis on the apoptosis levels of DCs after incubation with different samples at pH 6.5. Experiments in all panels were repeated three times independently with similar results. Source data are provided as a Source Data file.

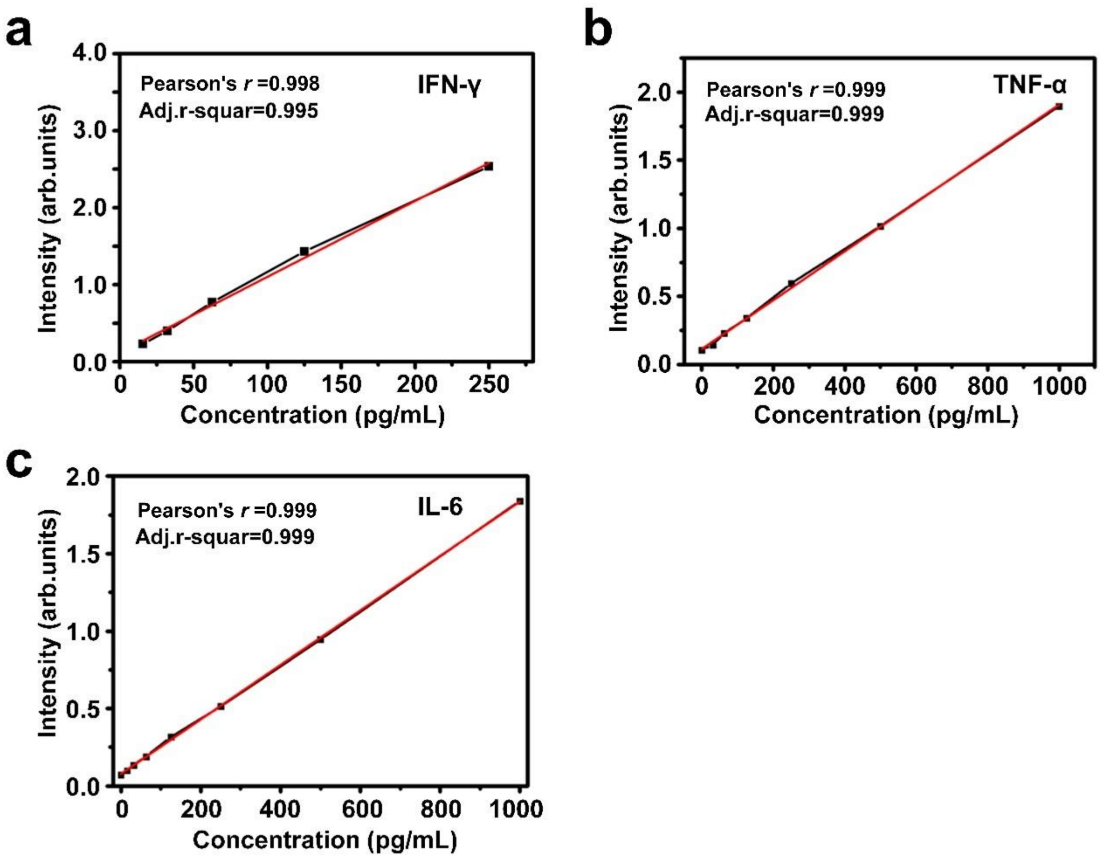

Supplementary Figure 10. Standard curves regarding the concentration-dependent changes in UV-vis absorption of IFN-γ, TNF-α and IL-6 by Elisa assay, which is then used for the quantitative analysis of cytokine section. Pearson's correlation coefficient *r* and goodness of fit were calculated using OriginPro 9.0. Experiments in all panels were repeated three times independently with similar results. Source data are provided as a Source Data file.

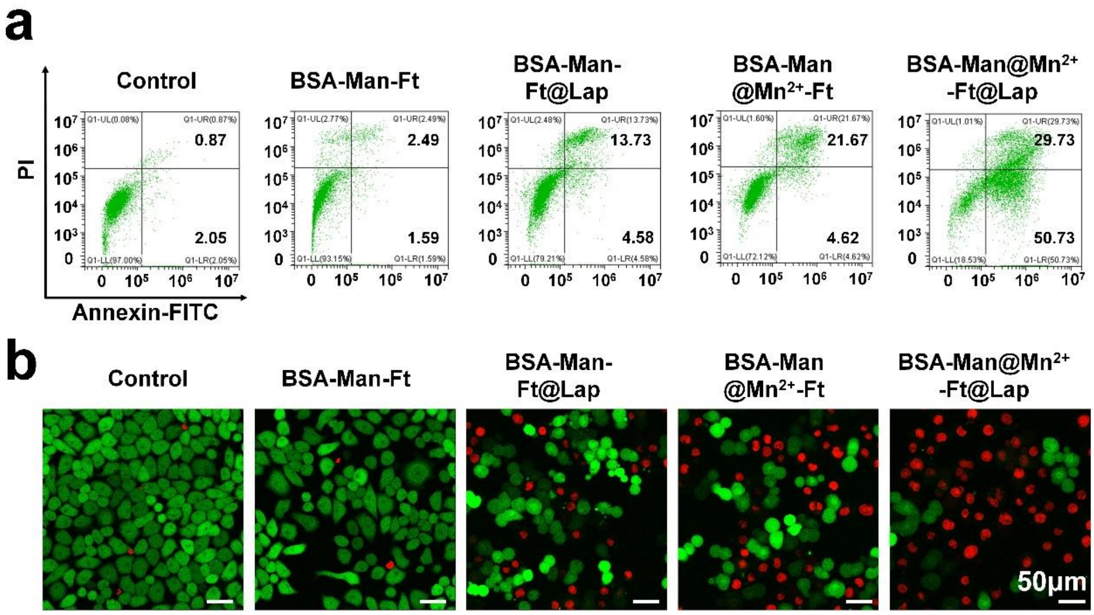

Supplementary Figure 11. Immunotherapeutic evaluation of the protein-based nanoassembly *in vitro*. **(a)** Flow cytometric analysis on the apoptosis levels of 4T1 cells at pH 6.5 under co-incubation with spleen cells and various other samples including control, BSA-Man-Ft, BSA-Man-Ft@Lap, BSA-Man@Mn^{2+}-Ft and BSA-Man@Mn^{2+}-Ft@Lap, respectively. **(b)** Live/dead cell images of 4T1 cells at pH 6.5 under co-incubation with splenic immune cells and various other samples including control, BSA-Man-Ft, BSA-Man-Ft@Lap, BSA-Man@Mn^{2+}-Ft and BSA-Man@Mn^{2+}-Ft@Lap, respectively. Red fluorescence represents dead cells while the green color represents living cells. Experiments in all panels were repeated three times independently with similar results.

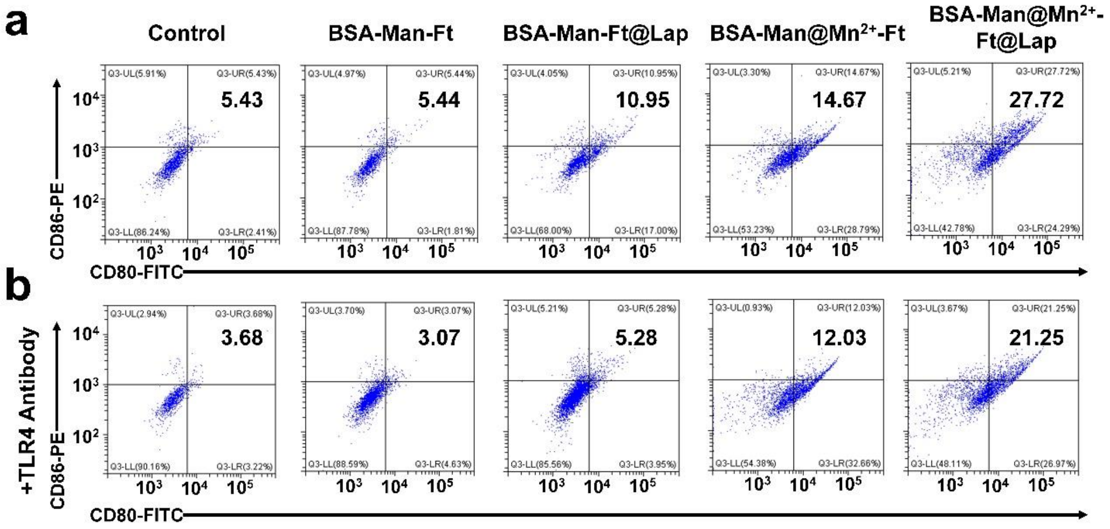

Supplementary Figure 12. Evaluation on potential contributing factors of DC maturation in vitro. **(a)** Flow cytometric analysis on the maturation status of DCs (CD80+/CD86+) after treatment with different samples. **(b)** Flow cytometric analysis on the maturation status of TLR4-antibody-pretreated DCs (CD80+/CD86+) after incubation with different samples. Flow cytometry experiments in all panels were repeated three times independently with similar results.

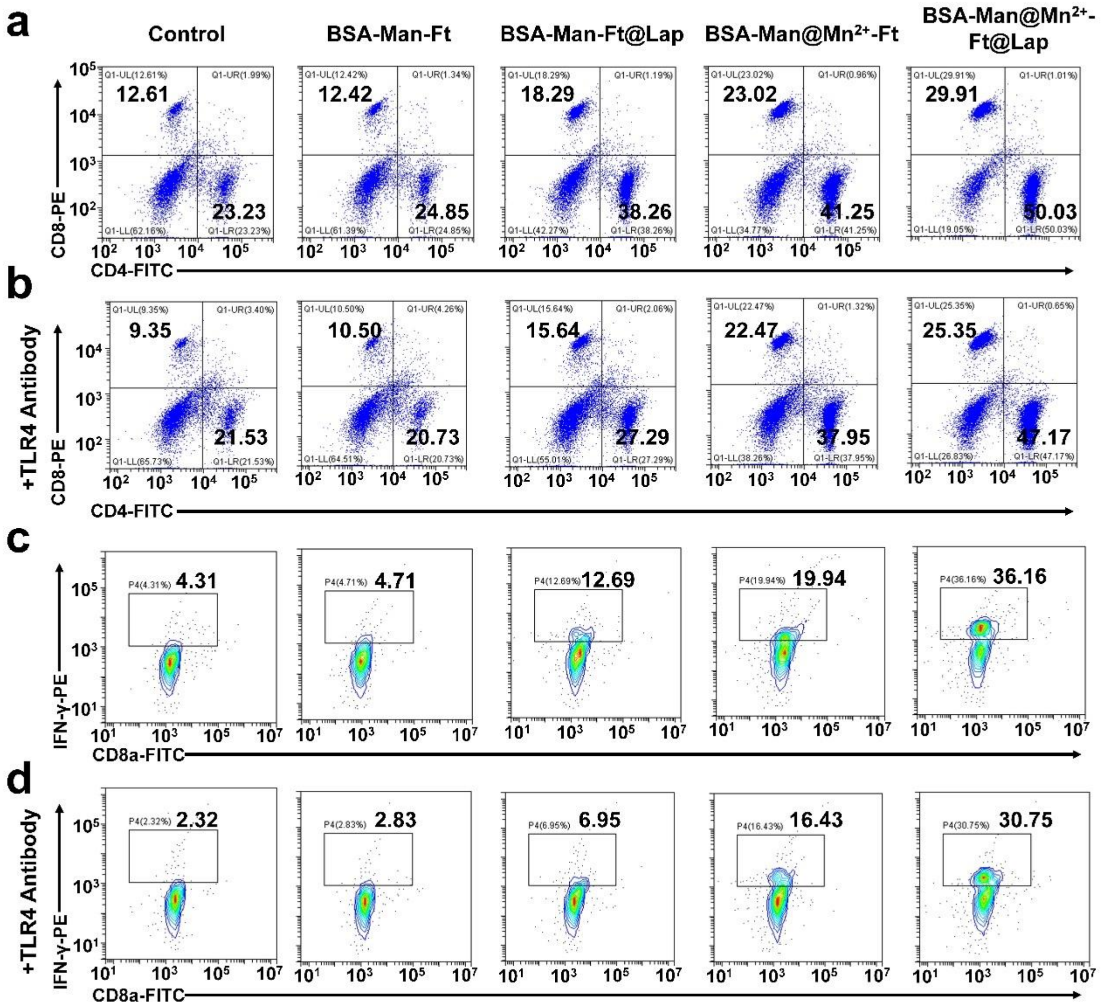

Supplementary Figure 13. Evaluations on the activation status of T cells in vitro. **(a)** Flow cytometric analysis on the ratio of activated T cells (CD4+/CD8+) in the tumor cell/splenic cell co-incubation system after treatment with different samples. **(b)** Flow cytometric analysis on the ratio of activated T cells (CD4+/CD8+) in the TLR4 antibody-pretreated tumor cell/splenic cell co-incubation system after treatment with samples. **(c)** Flow cytometric analysis on the ratio of IFN-γ–producing CD8+ T cells (CD8a+/IFN-γ+) in the tumor cell/splenic cell co-incubation system after treatment with different samples. **(d)** Flow cytometric analysis on the ratio of IFN-γ–producing CD8+ T cells (CD8a+/IFN-γ+) in the TLR4 antibody-pretreated tumor cell/splenic cell co-incubation system after treatment with different samples. Flow cytometry experiments in all panels were repeated three times independently with similar results.

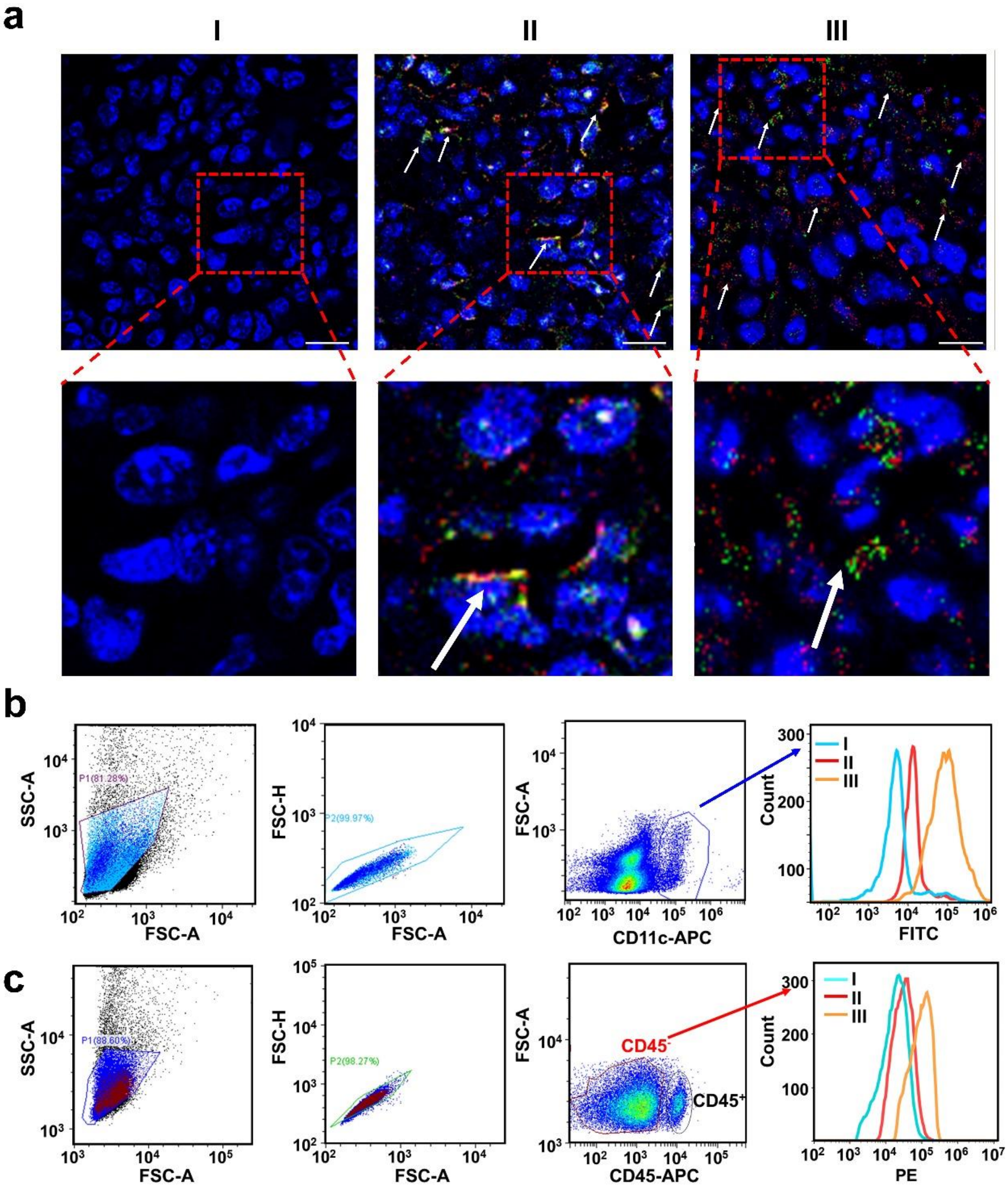

Supplementary Figure 14. Analysis of the targeting ability of the dissociable protein-based nanocomplex against different cell populations in TME. **(a)** Fluorescent imaging of tumor slices from mice after the intravenous injection of non-responsive amide-ligated BSA-Man@Mn^{2+}-FITC -Ft@Lap@RhB and tumor-responsive Schiff base-ligated BSA-Man@Mn^{2+}-FITC-Ft@Lap@RhB. The imaging experiments were repeated three times independently with similar results. **(b)** Gating strategy for analyzing the FITC fluorescence alteration in tumor-residing DCs via flow cytometry. This strategy was also applied for the

data in Figure 5, 7, 8, 9 and Supplementary Figure 12, 13, 21, 26, 27. **(c)** Gating strategy for analyzing the RhB fluorescence alteration in tumor cells via flow cytometry. The strategy was applied for the data in Supplementary Figure 14a. I: control, II: amide-ligated BSA-Man@Mn^{2+}-FITC -Ft@Lap@RhB, III: Schiff-based ligated BSA-Man@Mn^{2+}-FITC -Ft@Lap@RhB.

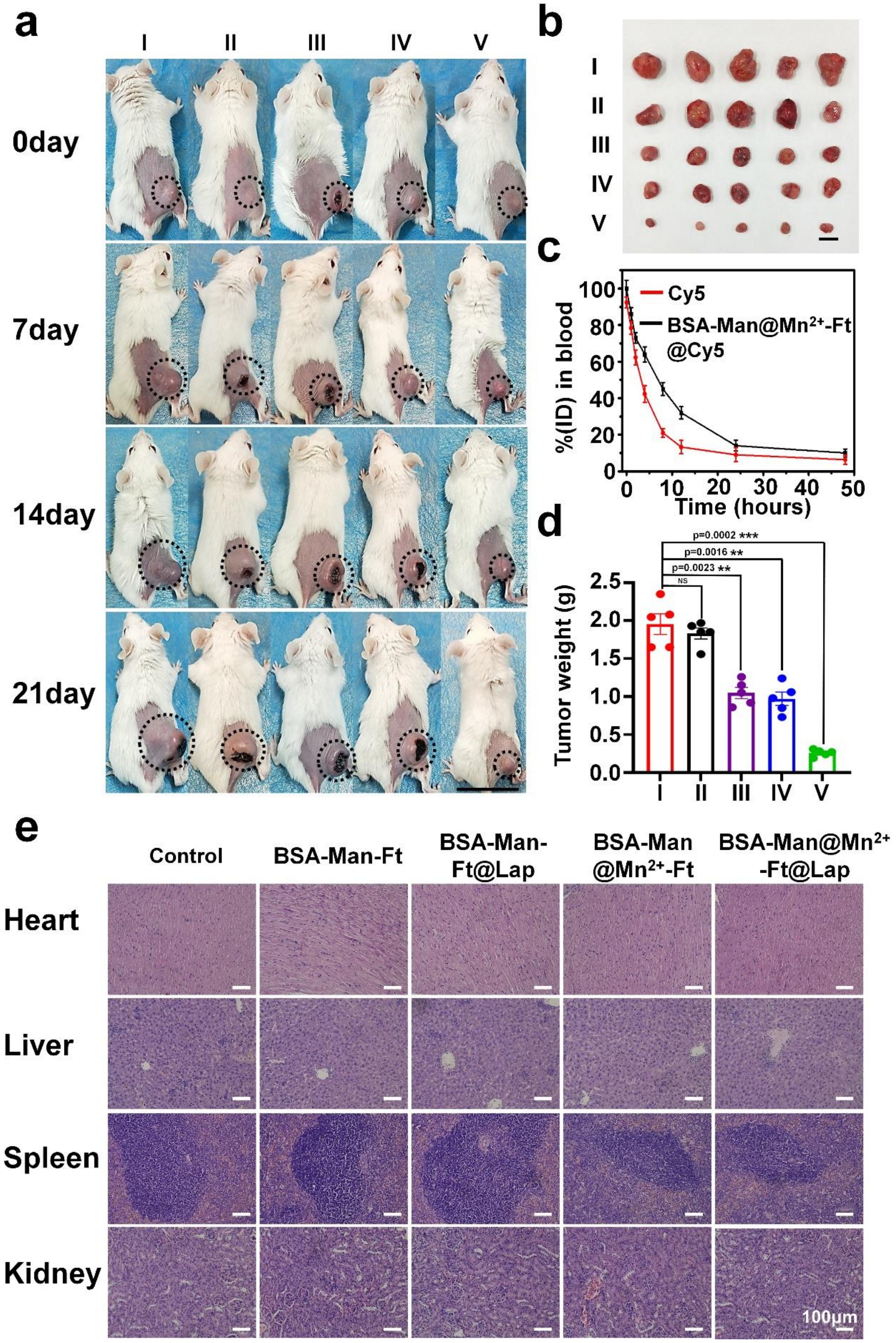

Supplementary Figure 15. Therapeutic evaluation of the protein-based nanoassembly *in vivo*. **(a)** Photographs of the tumor-bearing mice in different treatment groups (I) control, (II) BSA-Man-Ft, (III)

BSA-Man-Ft@Lap, (IV) BSA-Man@Mn^{2+}-Ft and (V) BSA-Man@Mn^{2+}-Ft@Lap. Scale bar=3cm. **(b)** Visual comparison of the extracted tumors after various treatments including (I) control, (II) BSA-Man-Ft, (III) BSA-Man-Ft@Lap, (IV) BSA-Man@Mn^{2+}-Ft and (V) BSA-Man@Mn^{2+}-Ft@Lap. Scale bar=1cm. **(c)** Blood half-life of the Cy5 labeled protein nanoassembly. **(d)** The average weight of tumors after different treatments. (I) control, (II) BSA-Man-Ft, (III) BSA-Man-Ft@Lap, (IV) BSA-Man@Mn^{2+}-Ft and (V) BSA-Man@Mn^{2+}-Ft@Lap. **(e)** H&E-stained tissue slices for the major organs and extracted tumors in different treatment groups. Scale bar=100μm. Experiments in panel e were repeated three times independently with similar results. Data are presented as mean values ± SEM (n=3 mice for panel c, n = 5 mice for panel d). Statistical analysis for panel d was carried out via one-way ANOVA method. * indicates significance at $p<0.05$, ** indicates significance at $p<0.01$, *** indicates significance at $p<0.001$. Source data are provided as a Source Data file.

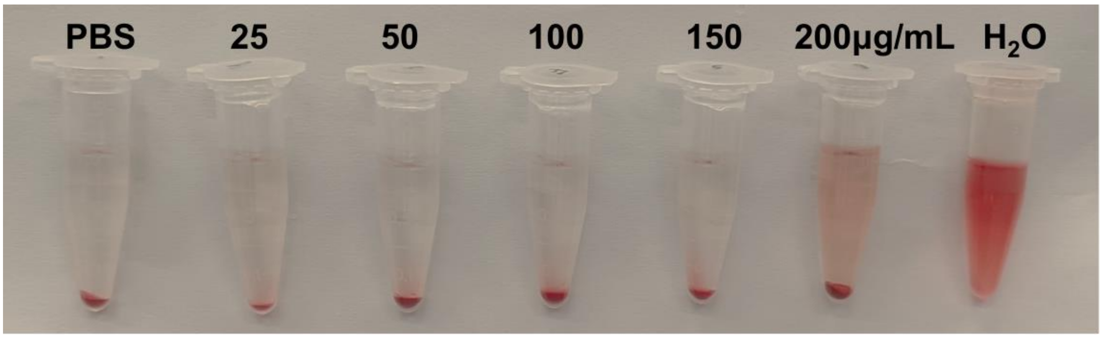

Samples	25μg/mL	50μg/mL	100μg/mL	150μg/mL	200μg/mL
Hemolysis (%)	0.3±0.2	0.4±0.2	0.6±0.4	0.7±0.3	2.1±0.2

Supplementary Figure 16. Suitability of the nanoagonist for intravenous administration. Hemolysis test of BSA-Man@Mn^{2+}-Ft@Lap nanoagonist under different concentrations. Experiments were repeated three times independently with similar results. Source data are provided as a Source Data file.

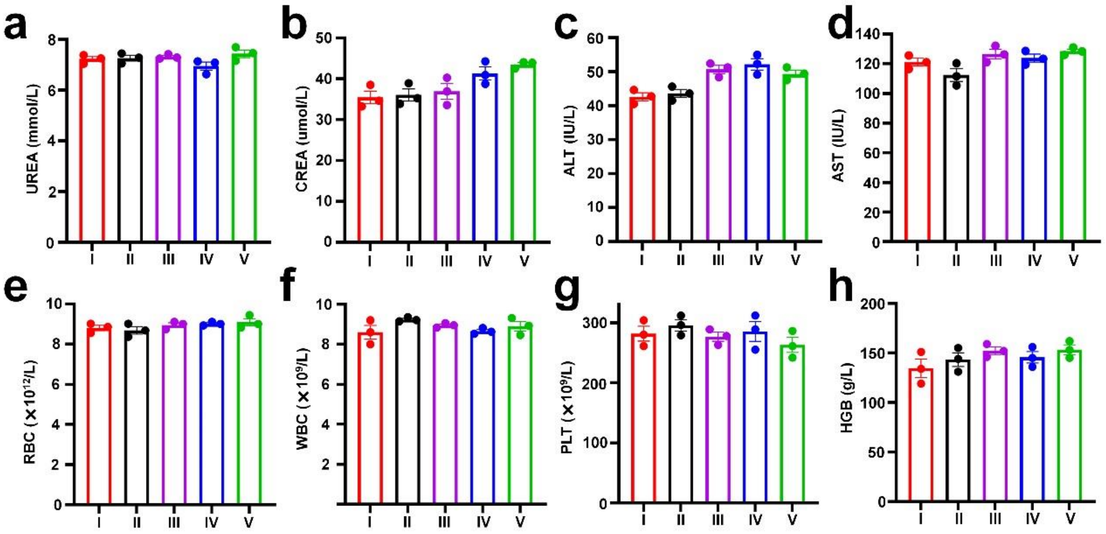

Supplementary Figure 17. Impact of the nanoagonist on liver and kidney functions in vivo as well as its hematological compatibility. Changes in key **(a/b)** kidney (creatinine and urea) and **(c/d)** liver (ALT and AST) indices as well as **(e-h)** vital hematological parameters of the mice after treatment with (I) control, (II) BSA-Man-Ft, (III) BSA-Man-Ft@Lap, (IV) BSA-Man@Mn^{2+}-Ft and (V) BSA-Man@Mn^{2+}-Ft@Lap *in vivo*. Data are presented as mean values ± SEM (n=3 mice for panel a-h). Source data are provided as a Source Data file.

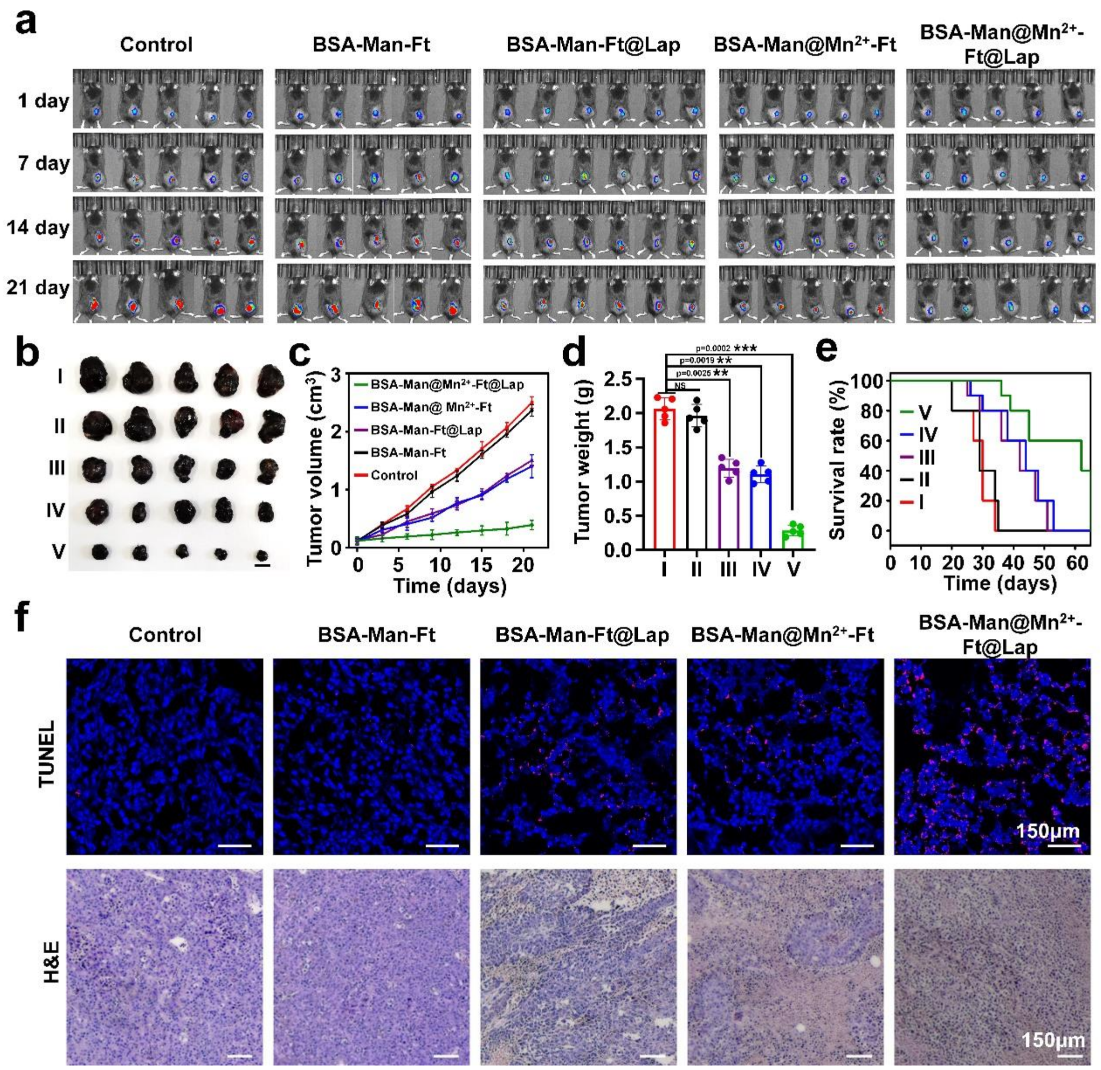

Supplementary Figure 18. BSA-Man@Mn^{2+}-Ft@Lap nanoassembly exhibits potent antitumor activity against B16F10-luc tumors in mice. **(a)** *In vivo* bioluminescence analysis of B16F10-luc tumor-bearing mice after different treatments. (I) control, (II) BSA-Man-Ft, (III) BSA-Man-Ft@Lap, (IV) BSA-Man@Mn^{2+}-Ft and (V) BSA-Man@Mn^{2+}-Ft@Lap. Scale bar=3cm; **(b)** Visual comparison of B16F10-luc tumors after various treatments including (I) control, (II) BSA-Man-Ft, (III) BSA-Man-Ft@Lap, (IV) BSA-Man@Mn^{2+}-Ft and (V) BSA-Man@Mn^{2+}-Ft@Lap. Scale bar=1cm; **(c)** Size changes of the B16F10-luc tumor during the incubation period after different treatment. **(d)** Weight changes of the B16F10-luc tumor after treatment with different samples. **(e)** Survival analysis of B16F10-luc tumor-bearing mice after treatment with (I) control, (II) BSA-Man-Ft, (III) BSA-Man-Ft@Lap, (IV) BSA-

Man@Mn^{2+}-Ft and (V) BSA-Man@Mn^{2+}-Ft@Lap. **(f)** H&E staining and TUNEL staining results of B16F10-luc tumor tissue samples after different treatment (I) control, (II) BSA-Man-Ft, (III) BSA-Man-Ft@Lap, (IV) BSA-Man@Mn^{2+}-Ft and (V) BSA-Man@Mn^{2+}-Ft@Lap. Experiments in panel f were repeated three times independently with similar results. Data are presented as mean values ± SEM (n=5 mice for panel c-d). Statistical analysis for panel d was carried out via one-way ANOVA method. * indicates significance at $p<0.05$, ** indicates significance at $p<0.01$, *** indicates significance at $p<0.001$. Source data are provided as a Source Data file.

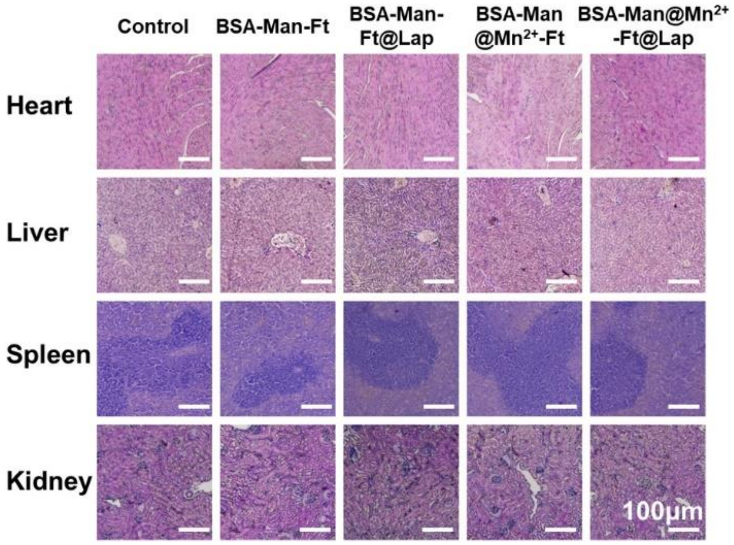

Supplementary Figure 19. Histocompatibility analysis of the nanoagonist on B16F10-luc tumor-bearing mice. H&E-stained tissue slices for the major organs in B16F10-luc tumor-bearing mice after different treatment. Scale bar=100μm. Experiments were repeated three times independently with similar results.

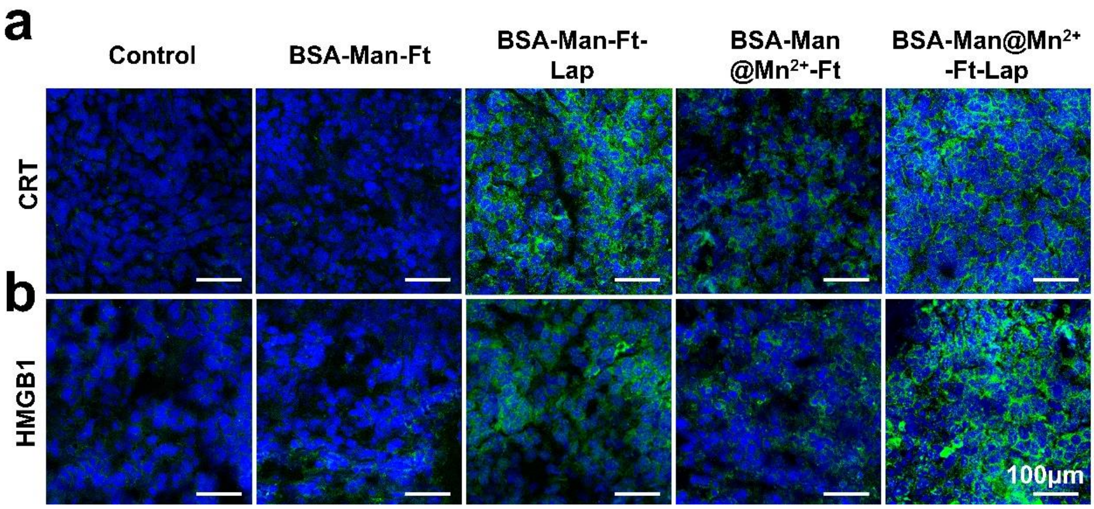

Supplementary Figure 20. Immunofluorescent analysis of the nanoassembly-induced DAMP release *in vivo*. **(a)** CRT and **(b)** HMGB1expression in 4T1 tumor slices after treatment with (I) control, (II) BSA-Man-Ft, (III) BSA-Man-Ft@Lap, (IV) BSA-Man@Mn^{2+}-Ft and (V) BSA-Man@Mn^{2+}-Ft@Lap. Scale bar=100μm. Experiments were repeated three times independently with similar results.

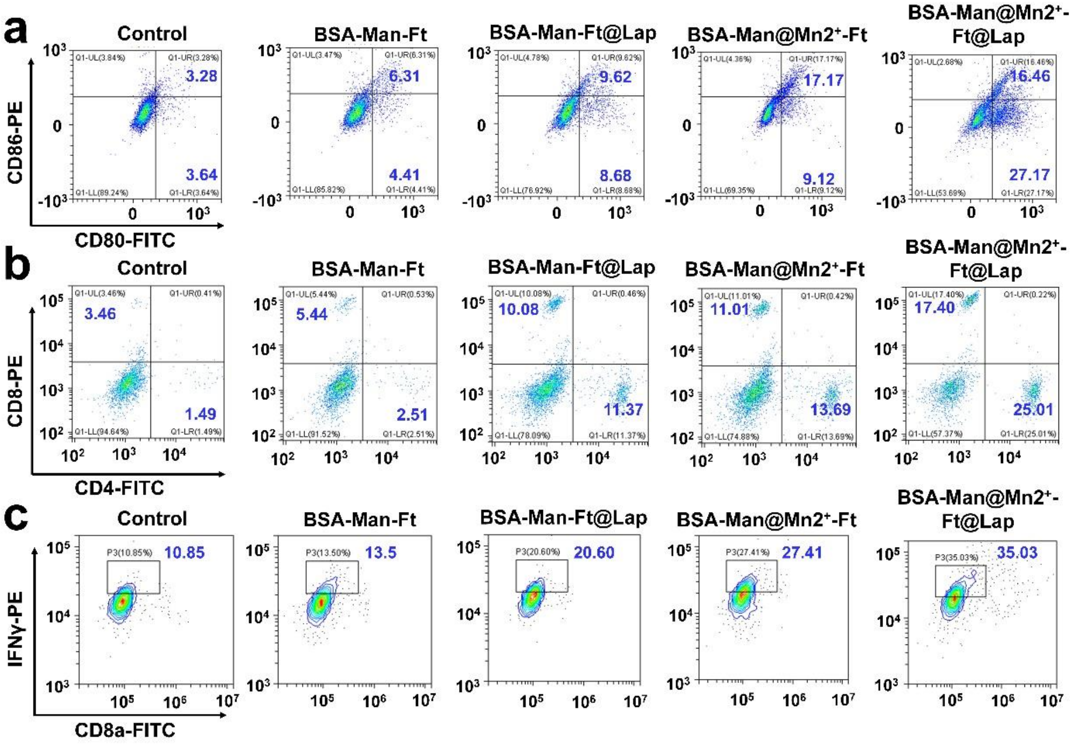

Supplementary Figure 21. *In vivo* analysis of the nanoassembly-mediated immunostimulation effect. **(a)** DC maturation ($CD80^+/CD86^+$), **(b)** T cell activation ($CD4^+/CD8^+$) and **(c)** relative ratio of IFN-γ-producing CD8+ T cells (CD8a/ IFN-γ) in the spleen of 4T1-luc tumor bearing mice were examined by flow cytometry after treatment with (I) control, (II) BSA-Man-Ft, (III) BSA-Man-Ft@Lap, (IV) BSA-Man@Mn^{2+}-Ft and (V) BSA-Man@Mn^{2+}-Ft@Lap. Experiments were repeated three times independently with similar results.

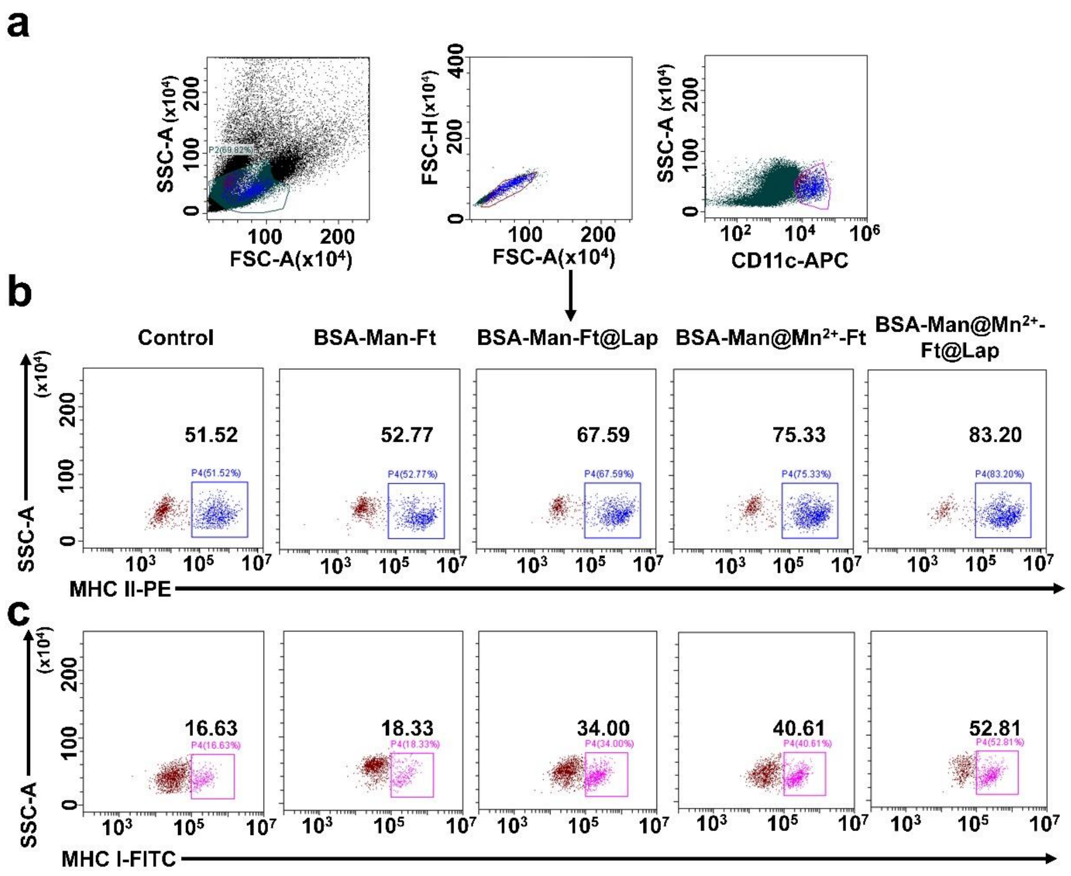

Supplementary Figure 22. Flow cytometric analysis on the nanoagonist-enhanced cross-presentation potential of mature DCs to activate CD4+ and CD8+ T cells. **(a)** Gating strategy for the determination of MHC I and MHC II expression levels on tumor-residing DCs. **(b)** Expression levels of MHC -II on tumor-residing DCs after various treatment. **(c)** Expression levels of MHC-I on tumor-residing DCs after various treatment. (I) control, (II) BSA-Man-Ft, (III) BSA-Man-Ft@Lap, (IV) BSA-Man@Mn^{2+}-Ft and (V) BSA-Man@Mn^{2+}-Ft@Lap. Flow cytometry experiments in panel b and c were repeated three times independently with similar results.

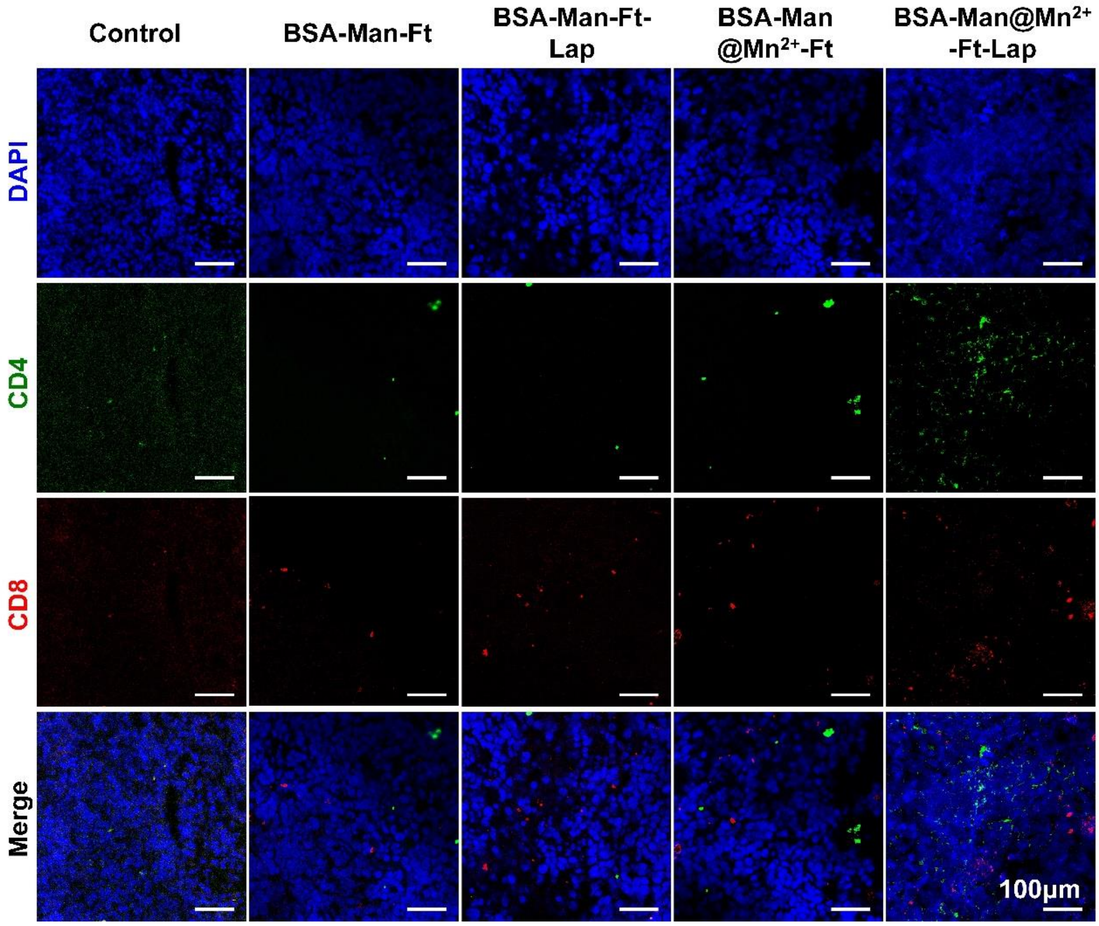

Supplementary Figure 23. Immunofluorescence analysis of the intratumoral infiltration of effector T cells after different treatment. Tumor tissues were harvested and co-stained with CD4-FITC and CD8-PE for the fluorescent tracking of activated T cells. Scale bar=100μm. Experiments were repeated three times independently with similar results.

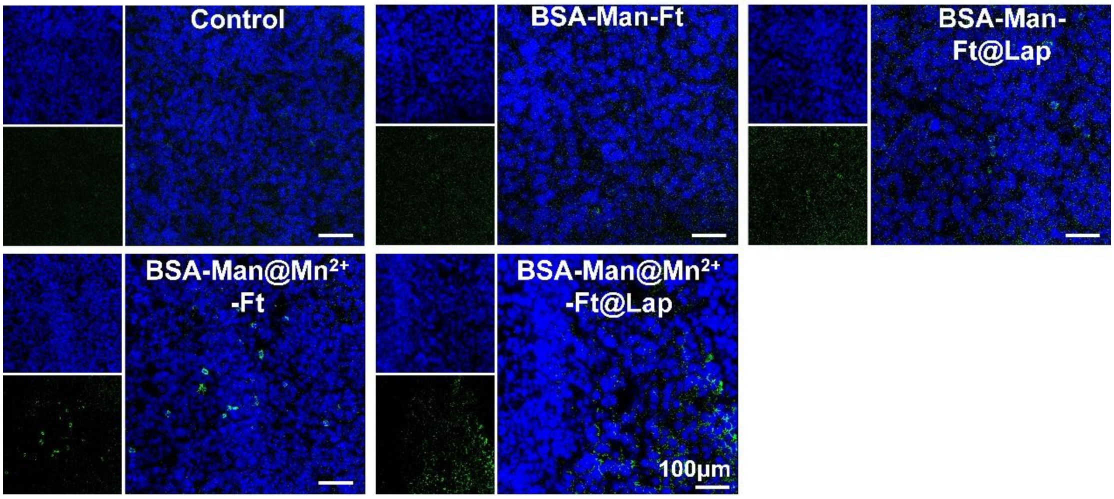

Supplementary Figure 24. Immunofluorescence analysis on the enhanced antitumor capacity of tumor-infiltrating T cells *in vivo*. Immunofluorescent image on the IFN-γ levels in tumor tissue sections from different groups. Experiments were repeated three times independently with similar results.

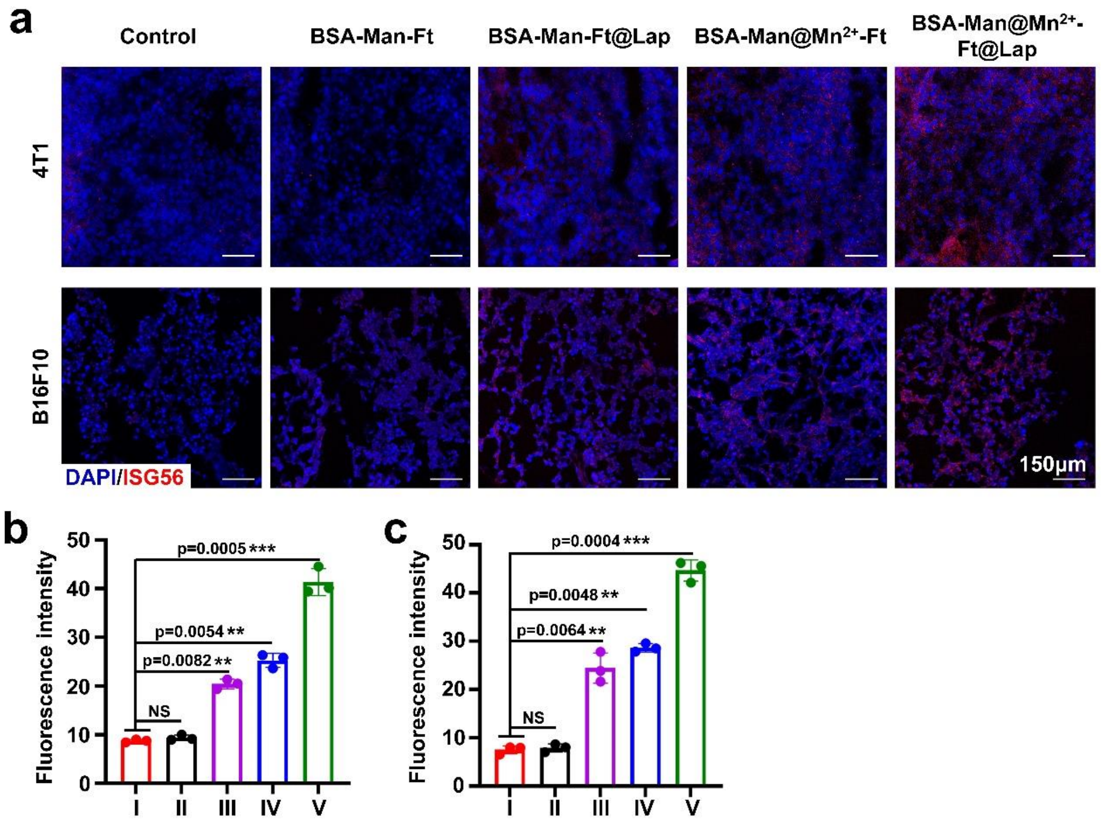

Supplementary Figure 25. Immunofluorescence analysis on intratumoral expression of ISG after different treatment. **(a)** Immunofluorescence imaging of ISG in 4T1/B16F10 tumor tissue sections after various treatments including (I) control, (II) BSA-Man-Ft, (III) BSA-Man-Ft@Lap, (IV) BSA-Man@Mn^{2+}-Ft and (V) BSA-Man@Mn^{2+}-Ft@Lap. (b/c): Quantitative analysis of fluorescence intensity in **(b)** 4T1 and **(c)** B16F10 tumors from panel a. Scale bar:150μm. Immunofluorescence imaging experiments in panel a were repeated three times independently with similar results. Data are presented as mean values ± SEM (n=3 mice for panel b-c). Statistical analysis for panel b and c was carried out via one-way ANOVA method. * indicates significance at $p<0.05$, ** indicates significance at $p<0.01$, *** indicates significance at $p<0.001$. Source data are provided as a Source Data file.

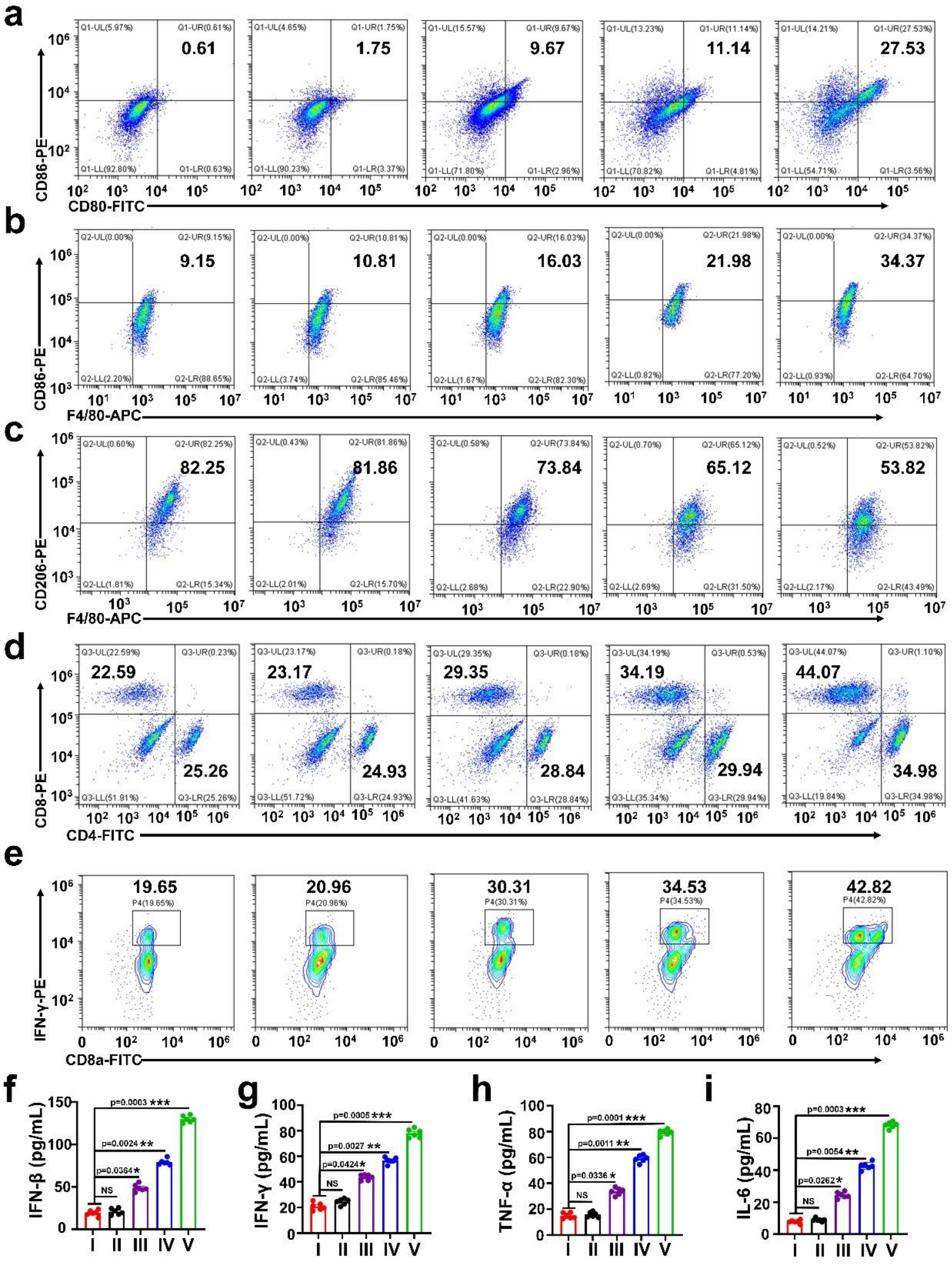

Supplementary Figure 26. BSA-Man@Mn^{2+}-Ft@Lap nanoassembly elicits robust antitumor immunity in B16F10 tumors. **(a-e)** Flow cytometric analysis on DC maturation (CD80^{+}/CD86^{+}), M1/M2 macrophage polarization state (F4/80/CD86^{+}/CD206^{+}) and T cell activation (CD4+/CD8+, CD8a/IFN-γ) in B16F10 tumor tissues after treatment with (I) control, (II) BSA-Man-Ft, (III) BSA-Man-Ft@Lap, (IV) BSA-Man@Mn^{2+}-Ft and (V) BSA-Man@Mn^{2+}-Ft@Lap in B16F10-tumor. **(f-i)** Serum levels of

IFN-β, IFN-γ, TNF-α and IL-6 in B16F10 tumor-bearing mice after different treatments. Flow cytometry experiments in panel a-e were repeated three times independently with similar results. Data are presented as mean values ± SEM (n=6 mice for panel f-i). Statistical analysis for panel f-i was carried out via one-way ANOVA method. * indicates significance at $p<0.05$, ** indicates significance at $p<0.01$, *** indicates significance at $p<0.001$. Source data are provided as a Source Data file.

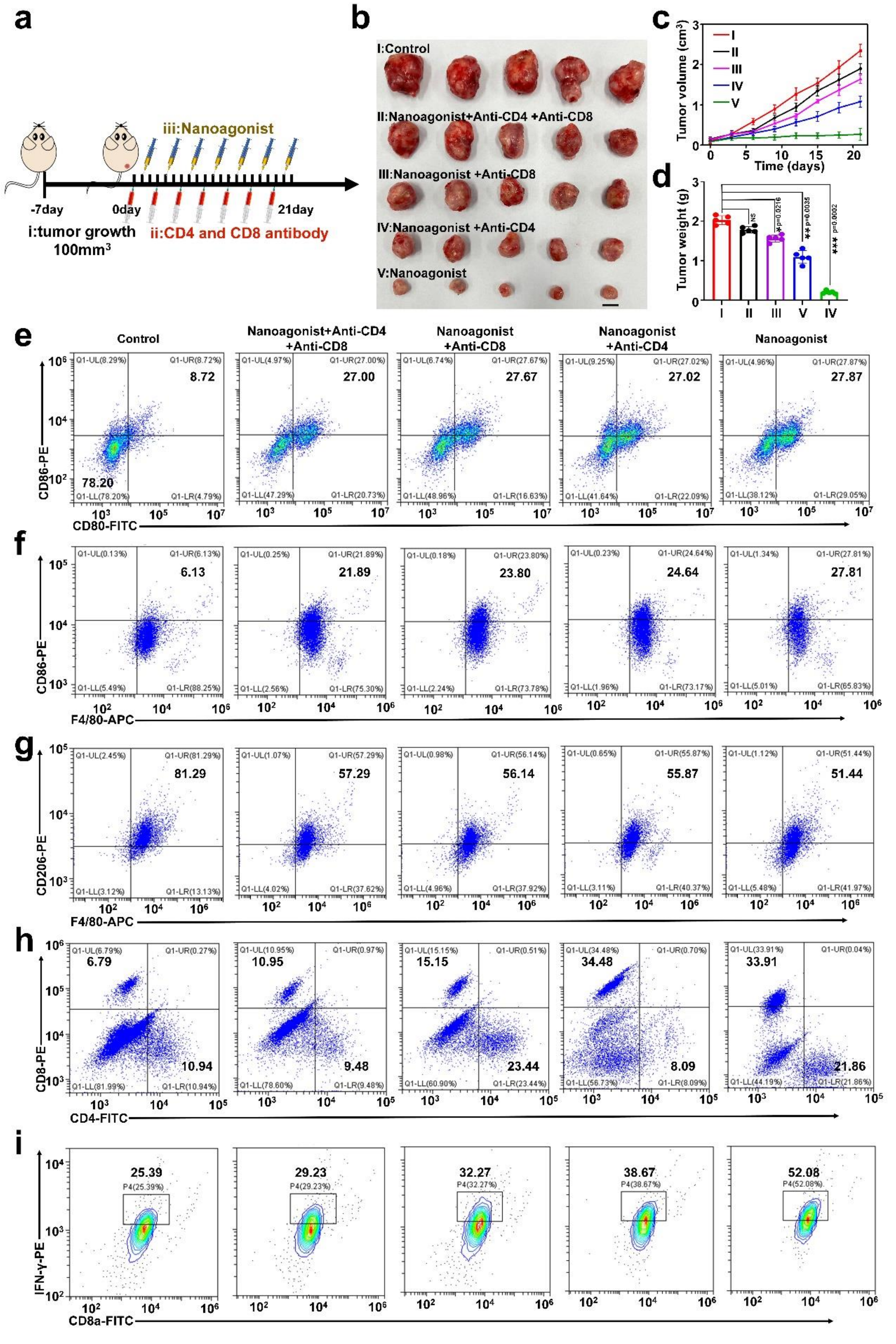

Supplementary Figure 27. CD4+ and CD8+ T cells have indispensable roles in the nanoagonist-enabled antitumor activity. **(a)** Schematic diagram of the CD4/CD8 T cell depletion treatment in 4T1-luc tumor-

bearing Balb-c mice receiving nanoagonist-mediated immunotherapy. **(b)** Visual comparison of the extracted tumors after various treatments. Scale bar=1cm; **(c)** Volume changes of 4T1-luc tumors throughout the treatment period; **(d)** Final tumor weight after treatment with different samples; **(e-i)** Flow cytometric analysis on DC maturation (CD80+/CD86+), M2-to-M1 macrophage repolarization (F4/80/CD86+ and F4/80/CD206+) and T cell activation status (CD4+/CD8+, CD8a/IFN-γ) in tumor tissues after various treatment. (I) control, (II) BSA-Man@Mn^{2+}-Ft@Lap+antiCD4+antiCD8, (III) BSA-Man@Mn^{2+}-Ft@Lap+antiCD4, (IV) BSA-Man@Mn^{2+}-Ft@Lap+antiCD8 and (V) BSA-Man@Mn2+-Ft@Lap in vivo. Flow cytometry experiments in panel e-i were repeated three times independently with similar results. Data are presented as mean values ± SEM (n=5 mice for panel c-d). Statistical analysis for panel d was carried out via one-way ANOVA method. * indicates significance at $p<0.05$, ** indicates significance at $p<0.01$, *** indicates significance at $p<0.001$. Source data are provided as a Source Data file.

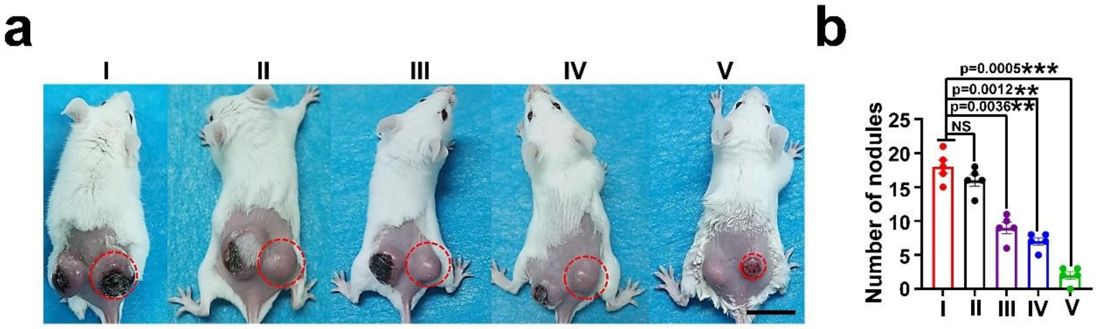

Supplementary Figure 28. BSA-Man@Mn^{2+}-Ft@Lap nanoassembly elicits systemic immunity for whole body tumor inhibition. **(a)** Photographs of mice bearing bilateral tumors throughout the treatment course with (I) control, (II) BSA-Man-Ft, (III) BSA-Man-Ft@Lap, (IV) BSA-Man@Mn^{2+}-Ft and (V) BSA-Man@Mn^{2+}-Ft@Lap. Scale bar=2cm; **(b)** Quantitative analysis of lung metastasis nodules after various treatments including (I) control, (II) BSA-Man-Ft, (III) BSA-Man-Ft@Lap, (IV) BSA-Man@Mn^{2+}-Ft and (V) BSA-Man@Mn^{2+}-Ft@Lap. Data are presented as mean values ± SEM (n=5 mice). Statistical analysis was carried out via one-way ANOVA method (* $p< 0.05$, ** $p< 0.01$ and *** $p< 0.001$). Source data are provided as a Source Data file.

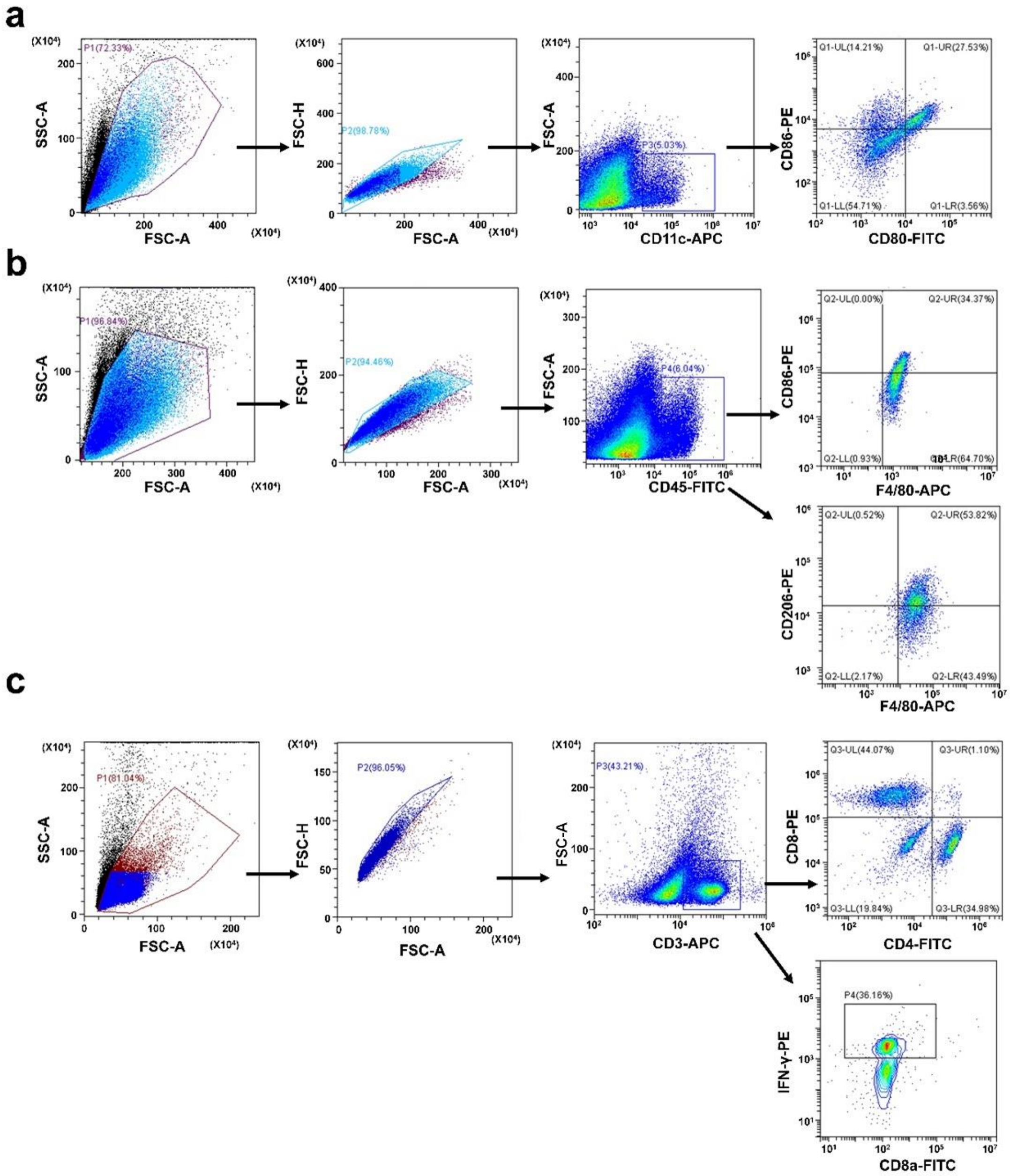

Supplementary Figure 29. Gating strategy for the FACS tests. Panel a-c are the gating strategies for isolating (a) CD80+/CD86+ mature DCs, (b) polarized macrophages (M1/M2), (c) CD4+/CD8+ and CD8+/IFN-γ+ T cells. This loop-gate strategy was applied for analysing the data shown in Figure 5, 7, 8, 9 and Supplementary Figure 12, 13, 21, 26, 27.

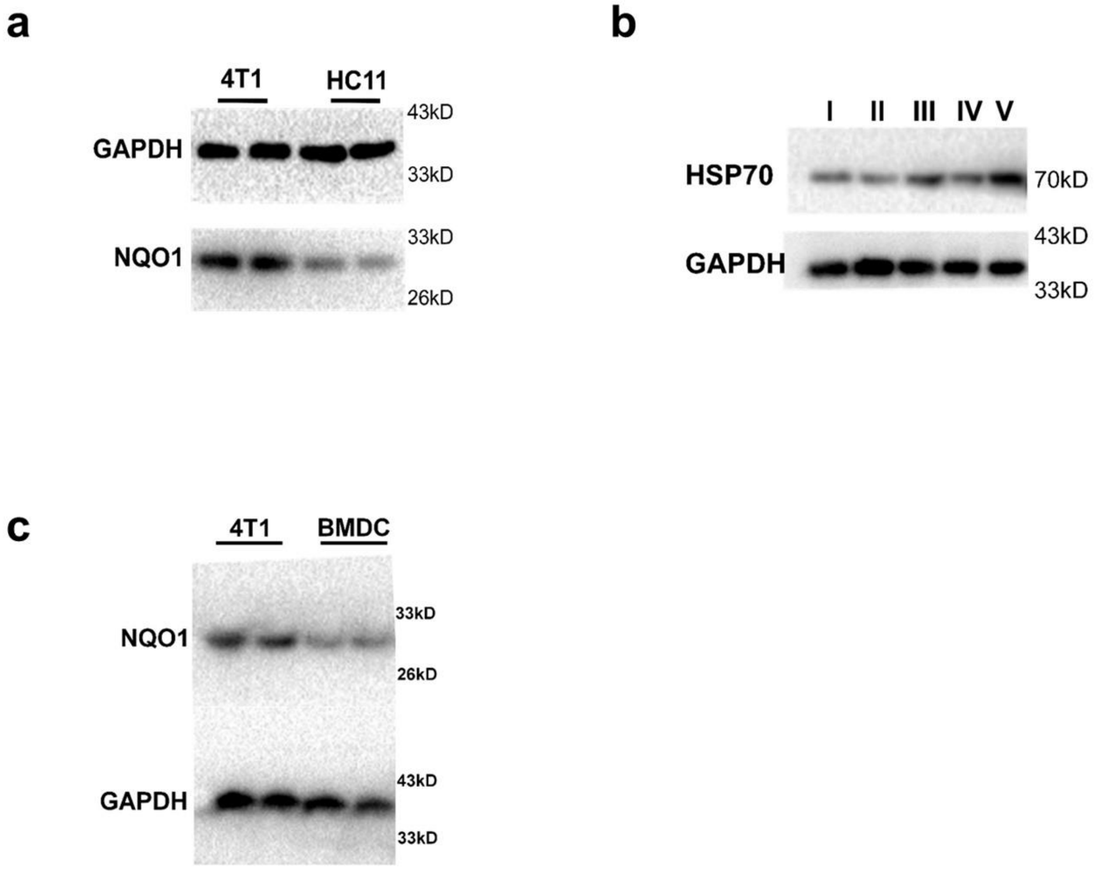

Supplementary Figure 30. Uncropped scans of western blot with molecular weight markers. (a) Related results for Supplementary Fig. 5b. (b) Related results for Supplementary Fig. 8b. (c) Related results for Supplementary Fig. 9d.

Supplementary Tables

Supplementary Table 1. **Validation information for all antibodies used in this study.** All information is provided by the manufactures and could be found on their website.

Antibody name	Validation information
**GADPH**	https://www.ptgcn.com/products/GAPDH-Antibody-10494-1-AP.htm
**BAX**	https://www.ptgcn.com/products/BAX-Antibody-50599-2-Ig.htm
**Bcl-2**	https://www.ptgcn.com/products/Bcl2-Antibody-26593-1-AP.htm
**HMGB1**	https://www.ptgcn.com/products/HMGB1-Antibody-66525-1-Ig.htm
**CRT**	https://www.ptgcn.com/products/CALR-Antibody-10292-1-AP.htm
**HSP70**	https://www.ptgcn.com/products/HSP70-Antibody-66183-1-Ig.htm
**NQO1**	https://www.ptgcn.com/products/NQO1-Antibody-11451-1-AP.htm
**HRP-conjugated anti-rabbit secondary antibody**	https://www.ptgcn.com/products/HRP-conjugated-Affinipure-Goat-Anti-Rabbit-IgG-H-L-secondary-antibody.htm
**Cy3-conjugated Goat anti-Rabbit IgG**	https://www.sangon.com/productDetail?productInfo.code=D110062
**Alexa Fluor 488-conjugated Goat anti-rabbit IgG**	https://www.sangon.com/productDetail?productInfo.code=D110061
**STING**	https://www.cellsignal.cn/products/primary-antibodies/sting-d2p2f-rabbit-mab/13647?site-search-type=Products&N=4294956287&Ntt=sting&fromPage=plp
**p-STING**	https://www.cellsignal.cn/products/primary-antibodies/phospho-sting-ser366-e9a9k-rabbit-mab/50907?site-search-type=Products&N=4294956287&Ntt=sting&fromPage=plp
**TBK1**	https://www.cellsignal.cn/products/primary-antibodies/tbk1-nak-d1b4-rabbit-mab/3504?site-search-type=Products&N=4294956287&Ntt=tbk1&fromPage=plp
**p-TBK1**	https://www.cellsignal.cn/products/primary-antibodies/phospho-tbk1-nak-ser172-d52c2-xp-rabbit-mab/5483?site-search-type=Products&N=4294956287&Ntt=p-tbk1&fromPage=plp
**p-IRF3**	https://www.cellsignal.cn/products/primary-antibodies/phospho-irf-3-ser396-d6o1m-rabbit-mab/29047?site-search-type=Products&N=4294956287&Ntt=irf3&fromPage=plp
**CD4**	https://www.leinco.com/p/anti-mouse-cd4-clone-gk1-5-purified-functional-grade-gold/
**CD8**	https://www.leinco.com/p/anti-mouse-cd8a-ly-2-purified-functional-grade-gold/

**FITC anti-mouse IFN-γ Antibody**	https://www.biolegend.com/en-us/products/fitc-anti-mouse-ifn-gamma-antibody-995
**FITC-antiCD80**	https://www.biolegend.com/en-us/products/fitc-anti-mouse-cd80-antibody-41
**PE-antiCD86**	https://www.biolegend.com/en-us/products/pe-anti-mouse-cd86-antibody-18945
**APC-antiF4/80**	https://www.biolegend.com/en-us/products/apc-anti-mouse-f4-80-recombinant-antibody-18756
**FITC-antiCD4**	https://www.biolegend.com/en-us/products/fitc-anti-mouse-cd4-antibody-248
**PE-antiCD8**	https://www.biolegend.com/en-us/products/pe-anti-mouse-cd8a-antibody-20990
**FITC-antiCD8**	https://www.biolegend.com/en-us/products/fitc-anti-mouse-cd8a-antibody-153
**FITC-antiIFN-γ**	https://www.biolegend.com/en-us/products/fitc-anti-mouse-ifn-gamma-antibody-995
**APC-antiCD11c**	https://www.biolegend.com/en-us/products/apc-anti-mouse-cd11c-antibody-1813
