# Supplementary material for: A protein-based cGAS-STING nanoagonist enhances T cell-mediated anti-tumor immune responses
Source: Nat Commun. 2022 Sep 28;13:5685. doi: 10.1038/s41467-022-33301-0 (PMC9515186; doi:10.1038/s41467-022-33301-0)
Supplement: Supplementary file 2 — Reporting Summary [file 41467_2022_33301_MOESM2_ESM.pdf]

nature portfolio

Corresponding author(s): Menghuan Li, Zhong Luo

Last updated by author(s): Aug 26, 2022

Reporting Summary

Nature Portfolio wishes to improve the reproducibility of the work that we publish. This form provides structure for consistency and transparency in reporting. For further information on Nature Portfolio policies, see our Editorial Policies and the Editorial Policy Checklist.

Statistics

For all statistical analyses, confirm that the following items are present in the figure legend, table legend, main text, or Methods section.

n/a	Confirmed	
☐	☒	The exact sample size (*n*) for each experimental group/condition, given as a discrete number and unit of measurement
☐	☒	A statement on whether measurements were taken from distinct samples or whether the same sample was measured repeatedly
☐	☒	The statistical test(s) used AND whether they are one- or two-sided<br>*Only common tests should be described solely by name; describe more complex techniques in the Methods section.*
☒	☐	A description of all covariates tested
☒	☐	A description of any assumptions or corrections, such as tests of normality and adjustment for multiple comparisons
☐	☒	A full description of the statistical parameters including central tendency (e.g. means) or other basic estimates (e.g. regression coefficient) AND variation (e.g. standard deviation) or associated estimates of uncertainty (e.g. confidence intervals)
☐	☒	For null hypothesis testing, the test statistic (e.g. *F*, *t*, *r*) with confidence intervals, effect sizes, degrees of freedom and *P* value noted<br>*Give P values as exact values whenever suitable.*
☒	☐	For Bayesian analysis, information on the choice of priors and Markov chain Monte Carlo settings
☒	☐	For hierarchical and complex designs, identification of the appropriate level for tests and full reporting of outcomes
☐	☒	Estimates of effect sizes (e.g. Cohen's *d*, Pearson's *r*), indicating how they were calculated

Our web collection on statistics for biologists contains articles on many of the points above.

Software and code

Policy information about availability of computer code

Data collection	LAS AF Lite, cyt Expert
Data analysis	All data were processed in GraphPad Prism (version 8.0 for Windows) by One-Way ANOVA or Two-Way ANOVA test. Standard curve calibration was done using OriginPro (version 9.0 for windows). Figures are created by 3Ds-MAX 2020 and Powerpoint 2020.

For manuscripts utilizing custom algorithms or software that are central to the research but not yet described in published literature, software must be made available to editors and reviewers. We strongly encourage code deposition in a community repository (e.g. GitHub). See the Nature Portfolio guidelines for submitting code & software for further information.

Data

Policy information about availability of data

All manuscripts must include a data availability statement. This statement should provide the following information, where applicable:

- Accession codes, unique identifiers, or web links for publicly available datasets
- A description of any restrictions on data availability
- For clinical datasets or third party data, please ensure that the statement adheres to our policy

The expression of NQO1 in RBCA and peri-tumor healthy tissues in this study is obtained from the TCGA and GTEx databases and recomputed using UCSC Xena online analysis system [https://xena.ucsc.edu/]. The accessible links/accession-codes for bioagents and cells could be obtained from the http://gepia2.cancer-pku.cn/#analysis. The authors declare that all data supporting the findings of this study are available within the article and its supplementary information files. The original data have been submitted as a Source Data file. A reporting summary for this article is available as a Supplementary Information file.

Field-specific reporting

Please select the one below that is the best fit for your research. If you are not sure, read the appropriate sections before making your selection.

☒ Life sciences ☐ Behavioural & social sciences ☐ Ecological, evolutionary & environmental sciences

For a reference copy of the document with all sections, see nature.com/documents/nr-reporting-summary-flat.pdf

Life sciences study design

All studies must disclose on these points even when the disclosure is negative.

Sample size	Sample size was determined based on the set-ups in previous reports to ensure adequate power (Y. Lee et al., Nat Mater 19, 118-126 (2020) and P. Praveschotinunt et al., Nat Commun 10, 5580 (2019)).
Data exclusions	No data exclusion was performed.
Replication	Individual tests have at least three independent replicates to ensure data consistency.
Randomization	Samples and cells were randomly allocated into groups. Mice with comparable age, weight and tumor sizes were randomly selected from the housing cages and then divided into experimental groups with no bias for further treatment.
Blinding	Investigators were blinded to group allocation during experiments.

Reporting for specific materials, systems and methods

We require information from authors about some types of materials, experimental systems and methods used in many studies. Here, indicate whether each material, system or method listed is relevant to your study. If you are not sure if a list item applies to your research, read the appropriate section before selecting a response.

Materials & experimental systems

n/a	Involved in the study	
☐	☒	Antibodies
☐	☒	Eukaryotic cell lines
☒	☐	Palaeontology and archaeology
☐	☒	Animals and other organisms
☒	☐	Human research participants
☒	☐	Clinical data
☒	☐	Dual use research of concern

Methods

n/a	Involved in the study	
☒	☐	ChIP-seq
☐	☒	Flow cytometry
☒	☐	MRI-based neuroimaging

Antibodies

Antibodies used	Antibodies used in this study include: GADPH (Proteintech, 10494-1-AP, 1:10000), BAX (Proteintech, 50599-2-Ig, 1:1000), Bcl-2 (Proteintech, 26593-1-AP, 1:1000), HMGB1 (Proteintech, 66525-1-Ig, 1:1000), CRT (Proteintech, 10292-1-AP, 1:1000), HSP70 (Proteintech, 66183-1-Ig, 1:1000), NQO1 (Proteintech, 11451-1-AP, 1:5000), HRP-conjugated anti-rabbit secondary antibody (Proteintech, SA00001-2, 1:5000), Cy3-conjugated Goat anti-Rabbit IgG (Sangon Biotech, D110062, 1:300), Alexa Fluor 488-conjugated Goat anti-rabbit IgG (Sangon Biotech, D110061, 1:300). STING (Cell Signaling Technology, 13647, 1:1000), p-STING(Cell Signaling Technology, 50907, 1:1000), TBK1 (Cell Signaling Technology, 3504, 1:1000), p-TBK1 (Cell Signaling Technology, 5483T, 1:1000), IRF3 (Cell Signaling Technology, 29047, 1:1000), p-IRF3 (Cell Signaling Technology, 29047, 1:1000), IRF3 (Cell Signaling Technology, 4302, 1:1000). CD4 (Leinco Technologies, 53-6.7 C375), CD8 (Leinco Technologies, GK1.5 C1333).<br>Fluorescent antibodies used in this study include: FITC-antiCD80 (Biolegend, 16-10A1, 50µg), PE-antiCD86 (Biolegend, A17199A, 50µg), APC-antiF4/80 (Biolegend, QA17A29, 100µg), FITC-antiCD4 (Biolegend, KG1.5, 50µg), PE-antiCD8 (Biolegend, S18018E, 50µg), FITC-antiCD8(Biolegend, 53-6.7), FITC-antiIFN-γ(Biolegend,XMG1.2) and APC-antiCD11c (Biolegend, N418, 100µg).<br>Antibodies for western blot analysis were diluted Primary Antibody Dilution Buffer.<br>Antibodies for intraperitoneal injections were diluted using sterile PBS.
Validation	All antibodies in the study were freshly obtained from the manufacturers and used according to the user manuals. All validation statements can be found on the respective antibody website:<br>1.GADPH:https://www.ptgcn.com/products/GAPDH-Antibody-10494-1-AP.htm<br>2.BAX:https://www.ptgcn.com/products/BAX-Antibody-50599-2-Ig.htm<br>3.Bcl-2:https://www.ptgcn.com/products/Bcl2-Antibody-26593-1-AP.htm<br>4.HMGB1:https://www.ptgcn.com/products/HMGB1-Antibody-66525-1-Ig.htm<br>5.CRT:https://www.ptgcn.com/products/CALR-Antibody-10292-1-AP.htm

6.HSP70:https://www.ptgcn.com/products/HSP70-Antibody-66183-1-Ig.htm
7.NQO1:https://www.ptgcn.com/products/NQO1-Antibody-11451-1-AP.htm
8.HRP-conjugated anti-rabbit secondary antibody:https://www.ptgcn.com/products/HRP-conjugated-Affinipure-Goat-Anti-Rabbit-IgG-H-L-secondary-antibody.htm
9.Cy3-conjugated Goat anti-Rabbit IgG:https://www.sangon.com/productDetail?productInfo.code=D110062
10.Alexa Fluor 488-conjugated Goat anti-rabbit IgG:https://www.sangon.com/productDetail?productInfo.code=D110061
11.STING:https://www.cellsignal.cn/products/primary-antibodies/sting-d2p2f-rabbit-mab/13647?site-search-type=Products&N=4294956287&Ntt=sting&fromPage=plp
12.p-STING:https://www.cellsignal.cn/products/primary-antibodies/phospho-sting-ser366-e9a9k-rabbit-mab/50907?site-search-type=Products&N=4294956287&Ntt=sting&fromPage=plp
13.TBK1:https://www.cellsignal.cn/products/primary-antibodies/tbk1-nak-d1b4-rabbit-mab/3504?site-search-type=Products&N=4294956287&Ntt=tbk1&fromPage=plp
14.p-TBK1:https://www.cellsignal.cn/products/primary-antibodies/phospho-tbk1-nak-ser172-d52c2-xp-rabbit-mab/5483?site-search-type=Products&N=4294956287&Ntt=p-tbk1&fromPage=plp
15.p-IRF3:https://www.cellsignal.cn/products/primary-antibodies/phospho-irf-3-ser396-d6o1m-rabbit-mab/29047?site-search-type=Products&N=4294956287&Ntt=irf3&fromPage=plp
16.CD4:https://www.leinco.com/p/anti-mouse-cd4-clone-gk1-5-purified-functional-grade-gold/
17.CD8:https://www.leinco.com/p/anti-mouse-cd8a-ly-2-purified-functional-grade-gold/
18.FITC anti-mouse IFN-γ Antibody:https://www.biolegend.com/en-us/products/fitc-anti-mouse-ifn-gamma-antibody-995
19.FITC-antiCD80:https://www.biolegend.com/en-us/products/fitc-anti-mouse-cd80-antibody-41
20.PE-antiCD86:https://www.biolegend.com/en-us/products/pe-anti-mouse-cd86-antibody-18945
21.APC-antiF4/80:https://www.biolegend.com/en-us/products/apc-anti-mouse-f4-80-recombinant-antibody-18756
22.FITC-antiCD4:https://www.biolegend.com/en-us/products/fitc-anti-mouse-cd4-antibody-248
23.PE-antiCD8:https://www.biolegend.com/en-us/products/pe-anti-mouse-cd8a-antibody-20990
24.FITC-antiCD8:https://www.biolegend.com/en-us/products/fitc-anti-mouse-cd8a-antibody-153
25.FITC-antiIFN-γ:https://www.biolegend.com/en-us/products/fitc-anti-mouse-ifn-gamma-antibody-995
26.APC-antiCD11c:https://www.biolegend.com/en-us/products/apc-anti-mouse-cd11c-antibody-1813

Eukaryotic cell lines

Policy information about cell lines

Cell line source(s)
4T1 and 4T1-Luc cell lines were purchased from Yeze Shanghai Biological Technology Co. LTD. under the catalog number of CRL-2539 and CRL-2539-luc2, respectively.B16F10 and B16F10-Luc were provided by Chongqing Medical University under the catalog number of CRL-6475 and CRL-6475-luc2.

Authentication
These cell lines were authenticated by the supplier using STR analysis.

Mycoplasma contamination
No contamination was detected by the supplier using Hoechst DNA stain method, agar culture method and PCR-based assay.

Commonly misidentified lines (See ICLAC register)
Name any commonly misidentified cell lines used in the study.

Animals and other organisms

Policy information about studies involving animals; ARRIVE guidelines recommended for reporting animal research

Laboratory animals
Male STING-KO C57 mice with an average body weight of 15±2g and age of 6 weeks were purchased from Gempharmatech Co., LTD. BALB/c and C57 mice (male, 6-week-old) were provided by Chongqing Medical University. All mice were kept in the animal house of Chongqing Medical University. Mice were housed in cages with four mice per cage and kept on in a regular 12-h:12-h light:dark cycle (9:00 AM-9:00 PM; 9:00 AM-9:00 PM). The temperature was 22±1 degree Celsius) and humidity was 40%-68%.

Wild animals
No wild animals were used in the study.

Field-collected samples
No field collected samples were used in the study.

Ethics oversight
The animal experiments in the present study were overseen by the Animal Care and Use Committee of Laboratory Animals Administration of Chongqing Medical University.

Note that full information on the approval of the study protocol must also be provided in the manuscript.

Flow Cytometry

Plots

Confirm that:

☒ The axis labels state the marker and fluorochrome used (e.g. CD4-FITC).

☒ The axis scales are clearly visible. Include numbers along axes only for bottom left plot of group (a 'group' is an analysis of identical markers).

☒ All plots are contour plots with outliers or pseudocolor plots.

☒ A numerical value for number of cells or percentage (with statistics) is provided.

Methodology

Sample preparation	In vitro flow cytometry analysis: Immune cells with different treatments were collected after digestion and centrifuged at low speed. Cells were then washed by PBS, followed by incubation with 3µL FITC-CD4 antibody and 3µL PE-CD8 antibody or 3µL FITC-CD80 antibody and 3uL PE-CD86 antibody in 200µL staining buffer.<br>In vivo: Murine tumor tissues, spleen and lymph nodes were grinded in a sterile environment, followed by digestion with red blood cell lysis buffer for 5min. The collected cells were washed by PBS and then incubated with 3µL APC-CD3, 3µL FITC-CD4 and 3µL PE-CD8 , 3µL APC-CD3, 3µL FITC-CD8a and 3µL PE-IFN-γ, 3µL APC-F4/80, 3µL FITC-CD80/CD206 and 3µL PE-CD45 , 3µL APC-CD11c, 3µL FITC-MHC-I and 3µL PE-MHC II or 3µL APC-CD11c, 3µL FITC-CD80 and 3µL PE-CD86 in 200µL staining buffer.
Instrument	Analytical Flow cytometry (CytoFLEX)
Software	FlowJo-V10 and CytExpert
Cell population abundance	The cells stained with different markers were filtered using 300 screen mesh cell strainer. 10000 cells were extracted from individual samples for flow cytometry analysis.
Gating strategy	Living cells are first gated on the basis of their scattering properties using forward (FSC) and side scatter (SSC). Adhered particles and impurities were removed via FSCA/FSCH approach. Immune cells were gated via the APC/SSCA approach to specify DC or T cells, while activated immune cells were gated via CD80/CD86 ,F4/80/CD86, F4/80/CD206, MHC I/MHC II, CD4/CD8 or CD8a/IFN-γ expressions.

☒ Tick this box to confirm that a figure exemplifying the gating strategy is provided in the Supplementary Information.

March 2021
